# The Association Between High Birth Weight and Long-Term Outcomes—Implications for Assisted Reproductive Technologies: A Systematic Review and Meta-Analysis

**DOI:** 10.3389/fped.2021.675775

**Published:** 2021-06-23

**Authors:** Åsa Magnusson, Hannele Laivuori, Anne Loft, Nan B. Oldereid, Anja Pinborg, Max Petzold, Liv Bente Romundstad, Viveca Söderström-Anttila, Christina Bergh

**Affiliations:** ^1^Department of Obstetrics and Gynaecology, Institute of Clinical Sciences, Sahlgrenska University Hospital, Sahlgrenska Academy, Gothenburg University, Gothenburg, Sweden; ^2^Department of Obstetrics and Gynecology, Tampere University Hospital and Faculty of Medicine and Health Technology, University of Tampere, Tampere, Finland; ^3^Medical and Clinical Genetics, University of Helsinki and Helsinki University Hospital, Helsinki, Finland; ^4^Institute for Molecular Medicine Finland, Helsinki Institute of Life Science, University of Helsinki, Helsinki, Finland; ^5^Fertility Clinic, Rigshospitalet, Copenhagen University Hospital, Copenhagen, Denmark; ^6^Livio IVF-klinikken Oslo, Oslo, Norway; ^7^Swedish National Data Service & Health Metrics Unit, University of Gothenburg, Gothenburg, Sweden; ^8^Spiren Fertility Clinic, Trondheim, Norway; ^9^Centre for Fertility and Health, Norwegian Institute of Public Health, Oslo, Norway; ^10^University of Helsinki, Helsinki, Finland

**Keywords:** assisted reproduction, frozen embryo transfer, large for gestational age, high birth weight, long-term morbidity, cancer, diabetes

## Abstract

**Background:** Studies have shown that the prevalence of children born with high birth weight or large for gestational age (LGA) is increasing. This is true for spontaneous pregnancies; however, children born after frozen embryo transfer (FET) as part of assisted reproductive technology (ART) also have an elevated risk. In recent years, the practice of FET has increased rapidly and while the perinatal and obstetric risks are well-studied, less is known about the long-term health consequences.

**Objective:** The aim of this systematic review was to describe the association between high birth weight and LGA on long-term child outcomes.

**Data Sources:** PubMed, Scopus, and Web of Science were searched up to January 2021. Exposure included high birth weight and LGA. Long-term outcome variables included malignancies, psychiatric disorders, cardiovascular disease, and diabetes.

**Study Selection:** Original studies published in English or Scandinavian languages were included. Studies with a control group were included while studies published as abstracts and case reports were excluded.

**Data Extraction:** The methodological quality, in terms of risk of bias, was assessed by pairs of reviewers. Robins-I (www.methods.cochrane.org) was used for risk of bias assessment in original articles. For systematic reviews, AMSTAR (www.amstar.ca) was used. For certainty of evidence, we used the GRADE system. The systematic review followed PRISMA guidelines. When possible, meta-analyses were performed.

**Results:** The search included 11,767 articles out of which 173 met the inclusion criteria and were included in the qualitative analysis, while 63 were included in quantitative synthesis (meta-analyses). High birth weight and/or LGA was associated with low to moderately elevated risks for certain malignancies in childhood, breast cancer, several psychiatric disorders, hypertension in childhood, and type 1 and 2 diabetes.

**Conclusions:** Although the increased risks for adverse outcome in offspring associated with high birth weight and LGA represent serious health effects in childhood and in adulthood, the size of these effects seems moderate. The identified risk association should, however, be taken into account in decisions concerning fresh and frozen ART cycles and is of general importance in view of the increasing prevalence in high birthweight babies.

## Introduction

The association between preterm birth (PTB), low birth weight (LBW), and small for gestational age (SGA) and neonatal and long-term outcomes is well-described and suggests higher risks for cardiovascular diseases, diabetes, hypertension, and stroke later in life according to the Barker hypothesis (1). Less attention has been paid to high birthweight children and children born large for gestational age (LGA), particularly the long-term outcomes. The prevalence of high birthweight and LGA babies is increasing (2, 3), in parallel with the worldwide rise in obesity, also among women of childbearing age (3). In assisted reproduction, several studies have shown that children born after transfer of frozen/thawed embryos (FET) have a lower risk of preterm birth, low birth weight, and SGA compared with singletons born after fresh transfer but also a higher risk of being born with a high birth weight and LGA (4–6). Due to high success rates, FET of vitrified/warmed blastocysts has increased dramatically in recent years, including the “freeze all” technique where all available embryos of good quality are cryopreserved for later use in a natural or programmed cycle (7–11). The perinatal outcomes for babies of high birth weight and being LGA are mainly associated with difficulties at delivery such as asphyxia, shoulder dystocia, hypoglycemia, respiratory problems, cesarean section, and obstetric injuries (12, 13). For long-term outcomes, an association has been found between high birth weight and child malignancies, breast cancer, psychiatric disorders, and cardiometabolic diseases (14–19).

The aim of this systematic review and meta-analysis is to summarize the present knowledge on long-term outcomes for children born with a high birth weight or being LGA.

## Methods

We searched PubMed, Scopus, and Web of Science databases up to January 2021. Exposures were large for gestational age and high birth weight. Long-term morbidity outcomes studied were cancer, metabolic disease, cardiovascular disease, and psychiatric disorders. Cancer was focused on breast cancer, child malignancies in the central nervous system (CNS), hematological malignancies, and Wilm's tumor. Metabolic diseases were focused on diabetes type 1 and type 2. Cardiovascular disease was focused on hypertension and other cardiovascular disorders. Psychiatric disorders were focused on schizophrenia/psychosis and cognitive disorders. Some of these outcomes, when appropriate, were used for meta-analysis.

### Systematic Search for Evidence

The terms used in the searches are listed below:

LGA[tiab] OR large for gestational age[tiab] OR large-for-gestational age[tiab] OR HBW[tiab] OR high birth weight^*^[tiab] OR higher birth weight^*^[tiab] OR highest birth weight^*^[tiab] OR high birthweight^*^[tiab] OR higher birthweight^*^[tiab] OR highest birthweight^*^[tiab] OR macrosomia[tiab]. Because of large heterogenecity in the nomenclature of diseases and to avoid missing any important morbidity, we decided not to include any specific disease or morbidity terms in the search.

We also manually searched reference lists of identified articles for additional references. Guidelines for meta-analysis and systematic reviews (SR) of observational studies were followed (20). The literature search was performed by two researchers (Å.M. and C.B.) and one librarian. Screening of abstracts and of full papers for inclusion was done by pairs of reviewers. Differences of opinion in the team were solved by discussion until consensus was achieved.

The last literature search was performed January 14, 2021.

### Inclusion and Exclusion of Studies

Original studies published in English or Scandinavian languages were included. In the case of double publication, the latest study was included. Studies with a control group were included. Studies published only as abstracts and case reports were excluded.

### Definitions

High birth weight was defined by each author but usually ≥4,000 or ≥4,500 or occasionally >5 g. LGA was defined by each author.

### Appraisal of Certainty of Evidence

The methodological quality of original studies, in terms of risk of bias, was assessed by pairs of reviewers by the tool Robins-I (http://www.methods.cochrane.org). For systematic reviews, we used AMSTAR (http://www.amstar.ca). For certainty of evidence, we used the GRADE system (21). The systematic review followed PRISMA guidelines (22).

### Data Synthesis

Outcomes are given in odds ratio (OR), adjusted odds ratio (AOR), hazard ratio (HR), adjusted hazard ratio (AHR), relative risk (RR), adjusted relative risk (ARR), incidence rate ratio (IRR), adjusted incidence rate ratio (AIRR), standardized incidence ratio (SIR), or random-effects odds ratio (REOR) with 95% CIs. Meta-analyses were performed despite significant heterogeneity in reference groups and despite the fact that outcomes were given in AOR, ARR, or ROR. However, studies reporting estimates as HR, AHR, AIRR, and SIR were not mixed with the RR- and OR-based outcomes. The HR- and IR-based outcomes were also too few to be included in a separate meta-analysis. A random-effects meta-analysis using the Der Simonian and Laird method, with the estimate of heterogeneity being taken from the Mantel–Haenszel model, was used in the analysis (command metan in Stata 15).

## Results

**Figure 1:**
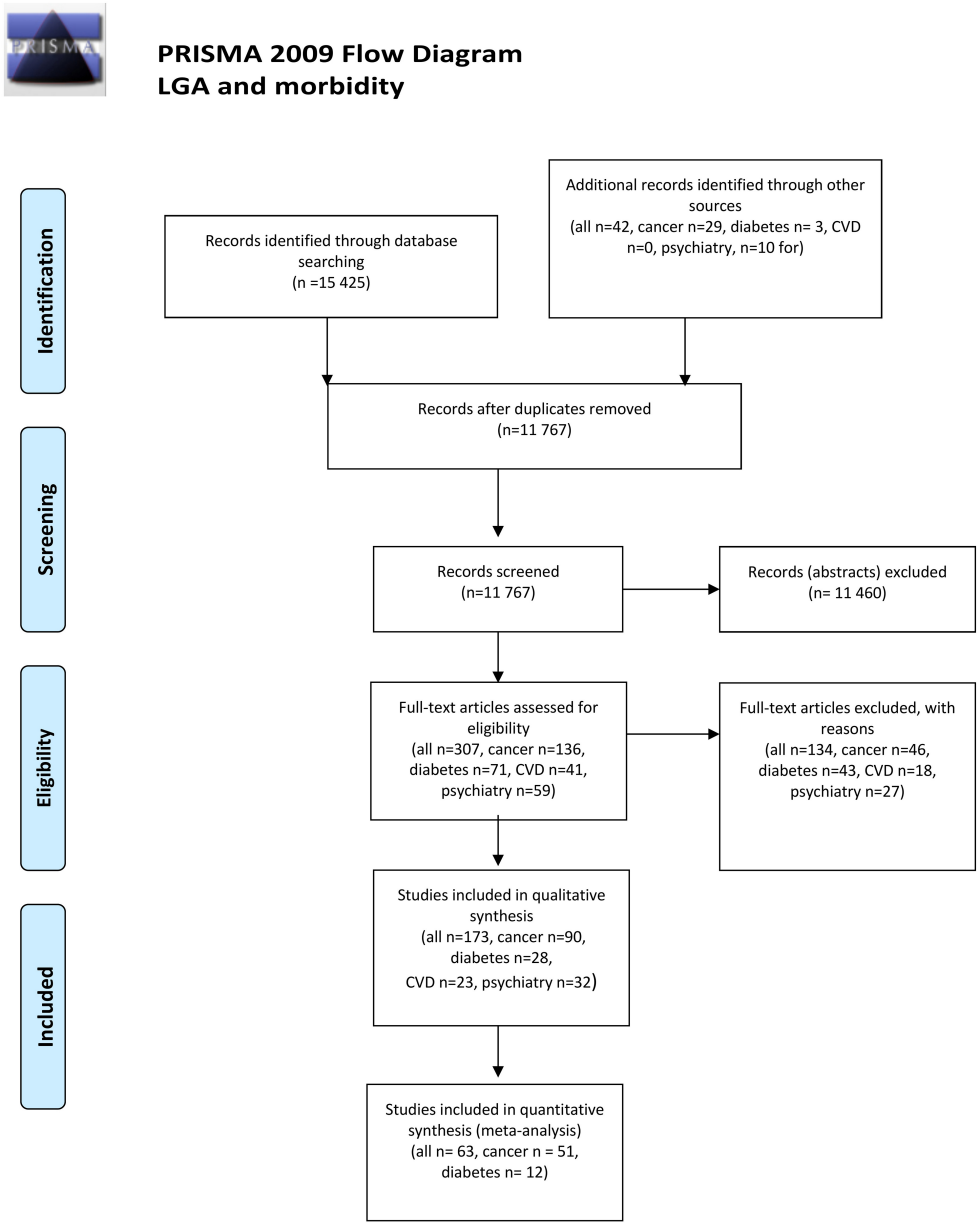
PRISMA flow chart. From Moher et al. (22). For more information, visit www.prisma-statement.org.

The search strategy identified a total of 11,767 abstracts, of which 173 were selected for inclusion in the systematic review and 63 for inclusion in quantitative synthesis (meta-analysis) (Figure 1). No papers, particularly focusing in children with high birth weight born after FET, were identified.

Among the studies included were 19 meta-analyses, 73 cohort studies, 74 case–control studies, and seven cross-sectional studies (tables, characteristics of included studies and excluded studies, with reasons for exclusion, are presented in Supplementary Tables 1.1–1.4, 2.1–2.4).

A quality assessment of the cohort, case–control, and cross-sectional studies included is presented in Supplementary Tables 3.1–3.4 and for systematic reviews in Supplementary Table 4. Of the selected cohort, case–control, and cross-sectional studies, 28 articles had low, 79 had moderate, 47 had serious, and two had critical risk of bias. Of the systematic reviews, 10 were of high, five of medium, and four were of low quality. Summary of findings (SoF) is presented in Supplementary Table 5.

### Malignancies

Outcomes are listed in Table 1.1.

**Table 1.1:** LGA and high birth weight and long-term outcomes—malignancies.

**Author, year, country**	**Study design**	**Cases**	**Outcomes (risk estimates)**	**Reference group (weight)**	**Comments/adjustments**	**Risk of bias**	**Directness**	**Precision**
**Breast cancer** **Systematic reviews/meta-analyses** ***n*** **=** **3**								
Michels and Xue (2006), USA (21)	• Meta-analysis • Cohort *n* = 11 • Case–control *n* = 16	12,301	• Birth weight >4,000 g (one study >3,000 g) • Cohort studies OR/HR/SIR 1.24 (95% CI 1.10–1.40) • Case–control studies OR/HR/SIR 1.21 (95% CI 1.06–1.38) • Total RR 1.23 (95% CI 1.13–1.24)	<2,500 g	Partly overlap with Xue (24)			
Xue and Michels (2007), USA (23)	• Cohort *n* = 14 Case–control *n* = 18 • Systematic review, meta-analysis	21,845	RR with increased birth weights 1.15 (1.09–1.21)		• Partly overlap (23) • The association disappeared after adjustment for birth length			
Zhou et al. (2020), China (24)	• Case/control *n* = 16 • Systematic review, meta-analysis	16,000	• RR per 500 g increase in birth weight • All ages: 1.02 (95% CI 1.01–1.03) • Pre-menopausal RR 1.09 (95% CI 1.04–1.15)					
**Breast cancer** **Original articles** ***n*** **=** **19**								
Andersson et al. (2001), Sweden (25) • All cancers	Cohort *n* = 1,080	62	Birth weight 4,000–5,500 g RR 1.57 (95% CI 0.67–3.64)	1,600–3,000 g	Adjusted for cohort membership, gestational age	Serious	Good	Poor
Ahlgren et al. (2003), Denmark (26)	Cohort *n* = 106,504	2,334	• Risk increase 8% per 1,000 g increase in birth weight (95% CI 1–16%) • Birth weight >5,000 g RR 1.2	3,000–3,399 g	Adjustments for age and calendar period	Moderate	Good	Good
Ahlgren et al. (2004), Denmark (27)	Cohort *n* = 117,415	3,340	• Weight category 4,000 g (median) • RR 1.17 (95% CI 1.02–1.33)	2,500 g (median)	Adjustments for attained age, calendar period, age of first childbirth and parity	Moderate	Good	Good
Ahlgren et al. (2007), Denmark (28)	Cohort >200,000 men and women	3,066	RR for trend 1.05 (95% CI 0.98–1.12)	3,000–3,499 g	Adjustment for age and calendar period	Moderate	Good	Good
Barber et al. (2019), USA (29)	Cohort *n* = 20,959	601	Birth weight >4,000 g HR 1.26 (95% CI 0.97–1.63)	2,500–3,999 g	Adjustments for time period, age, parity, age at first birth and family history of breast cancer	Serious	Good	Fair
dos Santos et al. (2004), UK (30)	Cohort *n* = 2,176	59	Birth weight≥4,000 g ARR 1.57 (95% CI 0.60–4.13)	<3,000 g	Adjusted for age	Moderate	Good	Poor
Innes et al. (2000), USA (14)	Case–control	484	Birth weight >4,500 g AOR 3.10 (95% CI 1.18–7.97)	2,500–3,499 g	Adjustments for gestational age, preeclampsia, abruptio placentae, multiple gestation, parity (birth rank), number of previous births, maternal age, paternal age, and race	Serious	Good	Poor
Lahmann et al. (2004), Sweden (35)	Case–control	89	Birth weight >4,000 g AOR 2.66 (95% CI 0.96–7.41)	<3,000 g	Adjustments for gestational age, birth year, pre-eclampsia, parental occupation, adult BMI, and educational attainment	Serious	Good	Poor
McCormack et al. (2003), Sweden (31)	Cohort *n* = 5,358	359	• Birth weight >4,000 g Premenopausal (<50 years) RR 3.48 (95% CI 1.29–9.38) • Postmenopausal (>50 years) RR 0.87 (95% CI 0.56–1.36)	<3,000 g	Adjustments for gestational age, marital status, children in home, age at first marriage, level of education, occupation, car possession	Low	Good	Fair
Mellemkjær et al. (2003), Denmark (36)	Case–control	881	Birth weight ≥4,000 g AOR 1.25 (95% CI 1.00–1.55)	3,000–3,499 g	Adjustments for marital status, birth order, maternal age at birth	Moderate	Good	Good
Michels et al. (1996), USA (37)	Case–control	582	Lower birth categories had significantly lower OR. Example 3,000–3,499 AOR 0.68 (95% CI 0.48–0.97)	>4,000	Adjustments for age, parity, cohort, age at first birth, age at menarche, BMI and family history of breast cancer	Serious	Good	Good
Michels and Xue (2006), USA, (21)	• Longitudinal cohort • *n* = 152,608	3,140	Lower weight categories had significantly lower HR. Example HR 0.66 (95% CI 0.47–0.93) if <2,495 g	>3,815 g	Adjustments for age, premature birth, age at menarche, BMI at age 18, current BMI, family history of breast cancer, history of benign breast disease, age at first birth, oral contraceptive use, physical activity, and alcohol consumption	Low	Good	Good
Mogren et al. (1999), Sweden (33)	• Cohort • *n* = 248,701	57	• High birth weight, >4,500 g • SIR 7.35 (95% CI 0.10–40.87)		Sex, age, calendar-specific person-year	Low	Good	Poor
Sanderson et al. (2002), USA (38)	Case–control	288	• High birth weight ≥4,000 g • AOR 0.7 (95% CI 0.4–1.4)	2,500–2,999 g	• Total 1,459 breast cancer, premenopausal interviewed, *n* = 288/296 • Adjusted for age, income, family history of breast cancer, history of fibroid adenoma, age at menarche, parity, age at first live birth	Moderate	Fair	Fair
Troisi et al. (2013), Sweden, Norway, Denmark (39)	Case–control	1,419	• Birth weight ≥4,000 g RR 1.14 (95% CI 0.98–1.34) • Continuous per 500 g RR 1.07 (95% CI 1.02–1.13)	2,500–3,999 g	Adjusted for gestational length	Low	Good	Good
Titus-Ernstoff et al. (2002), USA (40)	Case–control	5,659	Birth weight ≥4,500 g OR 1.18 (95% CI 0.92–1.51)	3,000–3,499 g	Adjustments for BMI at reference date, Jewish/non-Jewish, family history of breast cancer, age at first birth, parity, age at menopause	Serious	Good	Fair
Vatten et al. (2002), Norway (41)	Case–control	373	Birth weight >3,730 g OR 1.4 (95% CI 1.1–1.9)	<3,090 g	Adjustments for age at first birth and parity	Low	Fair	Fair
Vatten et al. (2005), Norway (34)	• Cohort • *n* = 16,016	312	Birth weight >3,840 g RR 1.5 (95% CI 1.0–2.2)	<3,040 g	Adjustments for year of birth, gestational length, marital status, socioeconomic status, maternal age, and birth order	Moderate	Good	Fair
Wu et al. (2011), USA (42)	Case–control	2,259	Birth weight ≥4,000 g OR 1.97 (95% CI 1.15–3.39)	<2,500 g	Adjustment for age, age at menarche, parity, adult BMI, Asian ethnicity, interviewer, years in USA, menopausal status, age at menopause, total calories, physical activity, and family history of breast cancer	Serious	Poor	Fair
• **CNS tumors** • **Systematic reviews/meta-analyses** • *n* = **4**								
Dahlhaus et al. (2016), Germany (43)	• Systematic review • Cohort *n* = 3 • Case–control *n* = 11	18,845	• >4,000 g • Astrocytoma REOR 1.60 (96% CI 1.23–2.09) • Ependymoma REOR 1.18 (95% CI 0.97–1.43) • Medulloblastoma REOR 1.31 (95% CI 1.08–1.58)	<4,000 g	Different adjustments in different studies			
Georgakis et al. (2017), Greece (45)	• Systematic review and MA • Cohort *n* = 9 • Case–control *n* = 32	53,167	• CNS tumors overall • >4,000 g OR 1.14 (95% CI 1.08–1.20) • LGA OR 1.12 (95% CI 1.03–1.22)	<4,000 g AGA	Only child cases *n* = 22,330 I meta-analyses			
Harder et al. (2008), Germany (44)	• Meta-analysis • Cohort *n* = 2 • Case–control *n* = 6	3,665	• >4,000 g • Astrocytoma OR 1.38 (95% CI 1.07–1.79) • Medulloblastoma OR 1.27 (95% CI 1.02–1.60)	<4,000 g				
Harder et al. (2010), Germany (47)	• Meta-analysis • Cohort *n* = 1 • Case–control *n* = 10	3,004	• >4,000 g OR 1.19 (95% CI 1.04–1.36)	<4,000 g				
**CNS tumors** **Original articles** *n* = **18**								
Crump et al. (2015), Sweden (46)	• Cohort • *n* = 3,571,574	2,809	• Birth weight ≥4,000 g • IRR 1.13 (95% CI 1.03–1.25)	2,500–3,999 g	Adjusted for year of birth both continuous and categorical, gender, fetal growth, parental country of birth, maternal education, familiar history of brain tumor in parents or siblings	Low	Good	Good
Emerson et al. (1991), USA (186)	Case–control	157	• Birth weight >4,000 g All histologies • AOR 1.4 (95% CI 1.0–2.0)	<4,000 g	Adjustments for matching variables; county of birth and birth year	Moderate	Good	Fair
Greenop et al. (2014), Australia (180)	Case–control	319	• Birth weight >4,000 g AOR 0.9 (95% CI 0.8–1.0) • LGA AOR 0.8 (95% CI 0.5–1.2)	2,500–3,999 g AGA	Adjusted for maternal age, year of birth, ethnicity, maternal folate supplementation	Serious	Good	Fair
Johnson et al. (2016), USA (190)	Cross-sectional	184	• Birth weight >3,915–5,815 g • HR 1.38 (95% CI 0.85–2.26)	<3,020 g	Adjusted for gestational age category	Moderate	Poor	Poor
Kitahara et al. (2014), Denmark (48)	• Cohort • *n* = 320,425	608	HR 1.13 (95% CI 1.04–1.24) per 0.5 kg increase in birth weight		No adjustments	Low	Good	Good
Mallol-Mesnard et al. (2008), France (183)	Case–control	209	Birth weight >4,000 g AOR 1.0 (95% CI 0.5–1.7)	2,500–4,000 g	Matched for age and sex	Moderate	Good	Fair
McLaughlin et al. (2009), USA (181)	Case–control	529	Birth weight ≥4,000 g RR1.4 (95% CI 0.7–2.5)	2,500–3,499 g	Adjustments for birth year, region, gender, race and birth weight	Moderate	Good	Poor
Oksuzyan et al. (2013), USA (184)	Case–control	3,308	• Birth weight >4,000 g AOR 1.12 (95% CI 0.91–1.38) • LGA AOR 1.09 (95% CI 0.89–1.27)	2,500–4,000 g	Adjusted for race, gestational age, birth order, maternal age, father's education, and source of payment for delivery	Moderate	Good	Fair
O'Neill et al. (2015), USA+UK (50)	Case–control	3,561, 5,702	• Birth weight per 0.5 kg increase • AOR 1.05 (95% CI 1.01–1.08) • AOR 1.07 (95% CI 1.04–1.10) • Birth weight ≥4,000 g • AOR 1.18 (95% CI 1.06–1.32) • AOR 1.14 (95% CI 0.98–1.34)	Per 500-g increase, 3,000–3,490 g	Adjusted for maternal age, plurality, gender, state and year of birth, birth order, maternal ethnicity	Moderate	Good	Good
Savitz and Ananth (1994), USA (64)	Case–control	47	Birth weight > 4,000 g OR 2.3 (95% CI 0.9–6.0)	2,500–4,000 g	Adjusted for year of diagnosis	Serious	Good	Poor
Schüz et al. (2001), Germany (81)	Case–control	466	• Birth weight >4,000 g • OR 1.31 (95% CI 0.97–1.78)	2,500–4,000 g	Adjustments for gender, age group of 1 year, year of birth, degree of urbanization and socioeconomic status	Serious	Good	Fair
Schüz and Forman (2007), Germany (65)	Case–control	389	• Birth weight >4,000 g • AOR 1.34 (95% CI 0.97–1.85) • LGA AOR 1.18 (95% CI 0.80–1.72)	2,500–4,000 g	Stratified for gender and age, adjusted for urbanization and socioeconomy	Serious	Good	Fair
Spix et al. (2009), Germany (196)	Case–control	• Leukemia • Cases = 229 • Controls = 557 • CNS • Cases = 88 • Controls = 204	• Birth weight >4,000 g Leukemia AOR 1.96 (95% CI 1.12–3.41) • CNS tumors AOR 3.55 (95% CI 0.81–15.62) <2,500 g	2,500–4,000	• Matching criteria, sex, age, and year of diagnosis • Response rate cases 78.1% and controls 61.4%	Serious	Good	Poor
Tettamanti et al. (2016), Sweden (49)	Cohort *n* = 2,032,727	758	• LGA • Glioma ARR 1.11 (95% CI 0.82–1.49) • Meningioma ARR 0.92 (95% CI 0.50–1.68)?? • Neuroma ARR 1.31 (95% CI 0.62–2.80) • Birth weight 4,000–6,000 g • Glioma ARR 1.12 (95% CI 0.86–1.47) • Meningioma ARR 0.71 (95% CI 0.40–1.28) • Neuroma ARR 0.99 (95% CI 0.49–2.01)	AGA 2,500–3,999 g	Adjustments for sex, maternal and paternal age, maternal birthplace, birth cohort, parental socioeconomic index at birth, birth weight by gestational age, head circumference, and birth length	Low	Good	Fair
Tran et al. (2017), USA (195)	Case–control	72	• Birth weight >4,000 g • AOR 2.5 (95% CI 1.2–5.2) • >4,000 g + LGA • AOR 2.7 (95% CI 1.1–6.2)	2,500–4,000 g AGA	Adjustments for sex, ethnicity, year of birth, age at diagnosis, gestational age, maternal age, and DOE sites	Moderate	Good	Poor
Urayama et al. (2007), USA (185)	Case–control	508	Birth weight >4,000 g AOR 1.22 (95% CI 0.90–1.66)	2,500–3,999 g	Adjustment for age, race, ethnicity, gestational age, birth order, abnormalities, socioeconomic factors, type of delivery	Moderate	Good	Fair
Von Behren and Reynolds (2003), USA (179)	Case–control	746	Birth weight ≥4,000 g OR 1.05 (95% CI 0.7–1.35)	2,500–3,999 g	Adjustments for birth date and sex	Moderate	Good	Fair
Yaezel et al. (1997), USA, Australia, Canada (66)	Case–control	252	Birth weight >4,000 g AOR 1.2 (95% CI 0.7–1.8)	<4,000 g	Adjusted for maternal age, birth order, gestational age, sex, maternal race, maternal/paternal education, income, age at diagnosis	Moderate	Good	Good
• **Hematologic malignancies** • **Systematic reviews** *n* = **2**								
Caughey and Michels (2009), USA (192)	SR and MA 28 case–control and 4 cohort studies	16,501	• Birth weight >4,000 g All leukemias • AOR 1.35 (96% CI 1.24–1.48)	Differs between 2,500–2,999 and <4,000 g	Different adjustments in different studies			
Hjalgrim et al. (2003), Denmark (191)	SR and MA 18 case–control studies	10,282	Birth weight >4,000 g AOR for ALL and leukemia combined OR 1.26 (95% CI 1.17–1.37)		Different adjustments in different studies			
• **Hematologic malignancies** • **Original articles** *n* = **29**								
Cnattingus et al. (1995), Sweden (77)	Case–control	613	• LL Birth weight >4,000 g • AOR 1.7 (95% CI 1.1–2.7)	3,000–3,499 g	Matched by sex and month and year of birth	Moderate	Good	Fair
Crump et al. (2015), Sweden (193)	• Cohort • *n* = 3,569,333	1,960	• ALL LGA • AIRR 1.22 (95% CI 1.06–1.40) • Birth weight >4,000 g • AIRR 1.19 (95% CI 1.06–1.32)	AGA 2,500–3,999 g	Adjusted for sex, birth year, fetal growth, parental country of birth, ALL in parent or sibling,	Low	Good	Good
Groves et al. (2018), USA (59)	Case–control	633	• ALL Birth weight >4,000 g • AOR 1.28 (95% CI 1.01–1.61)	2,500–4,000 g	Adjusted for age, sex, ethnicity, county of residence and day of birth	Moderate	Good	Good
Hjalgrim et al. (2004), Denmark, Sweden, Norway Iceland (52)	Case–control	2,204	• Birth weight ≥4,500 g • ALL AOR 1.19 (95% CI 0.09–1.58) • Trend per kg increase 1.26 (95% CI 1.13–1.41) • AML AOR 0.95 (95% CI 0.45–2.04) • Trend per kg increase 1.09 (95% CI 0.82–1.45)	3,500–3,999 g	• Matched for sex, year and month of birth • Trend adjusted for birth order, gestational age, parental age	Moderate	Good	Poor
Kaatsch et al. (1998), Tyskland (67)	Case–control	2,356	• Birth weight >4,000 g Leukemia AOR 1.64 (95% CI 1.16–2.32) • No statistics on lymphoma	2,500–4,000 g	• Matched for age, sex and place of residence at diagnosis • 81% response for cases and 67% for controls	Serious	Good	Fair
Koifman et al. (2008), Brazil (194)	Case–control	201	Birth weight >4,000 g Infant leukemia AOR 1.20 (95% CI 1.02–1.43)	2,500–2,999 g	Adjusted for sex, income, maternal age, pesticide exposure, hormonal intake during pregnancy	Serious	Good	Fair
Ma et al. (2005), USA (78)	Case–control	• 313 ALL • 53 AML	• Birth weight > 4,000 g ALL AOR 1.04 (95% CI 0.52–2.10) • AML AOR 1.60 (95% CI 0.13–19.9)	<2,500 g	Adjusted for household income, maternal education	Moderate	Good	Poor
McLaughlin et al. (2006), USA (189)	Case–control	1,070	• Birth weight ≥4,500 g • ALL AOR 1.10 (95% CI 0.67–1.73) • AML AOR 3.89 (95% CI 1.63–8.26)	3,000–3,499 g	Matched for year of birth Adjustments for year of birth, race, gender, ethnicity, maternal age, gestational age	Moderate	Good	Fair
Mogren et al. (1999), Sweden (33)	Cohort *n* = 248,701	97	• High birth weight, >4,500 g • SIR 4.29 (95% CI 1.56–9.33)		Sex, age, calendar-specific person-year	Low	Good	Fair
Okcu et al. (2002), USA (53)	Case–control	104 total leukemia83 ALL	• Leukemia total birth weight >4,000 g AOR 1.7 (95% CI 0.9–3.0) • ALL AOR 2.2 (95% CI 1.2–4.1)	2,500–4,000 g	Adjusted for year of birth, sex, gestational age, maternal age, tobacco use, parity and race	Low	Good	Moderate
O'Neill et al. (2015), USA+UK (50)	Case–control	5,561, 7,826	• Birth weight per 500 g increase • AOR 1.05 (95% CI 1.01–1.08) • AOR 1.07 (95% CI 1.04–1.10) • Birth weight ≥4,000 g • AOR 1.20 (95% CI 1.10–1.32) • AOR 1.10 (95% CI 0.96–1.26)	• Per 500 g increase • 3,000–3,490 g	Adjusted for maternal age, plurality, gender, state and year of birth, birth order, maternal ethnicity	Moderate	Good	Good
Paltiel et al. (2015), Multinational (51)	• Cohort • *n* = 112,781	• Leukemia, *n* = 115 • ALL, *n* = 98	• Birth weight >4,000 g • OR 1.31 (95% CI 0.97–1.78)	<4,000 g	Adjusted for sex, maternal age, pregnancy weight gain, BMI, first born, maternal smoking	Low	Good	Fair
Peckham-Gregory et al. (2017), USA (63)	Case–control	374 cases in total of which 89 cases with Burkitt's lymphoma	If LGA Subgroup analysis Burkitt lymphoma AOR 2.0 (95% CI 1.10–3.65)	Non-LGA	Adjusted for sex, maternal race, maternal ethnicity, year of birth, maternal education	Moderate	Poor	Poor
Petridou et al. (1997), Greece (54)	Case–control	153	Childhood leukemia AOR per 500 g increase in birth weight 1.36 (95% CI 1.04–1.77)	No ref	Matched for gender, age ±6 months, urban area	Serious	Good	Fair
Petridou et al. (2015), Sweden (62)	• Cohort • *n* = 3,444,136	684	• LGA • Non-Hodgkin lymphoma AHR 1.83 (95% CI 1.20–2.79) • Hodgkin lymphoma AHR 0.7 (95% CI 0.22–2.2) • Birth weight ≥4,000 g • Non-Hodgkin lymphoma AHR 1.10 (95% CI 0.88–1.38) • Hodgkin lymphoma AHR 1.14 (95% CI 0.78–1.67)	• 2,500–3,999 g AGA	Adjusted for sex, maternal age, maternal education, gestational age, birth order	Low	Good	Fair
Podvin et al. (2006), USA (55)	Case–control	• 376 ALL • 85 AML	• >4,000 g ALL AOR 1.6 (95% CI 1.2–2.1) • AML AOR 1.2 (95% CI 0.7–2.1)	2,500–3,999 g	Adjusted for mother's age	Moderate	Good	Good
Rangel et al. (2010), Brazil (68)	Case–control	Eligible number of cases 544. Included number of cases 410	• Birth weight ≥4,000 g • Non-Hodgkin lymphoma OR 1.99 (95% CI 1.08–3.69) • Leukemia OR 1.86 (95% CI 1.04–3.30)	<4,000 g	• Matched for gender and age • <50% responders among cases	Critical	Good	Poor
Reynolds et al. (2002), USA (56)	Case–control	• 307 ALL <2 years • 1,100 ALL 2–4 years • 240 AML	• Birth weight >4,000 g • AML OR 0.7 (95% CI 0.42–1.19) • ALL <2 years OR 0.93 (95% CI 0.63–1.39) • ALL 2–4 years OR 1.14 (95% CI 0.91–1.41)	2,500–3,999 g	No adjustments	Moderate	Good	Moderate
Robinson et al. (1987), USA (57)	Case–control	521 cases, 219 cases available for analysis	Birth weight >4,000 g ALL Relative Odds Ratio 0.73 Subgroup analysis >3,800 g and diagnosis <4 years of age OR 2.09 (95% CI 1.18–3.70)	<4,000 g	• Control group 1. Matched for date of birth and county of birth • Control group 2: year of birth • 4:1 • <50% of eligible cases identified	Serious	Good	Poor
Roman et al. (2013), USA, Germany, and UK (58)	Case–control pooled	3,922	• Weight centile >90. Boys AOR 1.2 (95% CI 1.1–1.5). Girls 1.3 (95% CI 1.1–1.6) • Per kilo increase boys 1.2 (95% CI 1.1–1.3) Girls 1.2 (95% CI 1.1–1.4) • Birth weight >4,500 g AOR 1.8 (95% CI 1.2–2.6)	3,000–3,999 g	• Controls matched for age at diagnosis • Adjusted for country, gestational age, sex, age at diagnosis • *Adjusted for sex and diagnosis • 58% of eligible controls participate	Moderate	Good	Fair
Savitz and Ananth (1994), USA (64)	Case–control	• 71 ALL • 26 lymphoma	• Birth weight > 4,000 g ALL OR 0.7 (95% CI 0.2–2.3) • Lymphoma OR 3.3 (95% CI 1.0–11.1)	2,500–4,000 g	Adjusted for year of diagnosis and maternal smoking	Serious	Good	Poor
Schüz and Forman (2007), Germany (65)	Case–control	• ALL, *n* = 621 • AML, *n* = 94 • Non-Hodgkin lymphoma, *n* = 164	• Birth weight >4,000 g • ALL AOR 1.41 (95% CI 1.08–1.84) • AML AOR 1.56 (95% CI 0.88–2.79) • Non-Hodgkin lymphoma AOR 0.94 (95% CI 0.54–1.63) • LGA • ALL AOR 1.45 (95% CI 1.07–1.97) • AML AOR 1.45 (95% CI 0.75–2.83) • Non-Hodgkin lymphoma AOR 1.40 (95% CI 0.81–2.43)	2,500–4,000 g	Stratified for gender and age, adjusted for urbanization, and socioeconomic factors	Serious	Good	Fair
Smith et al. (2009), UK (60)	Case–control	1,632	Birth weight >4,000 g AOR 1.2 (95% CI 1.02–1.43)	2,500–4,000 g	Matched for sex, month, and year of birth, area of residence	Moderate	Good	Fair
Spix et al. (2009), Germany (196)	Case–control	• Leukemia • Cases = 229 • Controls = 557 • CNS • Cases = 88 • Controls = 204	• Birth weight >4,000 g Leukemia AOR 1.96 (95% CI 1.12–3.41) • CNS tumors AOR 3.55 (95% CI 0.81–15.62) <2,500 g	2,500–4,000 g	• Matching criteria, sex, age, and year of diagnosis • Response rate cases 78.1% and controls 61.4%	Serious	Good	Poor
Tran et al. (2017), USA (195)	Case–control	207	• Birth weight >4,000 g • Leukemia AOR 1.4 (95% CI 0,7–2.6) • >4,000 g+LGA AOR 1.7 (95% CI 0.8–3.7)	• 2,500–4,000 g • AGA	Matched for year of birth, county of residence, sex, ethnicity, maternal age. Adjusted for sex, ethnicity, year of birth, age at diagnosis, gestational age, maternal age	Moderate	Good	Poor
Triebwasser et al. (2016), USA (16)	Case–control	1,216	Birth weight ≥4,000 g AOR 1.23 (95% CI 1.02–1.48)	2,500–3,999 g	Matched for month and year of birth, sex and ethnicity	Moderate	Good	Good
Westergaard et al. (1997), Denmark (76)	Cohort	• 704 ALL • 114 AML	• Birth weight 4,010–4,509 g ALL ARR 1.59 (95% CI 1.17–2.17) • AML ARR 1.66 (95% CI 0.83–3.31)	3,010–3,509 g	Adjusted for age, sex, calendar period, maternal age at birth, birth order	Low	Good	Good
Yaezel et al. (1997), USA, Australia, Canada (66)	Case–control	• ALL 1,284 • AML 185 • Non-Hodgkin lymphoma 190	• Birth weight >4,000 g ALL AOR 1.5 (95% CI 1.1–1.9) • AML AOR 1.5 (95% CI 1.0–2.4) • Non-Hodgkin lymphoma 1.5 (95% CI 1.0–2.4)	<4,000 g	Adjusted for maternal age, birth order, gestational age, sex, maternal race, maternal/paternal education, income, age at diagnosis	Moderate	Good	Good
Zack et al. (1991), Sweden (61)	Case–control	411	• Per 100-g increase in birth weight • OR 1.0 (95% CI 1.0–1.0)		Matched for sex, month, and year of birth	Moderate	Good	NA
• **Wilm's tumor** • **Systematic reviews**, *n* = **1**								
Chu et al. (2010), Canada (69)	• Systematic review, • 12 studies, cohort *n* = 3, case–control *n* = 7 and case–cohort *n* = 2	>6,000 cases	• Birth weight >4,000 g, OR 1.36 (95% CI 1.12–1.64) • LGA vs. AGA: OR 1.51 (95% CI 1.25–1.83)	2,500–4,000 g	• Case–control studies: matched for sex, year of birth, and/or year of diagnosis • Cohort studies adjusted at least for sex, year of birth. Some also adjusted for birth order, maternal age, residence., maternal education, socioeconomy			
• **Wilm's tumor** • **Original articles** *n* = **14**								
Crump et al. (2014), Sweden (70)	• Cohort • 3,571,574	443	• ≥4,000 g, girls, AHR 2.22 (95% CI 1.63–3.029) • Boys AHR 1.44 (95% CI 1.06–1.96)	2,500–3,999 g	Adjusted for age, fetal growth, gestational age at birth, birth order, maternal age, maternal education	Low	Good	Good
Daniels et al. (2008), USA (72)	Case–control	521	• ≥4,500 g, OR 1.7 (95% CI 0.9–3.3) Subgroup analysis (nephrogenic rests) • >4,000 g OR 21.1 (95% CI 1.2–3.9)	2,500– <4,000 g	Matched for child's age, geographic area	Serious	Good	Fair
Heck et al. (2019), Denmark (73)	Case–control	217	• >4,000 g, OR 1.57 (95% CI 1.11–2.22) • LGA or 1.79 (95% CI 1.08–2.96)	2,500– <4,000 g	Matched for sex and year of birth	Low	Good	Fair
Heuch et al. (1996), Norway (71)	Cohort	199	Birth weight >4,000 g IRR 1.19 (96% CI 0.72–1.98)	3,001–3,500 g	Adjusted for age and sex	Moderate	Good	Fair
Jepsen et al. (2004), Denmark (74)	Case–control	126	Birth weight 4,000–4,499 g OR 0.88 (95% CI 0.44–1.62)	<3,500 g	No adjustments	Moderate	Good	Poor
Lindblad et al. (1992), Sweden (75)	Case–control	110	>4,000 g, OR 1.2 (95% CI 0.7–2.0)	<4,000 g	Matched or sex and date of birth	Moderate	Good	Poor
Olshan et al. (1993), USA (79)	Case–control	612	• Birth weight 4,001–4,500 g • AOR 1.27 (95% CI 0.65–2.51)	3,001–3,500 g	Adjusted for household income and father's education	Serous	Poor	Poor
O'Neill (2015), USA, UK (50)	Case–control	1,129, 1,515	• Birth weight per 0.5-kg increase • AOR 1.17 (95% CI 1.10–1.24) • AOR 1.12 (95% CI 1.05–1.18) • Birth weight ≥4,000 g • AOR 1.55 (95% CI 1.29–1.87) • AOR 1.31 (95% CI 0.98–1.77)	Per 0.5-kg increase, 3,000–3,490 g	Adjusted for maternal age, plurality, gender, state and year of birth, birth order, maternal ethnicity	Moderate	Good	Good
Puumala et al. (2008), USA (80)	Case–control	138	Birth weight >4,000 g AHR 1.54 (95% CI 0.99–2.40)		Adjusted for sex and year of birth	Moderate	Good	Fair
Rangel et al. (2010), Brazil (68)	Case–control	Eligible number of cases 544. Included number of cases 410	• Birth weight ≥4,000 g • OR 4.76 (2.72–8.28) g	<4,000 g	• Matched for gender and age • <50% responders among cases	Critical	Good	Poor
Schyz (90), Germany	Case–control	177	>4,000 g, OR 1.58 (95% CI 1.01–2.48)	2,500– <4,000 g	Stratified by gender, age and year of birth and adjusted for socioeconomy and degree of urbanization	Serious	Fair	Poor
Schyz (91), Denmark, Sweden, Finland, Norway	Case–control	690	• >4,500 g, OR 1.90 (95% CI 1.29–2.81) • LGA OR 1.76 (95% CI 1.21–2.57)	• 3,000–3,500 g • AGA	Matched by birth month and year, sex and country	Low	Good	Good
Smulevich et al. (1999), Russia (83)	Case–control	48	Birth weight >4,000 g OR 5.1 (95% CI 1.6–16.4)	2,500–4,000 g	No adjustments	Moderate	Fair	Poor
Yaezel et al. (1997), USA (66)	Case–control	169	Birth weight >4,000 g AOR 2.1 (95% CI 1.4–3.4)	<4,000 g	Adjusted for maternal age, birth order, gestational age, sex, maternal race, maternal/paternal education, income, age at diagnosis	Moderate	Good	Good

*OR, odds ratio; AOR, adjusted odds ratio; HR, hazard ratio; AHR, adjusted hazard ratio; SIR, standard incidence ratio; REOR, random-effects odds ratio; RR, relative risk; ARR, adjusted relative risk; IRR, incidence risk ratio; AIRR, adjusted incidence risk ratio*.

#### Breast Cancer

**Figure 2:**
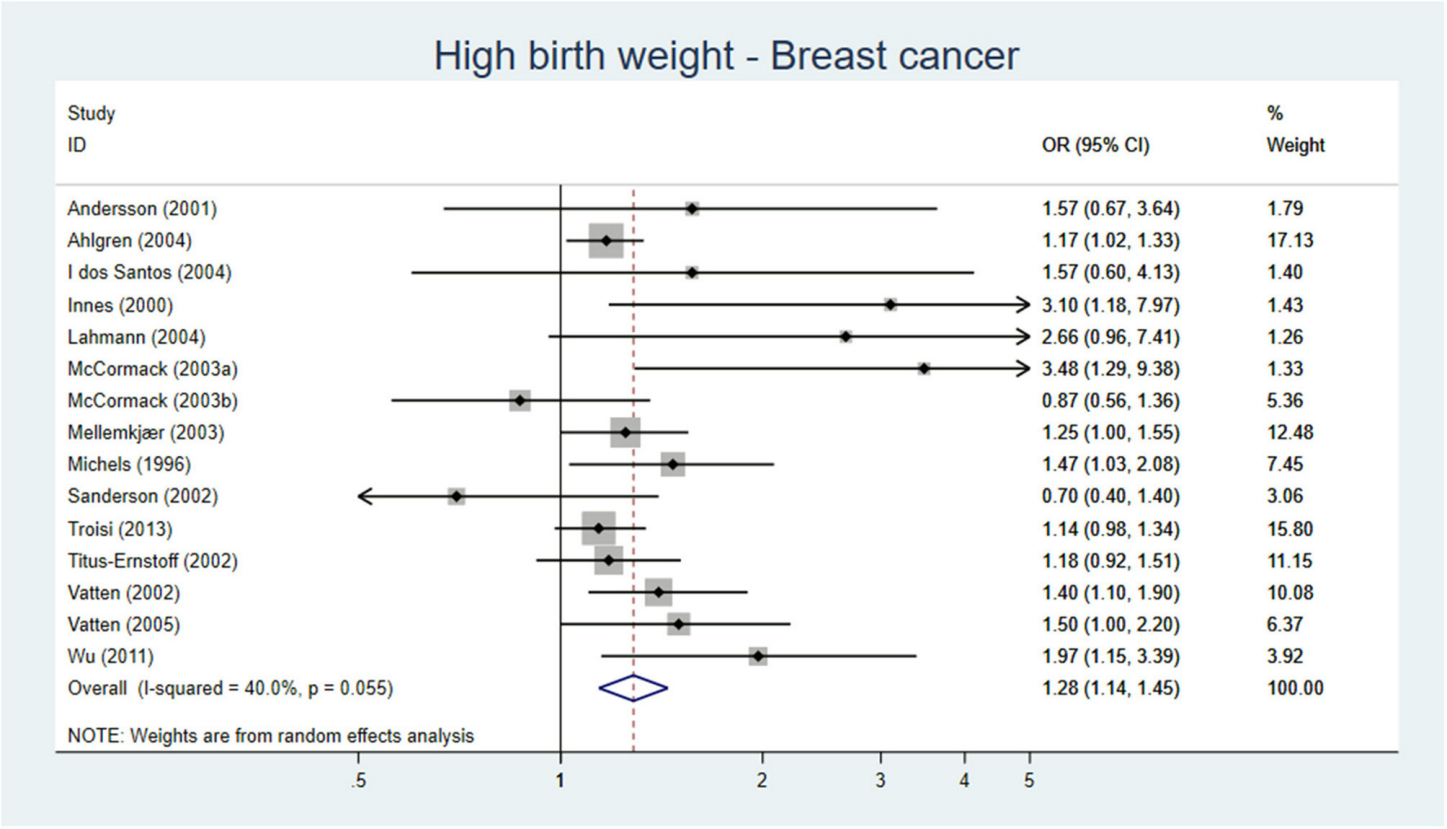
Forest plot describing the association between high birth weight and breast cancer.

Three SR/meta-analyses (23–25), 10 cohort studies (26–35), and nine case–control studies (14, 36–43) investigated the association between high birth weight and the risk of breast cancer. The three SR, one of high and two of low quality, reported an increase of breast cancer per 500 g increase in birth weight [RR 1.02 (95% CI 1.01–1.03)] (25) and if birth weight was >4,000 g [RR 1.23 (95% CI 1.13–1.24) and RR 1.15 (1.09–1.21)] (23, 24). Among the 10 cohort studies, five out of nine studies with low to moderate risk of bias (27–29, 31–35, 39), found an association between high birth weight and later development of breast cancer. Three out of four case–control studies with low to moderate risk of bias also found an association (37, 40, 42). When only evaluating studies with low risk of bias (32, 33, 40, 42), three studies found an association. Our meta-analysis including 15 original studies showed a pooled AOR of 1.24 (95% 1.11–1.39) for development of breast cancer, when comparing birth weight >4,000 or >4,500 g vs. birth weight of <4,000 g (Figure 2).

**Conclusion:** High birth weight is probably associated with a moderate increase in breast cancer, moderate certainty of evidence (GRADE ⊕⊕⊕O).

#### CNS Tumors

Four SR/meta-analyses, three cohort studies, 14 case–control studies, and one cross-sectional study reported on the association between high birth weight and CNS tumors. Two SRs, of medium and high quality, found an association between birth weight >4,000 g and astrocytoma [OR 1.38 (95% CI 1.07–1.79) and REOR 1.60 (95% CI 1.23–2.09)] and medulloblastoma [OR 1.27 (95% CI 1.02–1.60) and REOR 1.31(95% CI 1.08–1.58)] compared with <4,000 g (44, 45). A meta-analysis of medium quality (46) found for neuroblastoma, an OR of 1.19 (95% CI 1.04–1.36) for birth weight >4,000 g compared with <4,000 g. The SR/meta-analysis (high quality) by Georgakis and co-workers in 2017 (47) reporting on all CNS tumors, found an OR of 1.14 (95% CI 1.08–1.20) for high birth weight and an OR of 1.12 (95% CI 1.03–1.22) for LGA. Two cohort studies, both with low risk of bias, found an association between high birth weight and CNS tumors (48, 49), while one cohort study, with low risk of bias, found no association between LGA and CNS tumors (50). Nine out of 14 case–control studies had moderate risk of bias, where three studies (45, 51, 52) found an association between birth weight >4,000 g and CNS tumors, while six case–control studies, with moderate risk of bias, and one cross-sectional study (53) found no association.

**Figure 3:**
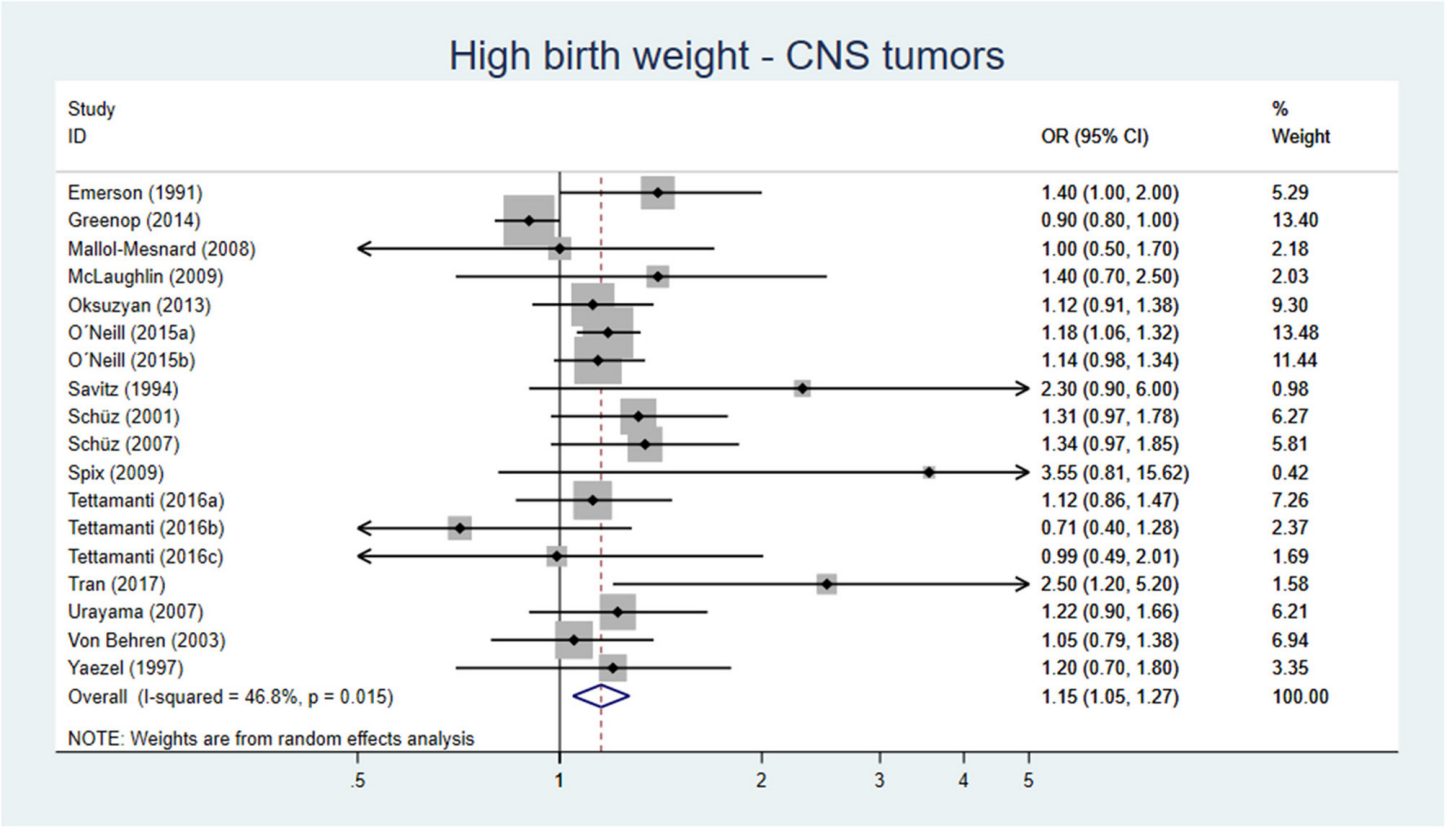
Forest plot describing the association between high birth weight and CNS tumors.

**Figure 4:**
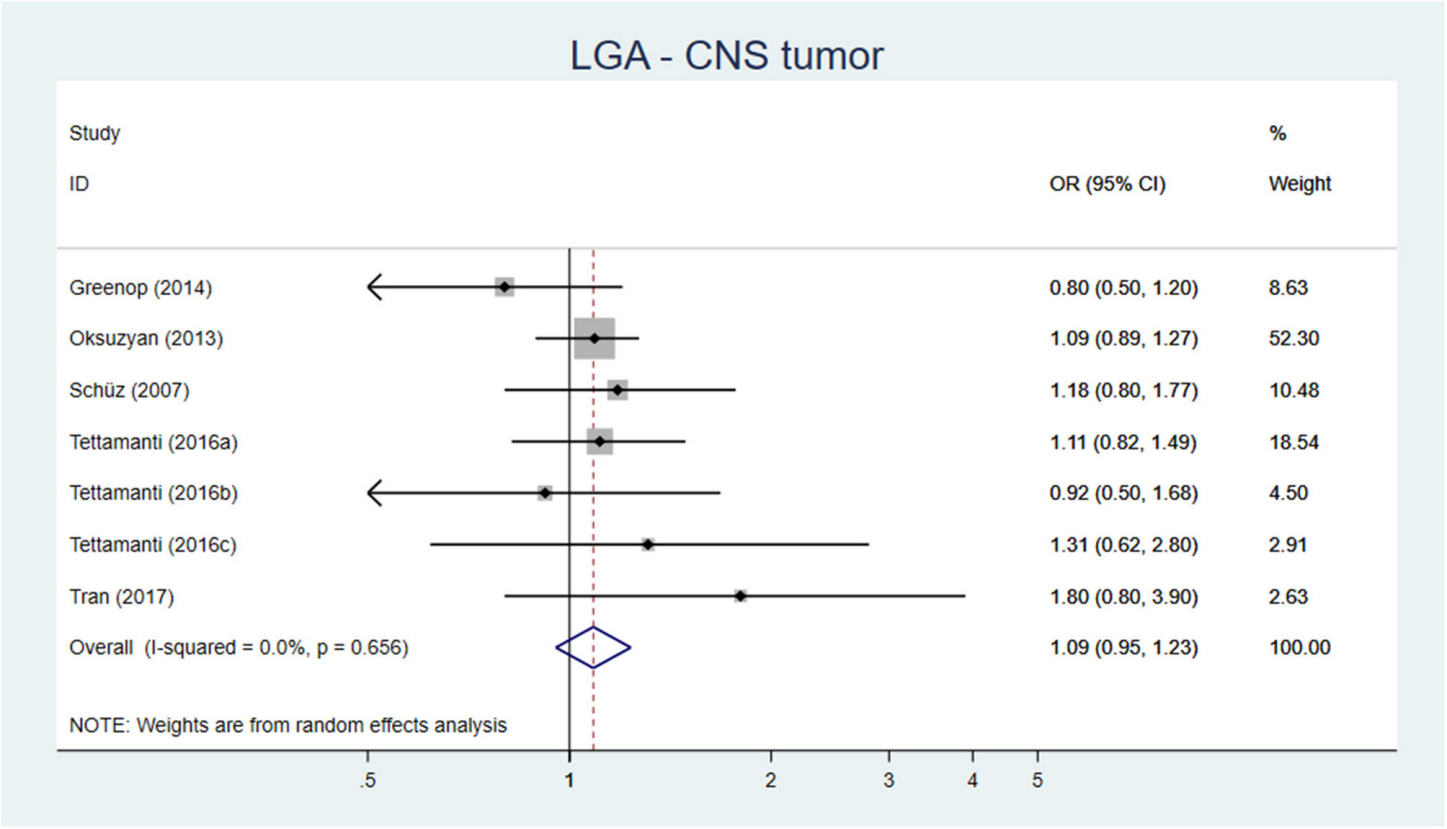
Forest plot describing the association between LGA and CNS tumor.

Our meta-analysis, including 15 original studies, showed a pooled AOR of 1.15 (95% CI 1.05–1.27) for development of CNS tumors, when comparing birth weight >4,000 or >4,500 g vs. birth weight of <4,000 g (Figure 3). For LGA vs. AGA, the corresponding figure was AOR 1.09 (95% CI 0.95–1.23) (Figure 4).

**Conclusion:** High birth weight is probably associated with a slight increase of CNS tumors, moderate certainty of evidence (GRADE ⊕⊕⊕O).

#### Hematological Malignancies

Two systematic reviews (54, 55), four cohort studies (34, 56–58) and 17 case–control studies (51, 52, 59–73) investigated the association between high birth weight and leukemia, one cohort study (74), and two case–control studies (16, 75) reported on lymphoma and five case–control studies (76–80) had investigated the impact of high birth weight on both leukemia and lymphoma.

##### Leukemia

**Figure 5:**
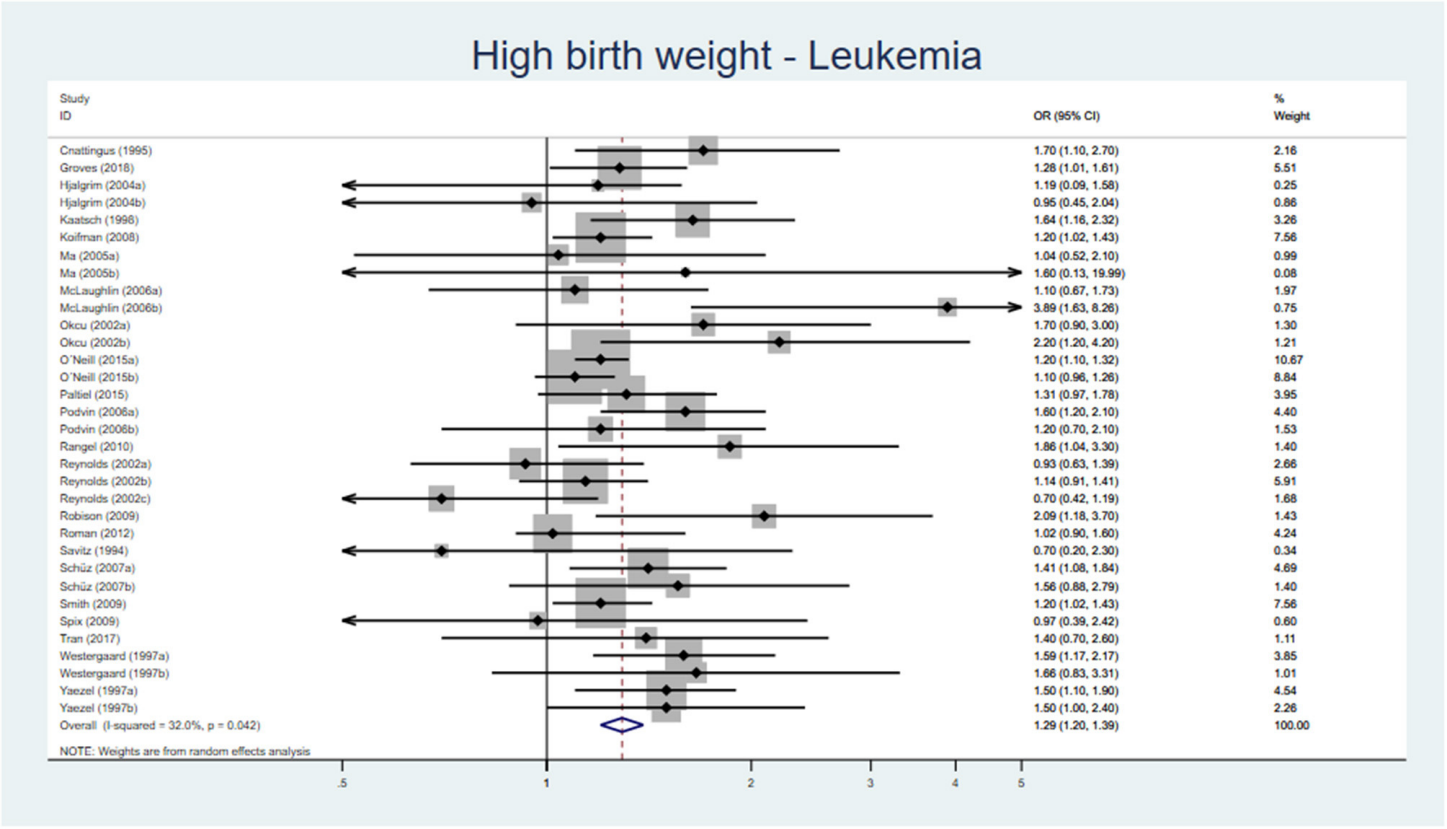
Forest plot describing the association between high birth weight and leukemia.

**Figure 6:**
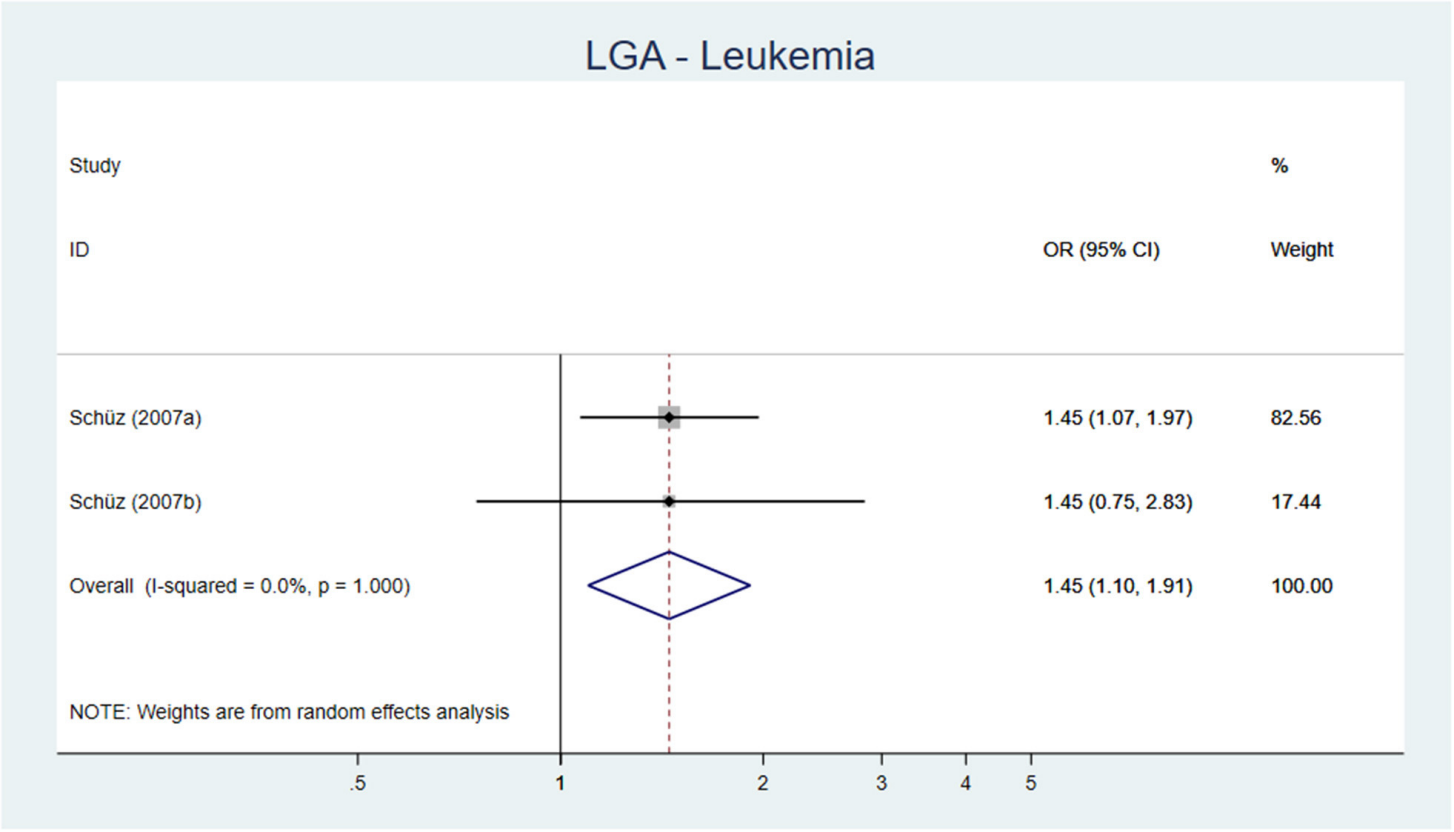
Forest plot describing the association between LGA and leukemia.

Both SR, of high and low quality, respectively, reported an association between birth weight >4,000 g and leukemia [OR 1.25 (95% CI 1.17–1.37) and AOR 1.35 (95% CI 1.24–1.48)] (54, 55). Two out of three cohort studies (56–58), all with low risk of bias, found an association between birth weight >4,000 g and acute lymphatic leukemia (ALL) (56, 58) and between LGA and ALL (56). Fourteen of the 22 case–control studies investigating the association between high birth weight and leukemia had a low to moderate risk of bias, and of these, 10 showed an increased risk if birth weight ≥4,000 or ≥4,500 g. The results from 22 original studies reporting on leukemia and high birth weight were pooled in a meta-analysis showing an AOR of 1.29 (95% CI 1.20–1.39) (Figure 5) and for LGA an AOR of 1.45 (95% CI 1.10–1.91) (Figure 6).

##### Lymphoma

One cohort and seven case–control studies reported on lymphoma. The cohort study by Petridou et al. (74) (low risk of bias) reported an increased risk for non-Hodgkin lymphoma when the child was born LGA while no significant increased risk was found for high birth weight. Two case–control studies with moderate risk of bias (16, 78), comparing >4,000 g as exposure to the reference <4,000 g, reported an association between high birth weight and Hodgkin/non-Hodgkin lymphoma. One case–control study, with moderate risk of bias reported an association between LGA and risk of Burkitt's lymphoma but no increased risk for other lymphomas (75).

**Conclusion:** High birth weight is probably associated with a moderate increase in leukemia, moderate certainty of evidence (GRADE ⊕⊕⊕O). LGA may be associated with a moderate increase in non-Hodgkin lymphoma, low certainty of evidence (GRADE ⊕⊕OO).

#### Wilm's Tumor

One SR (81), two cohort studies (82, 83), and 12 case–control studies (51, 78, 80, 84–92) reported on Wilm's tumor in childhood. The SR being of medium quality reported an increased risk for Wilm's tumor if birth weight >4,000 g as well as for LGA [OR 1.36 (95% CI 1.12–1.64) and OR 1.51 (95% CI 1.25–1.83)] (81).

**Figure 7:**
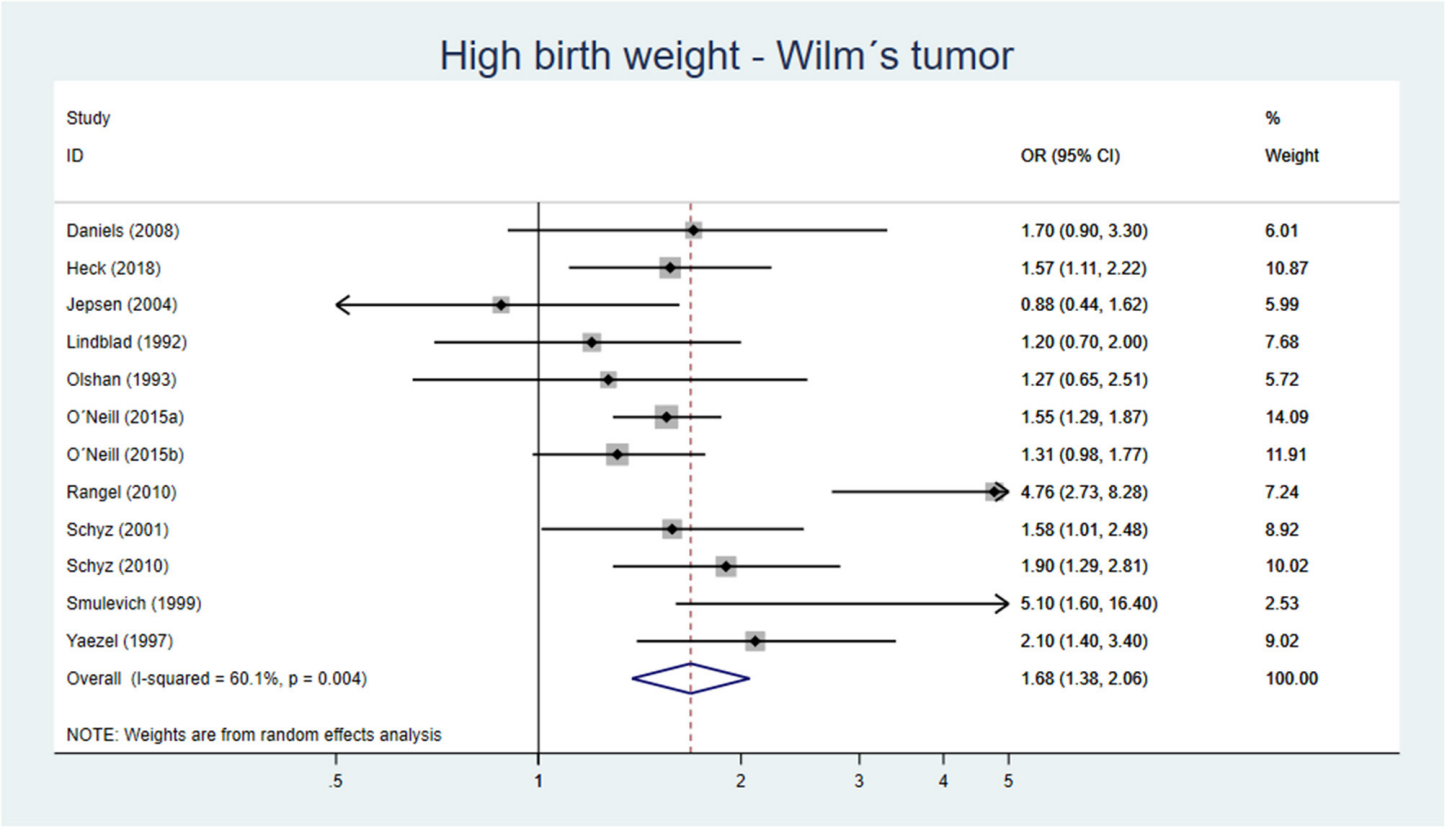
Forest plot describing the association between high birth weight and Wilm's tumor.

**Figure 8:**
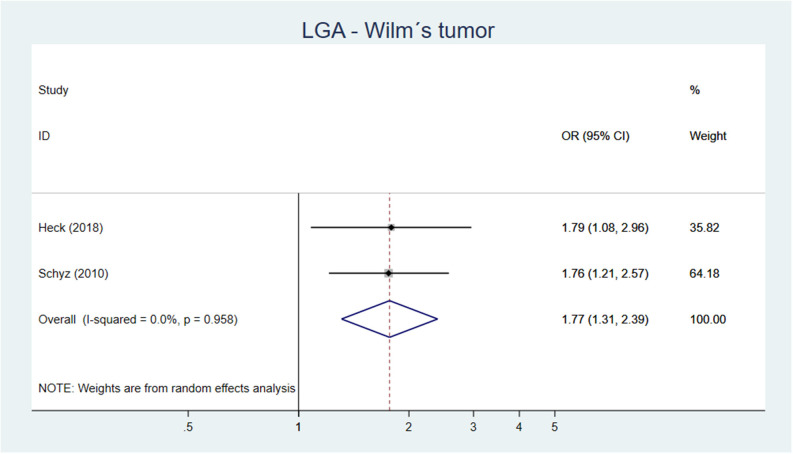
Forest plot describing the association between LGA and Wilm's tumor.

One out of two cohort studies with low-moderate risk of bias (82, 83) showed an association between high birth weight and Wilm's tumor (82). Five out of eight case–control studies, being of low to moderate risk of bias showed an increased risk of Wilm's tumor if birth weight >4,000 g or if LGA. Our meta-analysis including 11 original studies showed a pooled AOR of 1.68 (95% CI 1.38–2.06) for Wilm's tumor, when comparing birth weight >4,000 g vs. birth weight of <4,000 g (Figure 7). For LGA vs. AGA, the corresponding figure was AOR 1.77 (95% CI 1.31–2.39) (Figure 8).

**Conclusion:** High birth weight and/or LGA is probably associated with a moderate increase in Wilm's tumor, moderate certainty of evidence (GRADE ⊕⊕⊕O).

### Psychiatric Disorders

Outcomes are listed in Table 1.2a.

**Table 1.2a:** LGA, high birth weight, and long-term outcomes—psychiatric disorders.

**Author, year, country**	**Study design**	**Cases**	**Outcomes (risk estimates)**	**Reference group (weight)**	**Comments/adjustments**	**Risk of bias**	**Directness**	**Precision**
• **Psychiatric disorders** • **Systematic reviews** *n* = **1**								
Davies (100), UK	Systematic review, meta-analysis	Not reported	• Birth weight >4,000 g • OR 0.86 (95% CI 0.80–0.92)	Not stated	No adjustments performed			
• **Psychiatric disorders** • **Original articles** *n* = **10**								
Gunnell et al. (2003), Sweden (17)	Cohort 334,577	• 80 with schizophrenia • 124 with non-affective, non-schizophrenic psychosis	• Schizophrenia: • Birth weight >4,000 g • HR 3.37 (95% CI 1.68–6.74) • Non-affective psychosis: • HR 1.24 (95% CI 0.75–2.05)	3,501–4,000 g	Adjustments: gestational age, birth weight, birth length, ponderal index, head circumference, season of birth, urbanicity of residence at birth, age of mother, Apgar score at 1 minute, maternal parity, delivery by cesarean section, congenital malformation, uterine atony/prolonged labor, parental education	Moderate	Good	Good
Herva et al. (2008), Finland* (90)	• Cohort • 4,007 men and 4,332 women	1,026 (current), 315 (self-reported physician-diagnosed) depression	• Likelihood for current depression 4,500–4,999 g • men OR 1.21 (95% CI 0.72–2.03; women OR 2.02 (95% CI 1.20–3.39) • Likelihood for self-reported physician-diagnosed depression 4,500 g: men OR 1.30 (95% CI 0.50–3.40), women OR 0.46 (95% CI 0.11–1.90)	3,000–3,499 g	Adjustments: father's social class, mother's depression during pregnancy, mother's smoking during pregnancy, parity, mother's education, gestational age, mother's age at child's birth, mother's BMI before pregnancy	Moderate	Good	Good
Keskinen et al. (2013), Finland (87)	• Cohort • 10,526	150	• Schizophrenia • Birth weight >4,500 g • HR 2.0 (95% CI 1.0–4.0) • In the group without parental psychosis HR 1.5 (95% CI 0.7–3.4) • In the group with parental psychosis HR 11.4 (95% CI 3.3–39.7) • Birth weight >4,500 g in relation to gestational age and the risk of schizophrenia. HR 1.2 (95% CI 0.7–1.9), *p* = 0.46 • In the group without parental psychosis HR 1.0 (95% CI 0.6–1.7), *p* = 0.99 • In the group with parental psychosis HR 3.2 (95% CI 1.2–9.0), *p* = 0.03	2,500–4,500 g	The results were reported as gender-adjusted HRs with 95% CIs. The association between parental gender, gestational age, psychosis, and birth weight was adjusted for maternal BMI (continuous variable)	Low	Good	Good
Lahti et al. (2015), Finland (92)	Cohort 12,597	1,660	• Risk of any mental disorder (all subjects) LGA HR 1.03 (95% CI 0.75–1.41) • Risk of psychotic disorder (women) LGA HR 2.43 (95% CI 1.19–4.96)	AGA = between −2 and +2 SD of that predicted by gestational age	Stratified for sex and year of birth, and adjusted for gestational age, socioeconomic position in childhood and mothers' marital status at childbirth	Low	Good	Good
Liuhanen et al. (2018), Finland (88)	• Cohort 4,223, • Family study • 256	256	• Schizophrenia: Birth weight >4,000 g and high genetic risk OR 2.7 (95% CI 1.2–6.0) *p* = 0.013 • For women OR 7.6 (95% CI 2.8–20.5) • In fully adjusted model, there was no interaction between birth weight and genetic risk of social anhedonia (*p* = 0.61), or schizophrenia diagnosis (*p* = 0.24)	Those with low genetic risk and birth weight ≤4,000 g	Adjustments: sex, gestational age, mother's BMI, and 3 principal component analyses	Low	Good	Fair
Moilanen et al. (2010), Finland (84)	Cohort 10,934	111	• Risk of schizophrenia: Birth weight ≥4,500 g OR 2.4 (95% CI 1.1–4.9) • Large babies (>2 SD) for “corrected” gestational age • OR 2.1 (95% CI 1.0–5.1)	2,500–4,499 g	Adjusted for gestational age, parental history of psychosis, sex	Low	Good	Fair
Perquier et al. (2014), France (89)	Cohort 41,144	2,601 with new onset, 3,734 with recurrent depression	• Risk of depression • Birth weight >4,000 g • New-onset OR 1.16 (95% CI 1.01–1.34), Recurrent OR 1.11 (95% CI 0.99–1.26)	2,500–4,000 g	Adjustments: age; time since menopause; age at menarche; physical activity; energy intake; marital status; educational level; World War II food deprivation; psychological difficulties at work; alcohol intake; tobacco status; menstrual cycle length; number of children; type of menopause; history of cancer, type 2 diabetes, or vascular diseases; sleep duration; menopausal hormone therapy use	Low	Good	Good
Van Lieshout et al. (2020), Canada (93)	• Cohort • 2,151	628	• Birth weight >4,000 g • Conduct disorder, OR 3.19 (95% CI 1.37–7.43) • Oppositional defiant disorder (ODD), OR 1.79 (95% CI 1.11–2.91), • ADHD OR 1.77 (95% CI 1.21–2.80) • Birth weight >4,000 g and socioeconomic disadvantage • ODD OR 5.86 (95% CI 2.60–13.25) • Major depressive disorder • OR 4.24 (95% CI 1.69–10.66), Generalized anxiety disorder OR 3.85 (95% CI 1.64–9.08) compared with those with higher socioeconomic status	2,500–4,000 g	Adjusted for participant age, sex, socioeconomic status of the family, parental mental health, and gestational DM	Moderate	Fair	Good
Wegelius et al. (2011), Finland (85)	• Cohort • 1,051	360	• Schizophrenia • Birth weight >4,000 g • HRR 1.68 (95% CI 1.13–2.50), *p* = 0.010 • Risk of primary psychotic disorder • Birth weight >4,000 g • HRR 1.18 (95% CI 0.84–1.65), *p* = 0.35	3,000–4,000 g	Adjustments: sex, maternal and paternal history of psychotic disorder	Moderate	Good	Fair
Wegelius et al. (2013), Finland (86)	Cohort 1,051	282	High birth weight (>4,000 g) was associated with more severe symptoms of bizarre behavior, as reflected by the statistically significant quadratic term (β_Linear_ = −3.92, SE = 0.76, *p* < 0.001; β_Quadratic_ = 0.57, SE = 0.12, *p* < 0.001)	3,000–4,000 g	Adjusted for sex, place of birth and year of birth	Moderate	Good	Fair

*ADHD, attention deficit hyperactivity disorder; AGA, appropriate for gestational age; BMI, body mass index; CI, confidence interval; HR, hazard ratio; HRR, hazard rate ratio; LGA, large for gestational age; NA, not available; ODD, oppositional defiant disorder; OR, odds ratio*.

#### Schizophrenia

Four out of six cohort studies, with low to moderate risk of bias, found an association between high birth weight and/or LGA and schizophrenia (17, 93–95). All studies but one (17) included both males and females and were adjusted by sex. High birth weight also increased the risk of schizophrenia considerably in families with parental psychosis (94, 96). However, two studies found no association in adjusted models (96, 97).

#### Depression

Two cohort studies, one with low and one with moderate risk of bias reported on depression. In these studies, women born with high birth weight had increased risk for new-onset depression (98) and current depression (98, 99). In men, no association was found (99).

#### Psychiatric Disorders in General

According to a recent systematic review and meta-analysis, high birth weight >4,000 g was a protective factor for different types of psychotic disorders (OR 0.86, 95% CI 0.80–0.92) (100). In our search, we found three cohort studies investigating the association between several mental or psychotic disorders and high birth weight with contradictory results. According to two Finnish studies, no general increased risk of any mental disorder (substance use, psychotic, mood, anxiety, personality disorders, suicides, suicide attempts) or any primary psychotic disorder was observed in individuals born LGA (95, 101). However, Van Lieshout et al. (102) reported higher odds of some psychiatric disorders [oppositional defiant disorder, conduct disorder, attention deficit hyperactivity disorder (ADHD)] in 12–17-year-old children born macrosomic (102). Participants exposed to macrosomia and socioeconomic disadvantage were more susceptible to major depressive disorders, and generalized anxiety disorders, compared with those with higher socioeconomic status (102).

**Conclusion:** High birth weight and/or LGA may be associated with a moderate increase in schizophrenia and an increase in depression, low certainty of evidence (GRADE ⊕⊕OO).

It is uncertain whether high birth weight is associated with psychiatric disorders in general, very low certainty of evidence (GRADE ⊕OOO).

### Cognitive Function

Outcomes are listed in Table 1.2b.

**Table 1.2b:** LGA, high birth weight, and long-term outcomes—cognitive performance.

**Author, year, country**	**Study design**	**Cases**	**Outcomes (risk estimates)**	**Reference group (weight)**	**Comments/adjustments**	**Risk of bias**	**Directness**	**Precision**
**Original articles** ***n*** **=** **21**								
Alati et al. (2009), Australia (98)	• Cohort • 4,971		• Social problems Quintile 5 (highest birth weight): OR 1.57 (95% CI 1.12–2.20) • Anxious/depressive symptoms Quintile 5: OR 1.1 (95% CI 0.80–1.51)	Quintile 3	Adjustments: parity and child age, socio-economic position, maternal alcohol and tobacco use, maternal anxiety and depression in pregnancy	Moderate	Good	Good
Bergvall et al. (2006), Sweden (108)	• Cohort • 357,768	35,821	Risk of low intellectual performance: birth weight (SDS) more than 2: OR 0.98 (95% CI 0.90–1.06)	Birth weight (SDS) −2 to +2	Adjustments: gestational age, mothers age and parity, socioeconomic factors (household socioeconomic status, education, family structure)	Moderate	Good	Good
Buschgens (2009), The Netherlands (97)	• Cohort • 2,230		• Birth weight >4,500 g • Inattention (TCP^**^p < 0.01); • Hyperactivity/impulsivity (TCP *p* < 0.01) • Aggression (CBCL^***^ <0.05; TCP < 0.01) • Delinquency (TCP < 0.01)	2,500–4,500 g	Multiple linear regression analyses, for each separate (standardized) variable	Low	Good	Good
Dawes et al. (2015), UK (114)	• UK Biobank resource • 18,819		For hearing, vision, reaction time and IQ, the middle category had significantly better performance than both the low and high categories (both *p* < 0.001)	The top and bottom 3% by birth weight were compared with the middle 3% (centered on the 50th percentile)	An ANOVA model was applied, hearing, vision, and cognition as the dependent variable and group (bottom, middle, or top 3% of the distribution) as the independent variable in the model, with the covariates age, sex, Townsend deprivation index quintile, educational level, smoking, diabetes, cardiovascular disease, hypertension, high cholesterol, and maternal smoking	Serious	Poor	Fair
Duffy et al. (2020), USA (113)	• Cohort • 108,348		• Children born LGA • Did not meet proficiency on mathematics ARR 0.96 (95% CI 0.92–0.99) • Did not meet proficiency on English language or arts ARR 0.97 (95% CI 0.95–0.99) • Referred for special education ARR 0.98 (95% CI 0.94–1.03)	AGA	Adjustments: maternal ethnicity, age, education, nativity, marital status, Medicaid status, parity, maternal obesity, pre-gestational or gestational diabetes, tobacco, alcohol, or drug during pregnancy, excessive weight gain during pregnancy, infant gender, and year of birth	Moderate	Good	Good
Eide et al. (2007), Norway (109)	Cohort 317,761	4,912	Large infants (z-score birth weight >3.00) had a slightly elevated risk of low intelligence score (OR 1.22, 95% CI 1.00–1.48)	*z*-score −0.49 to 0.50	Adjustments: maternal age, maternal education, parity, adult height, BMI The gestational age–specific z-score (SD above or below the mean of birth weight was calculated using Norwegian population standards)	Moderate	Good	Good
Flensborg-Madsen and Mortensen (2017), Denmark (112)	Cohort 4,696		• Standardized intelligence score • Birth weight >4,000 g • At the age 19 years • mean difference 1.35 (95% CI −0.83 to 3.52), 28 years −0.03 (−4.05 to 4.00), 50 years 2.90 (−0.35 to 6.14)	3,001–3,500 g	Adjustments: infant sex, infant socioeconomic status, mother's age at birth, birth order, mother's smoking in last trimester, gestational age	Moderate	Good	Good
Haglund and Källen (2011), Sweden (94)	• Case–control • 68,964	250	• Both autism and Asperger: LGA vs. adequate weight for gestational age OR 0.3 (95% CI 0–1.9) • Any obstetrical risk factor (prematurity, low Apgar scores, growth restriction, or macrosomia) • Autism with mental retardation, AOR 1.3 (95% CI 0.3–2.2) • Autism without cognitive impairment AOR 3.1 (95% CI 1.7–5.7)	2,500–4,000 g	Adjusted for year of birth, maternal age 40 years or older, primiparity, maternal birth outside Sweden, and gender	Moderate	Fair	Good
Kristensen et al. (2014), Norway (111)	• Cohort • 217,746		• The crude mean IQ score • Birth weights of ≥5,000 g was 1.2 points (95% CI 0.3–2.2) lower	4,000–4,499 g	In the multivariable analysis included gestational age, year of birth, birth order, sibship size, mother's and father's ages at child's birth, mother's marital status, highest parental educational level, father's income level. Mean sibship birth weight, maximum sibship birth weight, and fraternal relatedness were added to the random-effects model	Moderate	Good	Good
Leonard et al. (2008), Australia (95)	Cohort 219,877	2,625	• Mild-moderate ID (>4,500 g) OR 1.10 (95% CI 0.75–1.61) • Severe ID: OR 1.29 (95% CI 0.40–4.10); ID with autism spectrum disorder: OR 1.66 (95% CI 0.60–4.56) • Caucasian infants with excess intrauterine growth (percentage of optimal birth weight 124) were more likely to be diagnosed with ID associated with autism spectrum disorder OR 2.36 (95% CI 0.93–6.03)	3,000–3,499 g	Adjustments: marital status, maternal country at birth, health insurance status, paternal occupation, geographic remoteness, socioeconomic well-being	Moderate	Good	Good
Lundgren et al. (2003), Sweden (110)	Cohort 620,834		• Risk for subnormal intellectual performance: • High birth weight (>2 SDS) according to the BMI groups at young adulthood: normal BMI (18.5–24.9) OR 0.92 (95% CI 0.87–0.98), BMI 25–29.9 OR 1.33 (95% CI 1.20–1.48), BMI >30 OR 1.86 (1.58–2.19)	Subjects born at term with normal birth weight	Adjusted for gestational age, low Apgar score, head circumference SDS at birth, height SDS at conscription and parental education	Moderate	Good	Good
Moore et al. (2012), USA (96)	Cohort 5,979,605	20,206	• Risk of autism: • Term LGA (95th percentile) infants 39–41 weeks AOR, 1.16 (95% CI 1.08–1.26) Preterm LGA infants 23–31 weeks AOR, 0.45 (95% CI 0.21–0.95)	Subjects born with birth weight AGA	Adjusted for maternal age, race, hypertension, pre-eclampsia, diabetes, birth order, twin gestation, and months since last live birth	Moderate	Good	Good
Power et al. (2006), UK (107)	• Cohort • 13,980		• For 1 kg increase in birth weight, 7-year mathematics *z*-score increased 0.23 (0.19 adjusted for parental interest in child's progress) and adult qualifications increased 0.22 (on a 5-point scale) • Mean *z*-scores for math (>4,000): • boys 0.10, girls 0.14		Adjustments for gender, gestational age (32–44 weeks), exact age of test and for parental interest in child's progress	Moderate	Good	Good
Record et al. (1969), UK (103)	Cohort 41,543		• Mean verbal reasoning scores of first-born children (40–41 weeks of gestation) • Birth weight 2,000–2,400: 96.9–98.9 • Birth weight 3,000–3,400: 102.1–104.2 • Birth weight 4,000–4,400: 104.3–105.3		Results reported according to sex, duration of gestation, birth order	Moderate	Poor	Good
Richards et al. (2001), UK (105)	Cohort 3,900		• Birth weight was associated with cognitive ability at age 8 (with an estimated SD score of 0.44 (95% CI 0.28–0.59)) between the lowest and highest birth weight categories • At age 43 high birth weight (4,010–5,000) vs. normal birth weight • Standardized cognitive score: • Verbal memory −0.17 (−0.31 to −0.04) • Search accuracy 0.02 (−0.11 to 0.16) • Search speed −0.07 (−0.21 to 0.07)	3,010–3,500 g	Adjusted for sex, father's social class, mother's education, birth order, and mother's age. From age 11 to age 43, each cognitive score was further adjusted for the score of previous age	Moderate	Good	Good
Räikkonen et al. (2013), Finland (106)	Cohort 931	The whole cohort	Men who were born larger were more likely to perform better in the Finnish Defense Forces Basic Intellectual Ability Test over time [1.22–1.43 increase in odds to remain in the top relative to the lower two thirds in ability over time per each SD increase in body size (95% CI 1.04–1.79)]		• No specific mention of birth weight categories • Adjustments: gestational age, mother's age, height and parity; social class in childhood; history of breast feeding; education; diagnosis of diseases	Low	Good	Good
Sörensen et al. (1997), Denmark (104)	• Cohort • 4,300		• The Boerge Piren test (validated intelligence test) increased from 39.9 at a birth weight of ≥2,500 g to 44.6 at a birth weight of 4,200 g. • Above a birth weight of 4,200 g the test score decreased slightly		Adjusted for gestational age, length at birth, maternal age and parity, marital status, and employment	Moderate	Good	Good
Tamai (2020), Japan (101)	Cohort 36,321		• At 2.5 years: • Unable to walk ARR 7.1 (95% CI 1.0–5.9) • Unable to say meaningful words ARR 10 (95% CI 3.8–26) • Unable to compose two-phrase sentence ARR 3.5 (95% CI 1.9–6.3) • Unable to say his/her name ARR 1.9 (95% CI 1.2–3) • Unable to use a spoon ARR 4.8 (95% CI 1.9–12.3) • All differences disappeared at 5.5 years of age • However not for LGA >3 SD	• −1.28 to 1.28 SD • Normal birth weight	Adjustments: parity, singleton, gender, maternal age, maternal smoking, maternal and paternal education level	Moderate	Good	Fair
van Mil et al. (2015), The Netherlands (100)	• Cohort • 6,015		• Risk of attention problems in children born with high birth weight percentile β (95% CI): • The attention problems subscale of the CBCL/1.5–5^***^ • >90th percentile 0.05 (−0.02 to 0.12) p value 0.17 • >80th percentile 0.01 (−0.07 to 0.04), *p* = 0.61	Subjects born with birth weight AGA	Adjusted for Apgar score 1 minute after birth, mode of delivery, maternal age, national origin, educational level, parity, BMI, psychological symptoms, smoking, alcohol use, folic acid supplementation use, gestational diabetes, pre-eclampsia	Moderate	Good	Good
Yang et al. (2019), China (99)	Cohort 9,295	724	• Behavioral problems • Macrosomia (*n* = 268) OR 1.61 (95% CI 1.16–2.22)	Normal and low birth weight	Adjustments: age, sex	Serious	Poor	Good
Zhang et al. (2020), China (102)	Cohort	4,026	• Gross motor DQ ARC 0.49 (95% CI 0.36–0.63) • Fine motor DQ ARC −2.73 (95% CI −2.87 to −2.59) • Adaptability DQ ARC −1.19 (95% CI −1.33 to −1.05) • Language DQ ARC 0.43 (95% CI 0.29–0.57) • Social behavior DQ ARC 1.10 (95% CI 0.95–1.24) • Overall no clear differences	Normal birth weight	Adjustments: maternal smoking, gender of infant, mode of delivery, neonatal asphyxia, birth length, gestational week, educational level of parent	Moderate	Fair	Fair

** *Teacher's Checklist of Psychopathology*.

*** *Child Behavior Checklist*.

*AGA, appropriate for gestational age; AOR, adjusted odds ratio; ARC, adjusted regression coefficient; ARR, adjusted relative risk; BMI, body mass index; CBCL, The Child Behavior Checklist; DQ, development quotient; ID, intellectual disability; IQ, intelligence quotient; LGA, large for gestational age; NA, not available; OR, odds ratio; SDS, standard deviation score; TCP, The Teacher's Checklist of Psychopathology*.

#### Autism

One case–control study with moderate risk of bias reported no association of LGA with autism or Asperger syndrome (103). Two cohort studies with moderate risk of bias reported a slightly increased risk for autism in children born LGA (104, 105).

#### Behavioral Problems

Four cohort studies reported results on associations between high birth weight/LGA and behavior/attention problems among children and adolescents aged 6–16 years, of which three reported an association between LGA and behavioral problems (106–108).

In a study with low risk of bias, a higher risk for externalizing behaviors (inattention, hyperactivity/impulsivity, aggression, delinquency) was found in high birthweight children (106). In another study with moderate risk of bias, an association between birth weight and social problems was observed in babies at the higher end of the birth weight distribution (107). In contrast, one study (109) found that high birthweight children had no increased risk of attention problems. In a study from Japan, the relation between LGA and neurodevelopment was U-shaped, with mild LGA having the lowest risk and severe LGA (>3 SD) was associated with higher risk of unfavorable behavioral development (110), while another study found no association (111).

#### Cognitive Development

In five cohort studies with low or moderate risk of bias, high birth weight was associated with high cognitive ability (112–115) and 7-year math score (116).

#### Intellectual Performance

Eight cohort studies investigated the association between high birth weight and intellectual performance, seven with moderate and one with serious risk of bias. Five of these studies consisted of a study population of Nordic conscripts (117–121), one was a large cohort study of children born in Western Australia (104) and one study was from the USA (122). In five studies, no clear association was found between high birth weight and intellectual performance, risk of intellectual disability, or low IQ score (104, 117–119, 121). However, in one study the crude mean IQ score was 1.2 points lower for those with the extreme birth weight (≥5,000 g) (120). The major part of the apparent association between high birth weight and low IQ score was caused by confounding family factors (120). Of note, the risk for subnormal intellectual performance was dependent on a BMI at young adulthood BMI >30 OR 1.86 (1.58–2.19) (119). In the recently published study from the USA, a slightly decreased risk of poor academic performance was noticed for LGA children (122). In addition, one study from UK Biobank, the middle birth weight category showed better performance for hearing, vision, reaction time, and IQ than the highest category (123).

**Conclusion:** High birth weight and/or LGA may be associated with a slight increase in autism and behavioral problems, low certainty of evidence (GRADE ⊕⊕OO). High birth weight may be positively associated with cognitive ability, low certainty of evidence (GRADE ⊕⊕OO). No association was found between high birth weight and/or LGA and intellectual performance, moderate certainty of evidence (GRADE ⊕⊕⊕O).

### Cardiovascular Health

Outcomes are listed in Table 1.3. Two SR/meta-analyses of high quality, one on hypertension and blood pressure (19) and one on coronary heart disease (CHD) (124), were included, together with 27 original articles.

**Table 1.3:** LGA and high birth weight and long-term outcomes—cardio-vascular diseases.

**Author, year, country**	**Study design**	**Cases**	**Outcomes (risk estimates)**	**Reference group (weight)**	**Comments/adjustments**	**Risk of bias**	**Directness**	**Precision**
**Cardio-vascular**								
**Systematic review/meta-analysis**, ***n*** **=** **2**								
Zhang et al. (2013), China (19)	• SR meta-analysis • 31 studies	NA for hypertension	• Overall weighted mean differences (WMD) (all age groups) • SBP: −0.25 mmHg (95% CI −0.92 to 0.42) • DBP: 0.20 mmHg (95% CI −0.23 to 0.62) • Hypertension: • RR: 1.00 (95% CI 0.93–1.06) • SBP, DBP, and risk of hypertension are higher among individuals with HBW during childhood but lower during adulthood	• NBW 2,500–4,000 g or the 10–90th percentile for GA • NBW *n* = 559,979	Not specified			
Wang et al. (2014), China (115)	SR+ Meta-analysis	• Cases with CHD: • *n* = 11,218 • –	• CHD in HBW vs. NBW • Pooled OR (random-effects model) • OR 0.89 (95% CI 0.79–1.01)	NBW 2,500–4,000 g	Non-Adjusted			
CVD, Original articles, *n* = 21								
Blood pressure/hypertension, *n* = 14								
Azadbakht et al. (2014), Iran (116)	• Cohort • *n* = 5,528 • *n* = 2,726 girls • *n* = 2,802 boys		• HBW • High SBP AOR 0.6 (95% CI 0.3–1.2) • High DBP AOR 0.8 (95% CI 0.4–1.6)	2,500–4,000 g	Adjustments: Age, sex, SES, parent's income, parent's education, birth order, family history of chronic disease, breast feeding during, type complementary food, sedentary lifestyle, BMI	Serious	Fair	Fair
Dong (2017), China (117)	• Cross sectional • High birth weight *n* = 4,981 • Normal birth weight *n* = 4,981	• High blood pressure • Boys *n* = 2,144 • Girls n = 1,086	• High blood pressure • Boys: AOR 0.96 (95% CI 0.77–1.20) • Girls: AOR 0.91 (95% CI 0.68–1.22)	2,500–3,999 g	• Matched age, sex, province • Adjusted: Parental education, delivery, breast feeding, family history of disease, food intake and physical activities, BMI	Serious	Poor	Good
Espineira (2011), Brazil (118)	• Cohort • *n* = 515	Continuous outcome	LGA had higher BP than controls (*p* < 0.05)	AGA	• Gender matched • Adjusted: Gender, waist circumference and height	Serious	Fair	Poor
Ferreira (2018), Brazil (119)	• Cross-sectional • School based • *n* = 829	• High BP • *OBP 8.5% *n* = 70 • **HoBP 3.8% • *n* = 32	Each increase of 100 g in birth weight did not influence office or home BP	BW	Simple linear regression analysis	Serious	Fair	Fair
Gunnarsdottir et al. (2002), Iceland (120)	• Cohort • *n* = 4,601 total • *n* = 2,337 men • *n* = 2,264 women	• Hypertension • 40–47% of women • 59–61% of men • Numbers NA	• Risk for hypertension • Women, AOR (95% CI): • ≤ 3.45 kg • 1.4 (95% CI 1.1–1.8) • >3.45 to ≤3.75 kg • (95% CI 0.8–1.3) • >4.0 kg • 0.9 (95% CI 0.7–1.2) • P for trend* <0.001 • P for trend** <0.001 • Men, AOR (95% CI): • ≤ 3.45 kg • (95% CI 0.8–1.3) • >3.45 to ≤3.75 kg • (95% CI 0.8–1.2) • >4.0 kg • 0.8 (95% CI 0.7–1.1) • *P* for trend* <0.051 • *P* for trend** <0.004 • Inverse association between size at birth and adult hypertension, strongest among women born small who were overweight in adulthood and for those without a family history of hypertension	3,750–4,000 g	• Adjusted for adult BMI, education, smoking habits, physical activity or family history of hypertension • Adjusted for trend: • *age, year of birth • ** age, year of birth, BMI	Moderate	Good	Good
Kuciene et al. (2018), Lithuania (121)	• Cross-sectional • Singleton, adolescents *n* = 4,598 • Boys *n* = 2,103 • Girls *n* = 2,495	• High blood pressure • *n* = 1,178	• Risk for high blood pressure • >4,000 g AOR 1.34 (95% CI 1.11–1.63)* • LGA AOR 1.44 (95% CI 1.16–1.79)* • >4,000 g and normal weight in adolescence: • AOR 1.37 (95% CI 1.11–1.70)** • 2,500–4,000 g and overweight/obesity • AOR 3.63 (95% CI 2.99–4.41)** • >4,000 g and overweight/obesity • AOR 4.36 (95% CI 3.04–6.26)** • LGA and normal weight in adolescence: • AOR 1.40 (95% CI 1.10–1.80)** • AGA and overweight/obesity • AOR 3.39 (95% I 2.79–4.13)** • LGA and overweight/obesity • AOR 5.03(95% CI 3.33–7.60)**	• 2,500–4,000 g • AGA	• *Adjustments in multivariable logistic regression analysis: • age, sex, and BMI • ** Adjustments in multivariable logistic regression analysis: • age and sex	Moderate	Good	Fair
Launer et al. (1993), Netherlands (122)	• Cohort • *n* = 374	Continuous outcome	Relation between SBP and birth weight appeared U-shaped in 4-year-old children	Birth weight	Adjusted for sex, gestational age, birth length, BP at 1 week (mmHg), blood pressure at 3 months (mmHg), current weight (kg)	Serious	Fair	Poor
Ledo et al. (2018), Brazil (123)	• Cross-sectional • *n* = 719	• SBP >90th • percentile • *n* = 22 • DBP >90th • Percentile • *n* = 36	HBW was not associated with high blood pressure	2,500–3,999 g	Adjusted for sex	Moderate	Fair	Poor
Li et al. (2006), USA (124)	• Longitudinal cohort • *n* = 98	• NA • Continuous outcome	• Birth weight was inversely associated with SBP in children in pre-pubertal stage but was not statistically significant in early or late puberty (*r* = −0.23 (SD 1.1), *p* < 0.05) • SBP significantly increased from pre-puberty to early or late puberty (sexual maturation) among children with HBW	<4,000 g	Adjusted for gender, race, age, pubertal status, BMI percentile	Serious	Poor	Fair
Li et al. (2013), China (125)	• Cohort • Childhood • *n* = 1,415 • Adolescence *n* = 1,112	Continuous outcome	• Childhood SBP and DBP: • No statistically significant difference • Adolescence • SBP • Cases: 110.83 ± 9.43 mmHg • Controls: 109.33 ± 9.26 mmHg • *P* = 0.0002 • DBP • Cases:72.10 ± 6.39 mmHg • Controls: 71.58 ± 6.47 mmHg • *P* = 0.055 • Similar results after adjustment in multi-mixed model	2,500–4,000 g	• Controls matched by sex and birth date • Adjustment in multi-linear analysis: • Repeated measures, birth year, sex, mother's occupation, age of delivery and adding weight during pregnancy, hypertension during delivery, gestational age, parity, and picky eating in childhood	Moderate	Fair	Fair
Schooling et al. (2010), China (126)	• Longitudinal cohort study • Men *n* = 5,051 • Women • *n* = 13,907	• High blood pressure • Men 55.9% (*n* = 2,824) • Women 47.2% (*n* = 6,564)	• Risk of HBP • per birth weight SD: • All: AOR 0.94 (95% CI 0.91–0.97)	Birth weight	Adjusted for study phase, age and sex, SES, number of offspring, height, BMI, WHR	Serious	Poor	Good
Strufaldi et al. (2009), Brazil (127)	• Cross-sectional • *n* = 739	Continuous outcome	• Inverse association between birth weight and BP • SBP and DBP was negatively associated with BW • Adjusted SBP: • Q1: 105.3 (95% CI 103–107.5) • Q2: 94.8 (95% CI 92.7–96.9) • Q3: 95.5 (95% CI 93.4–97.6) • Q4: 95.7 (95% CI 93.6–97.8)	• BW quartiles. • Q1: ≤2.9 kg • Q2: 2.91–3.20 kg • Q3: 3.21–3.58 kg • Q4: >3.58 kg	Adjusted for gender, prematurity, BMI	Serious	Fair	Fair
Tan et al. (2018), China (128)	• Cohort • *n* = 49,357	• High SBP • *n* = 7,654High DBP • *n* = 4,787Hypertension • *n* = 9,479	• High birth weight • Adjusted OR of hypertension • AOR 0.84 (95% CI 0.77–0.92) • High SBP • AOR 0.89 (95% CI 0.80–1.00) • High DBP • AOR 0.82 (95% CI 0.75–0.90) • BW had a negative association with BP across the whole BP range	2.5–4.0 kg	Adjusted for age, gender, height, BW/gestational age, family history of hypertension, parental educational level, family income, region, BMI	Serious	Fair	Good
Yiu et al. (1998), USA (129)	• Cohort • *n* = 2,958	Continuous outcome	• HBW >4,500 g (97^th^ percentile) • Significant inverse relationship between birth weight and SBP. For every 1-kg decrease in BW in term infants, SBP increased by 1.3 mmHg and DBP by 0.6 mmHg	AGA (3rd−97th percentile)	Adjusted for gestational age, race, sex, follow-up height, follow-up weight	Serious	Poor	Poor
**Coronary heart disease (CHD)**, *n* = **1**								
Rashid et al. (2019), USA (130)	• Cohort • *n* = 9,820	• Incident heart failure • *n* = 432	• HBW compared with medium BW: • Incident heart failure: • AHR 1.27 (95% CI 1.05–1.54) • No significant association with all-cause mortality or myocardial infarction	2,500–4,000 g	Adjusted for age, sex, BMI, current and former smoking, ethanol intake, hypertension, diabetes mellitus, left ventricular hypertrophy, income, systolic BP, and high-density lipoprotein	Serious	Fair	Fair
**Atrial fibrillation/other cardio-vascular risk factors**, *n* = **6**								
Conen et al. (2010), USA (131)	• Longitudinal prospective cohort • *n* = 27,982	• Cases AF • *n* = 735	• Risk of AF in BW categories • Adjusted HR • >4,500 vs. <2,500 g • *AHR 1.63 (95% CI 1.07–2.50) • Fully adjusted HR • **AHR 1.29 (95% CI 0.84–1.98) • P-linear trend 0.23	<2,500 g	• *Age, hypercholesterolemia, smoking, exercise, alcohol consumption, education, race, HRT therapy, BMI, SBP, DBP, diabetes • **All above plus adult height, body weight between 18 and 30 years	Serious	Fair	Fair
Johnsson et al. (2018*)*, Sweden (133)	• Cohort, matched • *n* = 644, • only 54 participated	Continuous outcome	• No differences regarding blood pressure, lipid profiles, apolipoproteins, high-sensitivity CRP, or common carotid artery (CCA) wall dimension • Cases: 37% higher intima thickness in radial artery (RA-IT) (*p* < 0.01) and 44% difference in radial intima/media ratio (RA-I:M ratio) (*p* < 0.01)	3,140–3,950 g	RA-IT and RA-I: M adjusted for gender, gestational age, smoking, BMI, systolic and diastolic blood pressure, CRP, and apolipoprotein B/A1 ratio	Critical	Poor	Poor
Larsson et al. (2015), Sweden (132)	• Cohort • *n* = 29,551 men • *n* = 23,454 women	• Cases AF • *n* = 2,711 men • *n* = 1,491 = women	• Risk for atrial fibrillation • Relative risk (RR)+95% CI 4,000–4,999 g • Men • ARR 1.03 (95% CI 0.94–1.15)* • ARR 0.89 (95% CI 0.80–0.99)** • Women • ARR 1.07 (95% CI 0.91–1.27)* • ARR 0.96 (95% CI 0.81–1.14)** • ≥5,000 g • Men • ARR 1.29 (95% CI 1.05–1.58)* • ARR 1.06 (95% CI 0.86–1.30)** • Women • ARR 1.50 (95% CI 1.01–2.24) • ARR 1.21 (95% CI 0.81–1.81)**	2,500–3,999 g	• Adjustments in multivariable logistic regression analysis: • *Age, preterm birth, • **Plus education, smoking status and pack year of smoking, family history of myocardial infarction before 60 years and age, history of coronary heart disease or heart failure, history of hypertension, history of diabetes, BMI, and height	Moderate	Good	Fair
Perkiömäki et al. (2016), Finland (135)	• Cohort • rMSSD: • *n* = 1,799 men • *n* = 2,279 women • BRS: • *n* = 902 men • *n* = 1,020 women	Continuous outcome	• In men higher birth weight was independently associated with poorer cardiac autonomic function [seated (*r* = −0.058, *p* = 0.014) and standing rMSSD (*r* = −0.090, *p* < 0.001), standing BRS (*r* = −0.092, *p* = 0.006)]. Multivariate analysis *p* < 0.05 for all. • Same association was not seen in women	Birth weight	• Vagally mediated heart rate variability (rMSSD, sitting or standing) • Spontaneous baroreflex sensitivity (BRS) at age 46 • Adjusted for: • Continuous adult variables: BMI, height, SBP, DBP, waist–hip ratio, glucose, glycated hemoglobin, total cholesterol, high density cholesterol, triglycerides • Categorized adult variables: current smoking, sitting time, alcohol consumption, sufficiency of sleep, physical activity	Moderate	Good	Good
Skilton et al. (2014), Finland (134)	Cohort	*n* = 696Continuous outcome	• Mean carotid intima thickness: • Adj. beta-coefficient: • 0.022 (95% CI 0.007–0.036) (*p* = 0.003) • No difference in brachial flow mediated dilation, BP between LGA and normal BW	Normal birth weight 50–75th percentile	Adjusted for age, sex, study center, SES, marital status, cardiovascular risk factors, BMI	Moderate	Good	Fair
Timpka et al. (2019), UK (136)	• Longitudinal cohort study • *n* = 1,964	Continuous outcome	Higher BW *z*-scores were associated with small differences of diastolic function in adolescence	*Z*-scores between 10th and 90th percentiles	• Adjusted for maternal pre-pregnancy BMI, age, level of education and smoking during pregnancy • Final model additionally adjusted for factors in adolescence; BMI, SBP, heart rate	Moderate	Fair	Fair

*BW, birth weight; HBW, high birth weight; NBW, normal birth weight; GA, gestational age; LGA, large for gestational age; AGA, appropriate for gestational age; SGA, small for gestational age; BMI, body mass index; HBP, high blood pressure; SBP, systolic blood pressure; DBP, diastolic blood pressure; SES, socioeconomic status; CHD, coronary heart disease; AF, atrial fibrillation; OBP, office blood pressure; HoBP, home blood pressure; AOR, adjusted odds ratio; AHR, adjusted hazard ratio*.

#### Blood Pressure and Hypertension

The SR and meta-analysis by Zhang et al. (19), including 31 studies on the association between high birth weight or LGA and blood pressure or hypertension, showed that high birth weight in younger children (6–12 years) was associated with a higher systolic and diastolic blood pressure, while in older adults (41–60 years) the reverse association was found. The same pattern was seen for the relative risk of hypertension. The authors describe the phenomenon as a “catch-down” effect in the elevation of blood pressure that is observed in subjects with high birth weight as they grow older (19). Hence, older individuals with high birth weight are less likely to develop hypertension than those with normal birth weight (19).

Fourteen original studies (125–138), not included in the review by Zhang et al. (19) were found. Four studies, all with serious risk of bias, showed an inverse relation between high birth weight/LGA and blood pressure, but the mean age of the individuals included in the studies varied tremendously ranging from 6–9 to >50 years of age. Six studies, four with serious and two with moderate risk of bias, showed no association between high birth weight/LGA and blood pressure/hypertension. The two studies with moderate risk of bias included individuals with age ranging from 6–18 years (126) to 33–65 years (129). Finally, four studies, one with moderate risk of bias and three with serious risk of bias, showed that high birth weight/LGA was positively associated with high blood pressure/hypertension. The study with moderate risk of bias included individuals with age 12–15 years (130).

**Conclusion:** There may be an association between high birth weight and hypertension in childhood, low certainty of evidence (GRADE ⊕⊕OO).

There may be an inverse association between high birth weight and hypertension in adulthood, low certainty of evidence (GRADE ⊕⊕OO).

#### Coronary Heart Disease

One SR of high quality including 27 articles on birth weight and CHD in adults was identified (124). A meta-analysis based on six prospective cohort studies on CHD exploring the risk of CHD in high birthweight children found no difference in the risk of CHD in children with high birth weight [OR 0.89 (95% CI 0.79–1.01)] (124). Furthermore, the meta-analysis showed that a 1-kg increase in birth weight is associated with a lower risk of CHD [OR 0.83 (95% CI 0.80–0.86)].

Only one original study (139) from the USA was identified which was not included in the SR.

**Conclusion:** There is probably no difference in the risk of CHD in men and women born with high birth weight compared with adults born with normal birth weight, moderate certainty of evidence (GRADE ⊕⊕⊕O).

#### Atrial Fibrillation and Other Cardiovascular Outcomes

Two studies with serious (140) and moderate risk of bias (141) explored the association between high birth weight and atrial fibrillation in adulthood and found no association.

Two studies found higher thickness of the radial artery intima (142) and the carotid artery intima (143) in adults of high birth weight or LGA while other cardiovascular risk factors and arterial function did not differ. In a Finnish study with moderate risk of bias, men with higher birth weight had a higher risk of poor cardiac autonomic function while the same association was not seen in women (144). Finally, higher BW z-scores were associated with small differences in diastolic function in adolescence in a study with moderate risk of bias (145).

**Conclusion:** It is uncertain if there is an association between high birth weight or LGA and altered cardiovascular function in adulthood, very low certainty of evidence (GRADE ⊕OOO).

### Diabetes

Outcomes are listed in Table 1.4.

**Table 1.4:** LGA and high birth weight and long-term outcomes—type 1 and type 2 diabetes.

**Author, year, country**	**Study design**	**Cases**	**Outcomes (risk estimates)**	**Reference group (weight)**	**Comments/adjustments**	**Risk of bias**	**Directness**	**Precision**
• **Type 1 and type 2 diabetes** • **Systematic review/meta-analysis** ***n*** **=** **6**								
Cardwell et al. (2010), UK (137)	• Type 1 diabetes • Meta-analysis • Cohort *n* = 5 case–control *n* = 20 • 30 populations	12,087	• Birth weight >4,000 g: • OR (cohort studies) 1.15 (95% CI 1.05–1.26) • OR (case–control studies) 1.05 (95% CI 0.95–1.17) • AOR (all studies) 1.11 (95% CI 1.03–1.20)	3,000–3,500 g	All ages included in risk estimates not only children/adolescents <18 years			
Harder et al. (2007), Germany (158)	• Type 2 diabetes • Meta-analysis • cohort *n* = 10 • case–control *n* = 3	6,901	• Birth weight >4,000 g: • ^1^OR 1.27 (95% CI 1.01–1.59) • ^2^OR 1.36 (95% CI 1.07–1.73)	• ^1^ ≤ 4,000 g • ^2^2,500 g • 4,000 g	No separate OR calculated for children/adolescents <18 years			
Harder et al. (2009), Germany (18)	• Type 1 diabetes • Meta-analysis • cohort *n* = 2 • case–control *n* = 10	7,491	• Birth weight >4,000 g: • OR. 1.17 (95% CI 1.09–1.26) • AOR 1.43 (95% CI 1.11–1.85)	<4,000 g	Adjusted for confounders in seven of the included studies and wide difference in the number of confounders ranging from 2 to 14			
Knop et al. (2018), China (160)	• Type 2 diabetes • Systematic review, meta-analysis • 49 studies • Cohort *n* = 36 • Case–control *n* = 8 • Cross-sectional *n* = 5 • (for high birth weight 32 studies)	43,549	• Birth weight >4,500 g: • OR 1.19 (95% CI 1.04–1.36)	4,000–4,500 g	Adult only (>18 years)			
Whincup et al. (2008), UK (159)	Type 2 diabetes systematic review, meta-analysis	6,260	• Per 1,000-g increase: • OR 0.80 (95% CI 0.72–0.89) • Birth weight >4,000 g: • OR 1.35 (95% CI 0.67–2.72)	<4,000 g	Adults			
Zhao et al. (2018), China (161)	• Type 2 diabetes • Meta-analysis, • Cohort *n* = 16 • Case–control *n* = 5	22,341	• Birth weight >4,000 g: • OR was calculated for all ages: • OR 1.11 (95% CI 1.00–1.24)	2,500–4,000 g	Only 2 studies were limited to children/adolescents less than 18 years, both were case–control studies. No separate calculated OR for children/adolescents separately			
**Original articles**								
• **Type 1 diabetes** • **Original articles** *n* =**20**								
Bock et al. (1994), Denmark (144)	Case–control	837	• No statistical differences in mean birth weight between the cases and controls: • 3,381, SD 536 g vs. 3,351, SD 602 g		• Exclusion criteria: mother with IDDM at the time of birth • No risk estimates	Serious	Good	Fair
Borras et al. (2011), Spain (145)	Case–control	306	• LGA >90 percentile • OR for diabetes 1.45 (95% CI 1.02–2.07)	10–90th percentile	• No adjustment • 43 of originally 349 cases excluded due to missing data on birth weight	Serious	Good	Fair
Cardwell et al. (2005), UK (138)	Cohort study	991	• Birth weight >4,000 g: • ARR 1.68 (95% CI 1.30–2.18) • Birth weight 3,500–3,999 g: • ARR 1.48 (95% CI 1.20–1.83)	<3,000 g	• Adjusted for maternal age, birth order, year of birth, gestational age • Missing data 8%	Moderate	Good	Good
Goldacre (2017), UK (139)	Cohort study	2,969	• Birth weight 4,000–5,499 g: • AHR 1.12 (95% CI 0.99–1.27) • Birth weight 3,500–3,999 g: • AHR 1.11 (95% CI 1.02–1.22)	3,000–3,499 g	Adjusted for infant sex, gestational age, maternal type 1 diabetes, maternal obesity, deprivation quintile, and caesarean section	Moderate	Good	Good
Haynes et al. (2007), Australia (146)	Cohort	840	• Birth weight ≥4,000 g: • IRR 1.19 (95% CI 0.95–1.49) • Birth weight 3,500–3,999 g: • IRR 1.09 (95% CI 0.92–1.28)	3,000–3,499 g	Adjusted for maternal age, gestational age, birth order, and year of birth	Moderate	Good	Good
Levins et al. (2007), UK (140)	Cohort	518	• Estimated rate of diabetes (<15 years) in birth weight categories: • 3,500–3,999: Rate 1.55 (95% CI 1.28–1.86) • ≥4,000: Rate 1.65 (95% CI 1.17–2.26)	No ref group	Adjusted for year of birth, Rates only per 1,000 individuals presented. No difference between birth categories	Serious	Good	Fair
Jones et al. (1999), UK (147)	Case–control study	315	• Birth weight 3,500–3,900 g: • ARR 1.00 (95% CI 0.74–1.36) • Birth weight ≥4,000 g: • ARR 1.15 (95% CI 0.76–1.75)	3,000–3,499 g	Adjusted for maternal age, parity, birth weight for gestational age, gestational age and year of birth. Data included in Ievins (1997) and more restricted data material	Moderate	Good	Fair
Khashan et al. (2015), Sweden (141)	Cohort study	13,944	• Birth weight 4,000–5,500 g: • ARR 1.01 (95% CI 0.96–1.05) • LGA (+2 SD above mean) vs. AGA • RR 1.14 (95% CI 1.04–1.24)	3,000–3,999 g	Adjusted for offspring age as a time-dependent variable, year of birth, maternal age, education, BMI, country of origin, pre-gestational diabetes, gestational diabetes and infant sex	Low	Good	Good
Kuchlbauer et al. (2014), Germany (142)	Cohort study	1,117	No risk estimate available. cases with type 1 diabetes had higher birth weight measured as SDS (0.15 vs. 0.03) than the newborn in the control SDS (*z*-scores) are calculated from birth weights based on population reference values		No adjustment. No risk estimates	Critical	Good	Fair
Lawler-Heavner et al. (1994), USA (148)	Case–control study	221	• Birth weight 3,500–3,999 g: • AOR 0.9 (95% CI 0.5–1.7) • Birth weight ≥4,000 g: • AOR 1.0 (95% CI 0.4–2.5)	<3,000 g	Adjusted for sex, age and birth in Colorado	Serious	Good	Fair
McKinney et al. (1999), UK (149)	Case–control study	196	• Birth weight ≥3,500 g: • OR 1.01 (95% CI 0.68–1.51)	2,500–3,000 g	Uncertain whether the results are adjusted or not	Serious	Good	Fair
Metcalfe and Baum (1992), UK (150)	Case–control study	952	• Results given according to proportions in three categories of birth weight: • <2,500: insulin-dependent diabetes mellitus (IDDM) 65 (7%), Office of Population Censuses and Surveys (OPCS) 32,779 (6%) • 2,500–3,999: IDDM 783(82%), OPCS 509707 (86%) • ≥4,000: IDDM 104 (11%), OPCS 46012 (8%)		No adjustments. No risk estimates. No conclusions drawn	Serious	Good	Fair
Patterson et al. (1994), UK (151)	Case–control study	529	• Birth weight ≥4,000 g; • OR 1.14 (95% CI 0.75–1.74)	2,500–3,999 g	No adjustments	Serious	Good	Fair
Rosenbauer et al. (2008), Germany (152)	• Case–control • Nationwide hospital-based surveillance (ESPED)	• 760 • 719 cases in birthweight analysis	• Birth weight ≥4,000 g: • AOR 1.28 (95% CI 0.94–1.73)	3,000–3,999 g	Probably adjusted for familiar type 1 diabetes, social status, maternal age, number of siblings and change of residency	Moderate	Good	Fair
Stene et al. (2001), Norway (143)	Cohort study	1,824	• 3,500–3,999 g: RR 2.11 (95% CI 1.24–3.58) • 4,000–4,499 g: RR 2.38 (95% CI 1.39–4,06) • ≥4,500 g: RR 2.21 (95% CI 1.24–3.94)	<2,000 g	Adjusted for sex, maternal age, plurality, birth weight, gestational age, caesarean section, pre-eclampsia, year of birth	Low	Good	Fair
Stene and Joner (2004), Norway (153)	Case–control study	545	• 3,500–3,999 g: AOR 0.94 (95% CI 0.44–1.99) • ≥4,000 g: AOR 1.01 (95% CI 0.46–2.29)	<2,500 g	Adjusted for sex, maternal age, plurality, birth weight, gestational age, caesarean section, pre-eclampsia, duration of breast feeding, maternal education, atopic eczema, allergic rhino-conjunctivitis and asthma	Low	Good	Fair
Tai et al. (1998), Taiwan (154)	Case–control	117	• Birth weight ≥4,000 g: • AOR 0.97 (95% CI 0.39–2.45)	<3,000 g	Adjusted for age, sex	Critical	Poor	Poor
Wadsworth et al. (1997), UK (155)	Case–control	• 281 • 218 cases included in the analysis	• No significant association with birth weight analyzed as a continuous variable • Unadjusted OR per kg increase in birth weight 0.94 (95% CI 0.65–1.35)		Unadjusted	Serious	Good	Poor
Waernbaum et al. (2019), Sweden (156)	Case–control study	14,949	AOR 1.08 (95% CI 1.06–1.10)	Birth weight z-score category with the interval 0–1 as reference	Adjusted for urinary tract infection, PROM, maternal age, PTB, maternal BMI	Low	Good	Good
Wei et al. (2006), Taiwan (157)	Case–control study	277	≥4,000 g: AOR 1.01 (95% CI 0.46–2.29)	<2,600 g	Adjusted for age, sex, socioeconomy, family history of diabetes„ delivery order, breast feeding, BMI, and GDM	Moderate	Fair	Fair
**Type 2 diabetes**								
Hu et al. (2020), China (163)	Cohort	48,118	≥4,000 g: AOR 1.20 (95% CI 1.07–1.34)	2,500–3,499 g	Adjustments: age, gender, smoking, drinking, education, physical activity, diet habits, systolic blood pressure, dyslipidemia, BMI	Moderate	Fair	Good
Zhu et al. (2013), China (164)	Cross-sectional survey	• 903 children with overweight • 2 with type 2 diabetes • 6 with impaired fasting glucose • 16 with impaired glucose tolerance • 2 with impaired fasting glucose + impaired glucose intolerance	• Birth weight ≥4,000 g: • AOR 1.92 (95% CI 1.06–3.49) • Subgroup of girls analyzed separately: • AOR 4.38 (95% CI 1.21–15.85)	2,500–3,999 g	Adjusted for age, gender, parental education. Only few children with type 2 diabetes or impaired fasting glucose	Moderate	Fair	Fair

*LGA, large-for-gestational-age; AGA, appropriate-for-gestational-age; HOMA-IR, homeostasis model assessment-insulin resistance; MS, metabolic syndrome; GDM, gestational diabetes mellitus; LBW, low birth weight; HBW, high birth weight; NBW, normal birth weight*.

#### Type 1 Diabetes

Two SR and meta-analyses (18, 146) (moderate and low quality), six cohort studies (147–152), and 14 case–control studies (153–166) reported on the association between high birth weight or LGA and type 1 diabetes. Both SR/meta-analyses reported an association between high birth weight and childhood-onset type 1 diabetes [AOR of 1.43 (95% CI 1.11–1.85) and AOR 1.11 (95% CI 1.03–1.20)] (18, 146).

Of the 20 original studies, four were assessed being of low, six of moderate, and the rest of critical or serious risk of bias.

**Figure 9:**
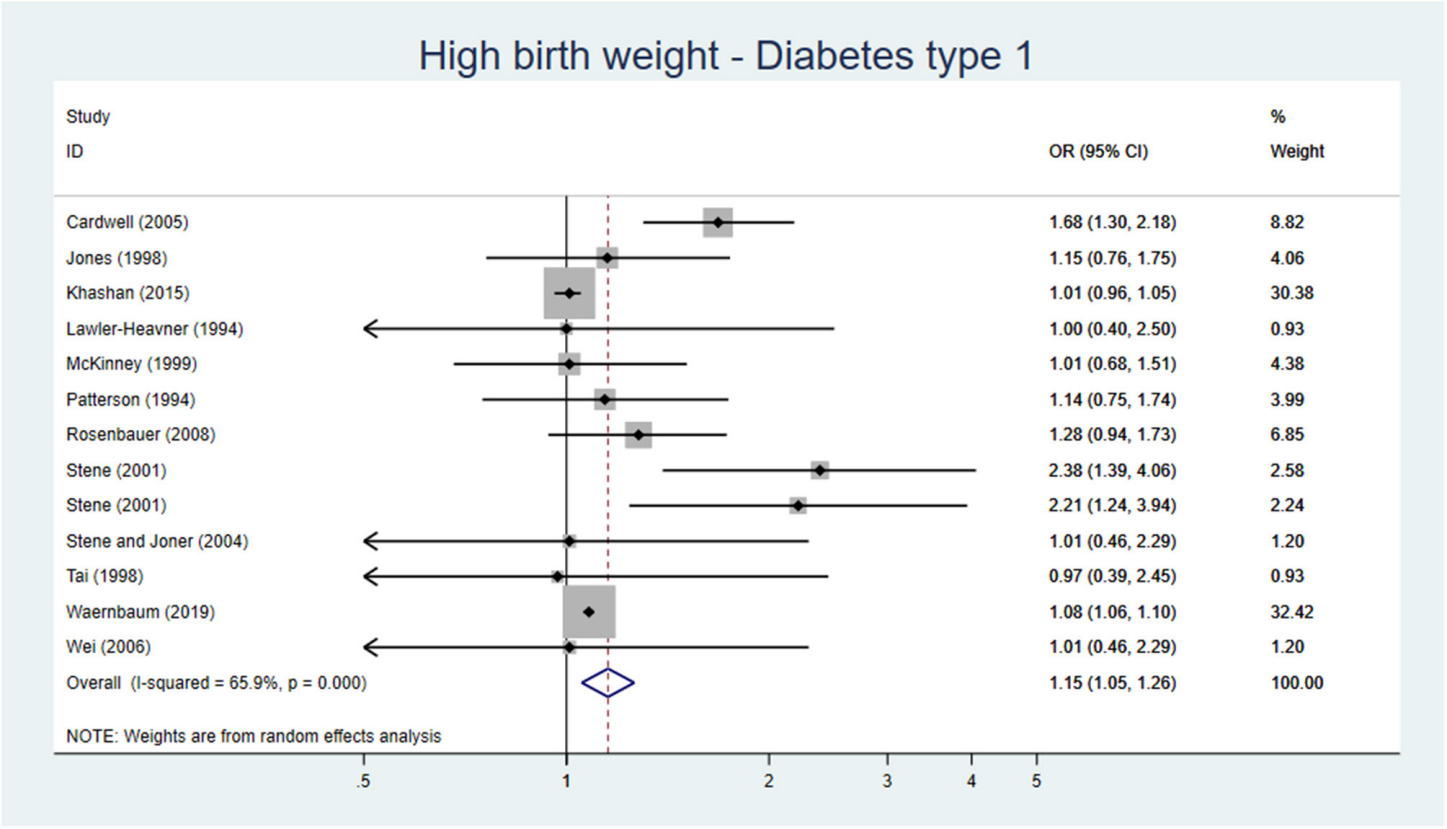
Forest plot describing the association between high birth weight and Diabetes type 1.

**Figure 10:**
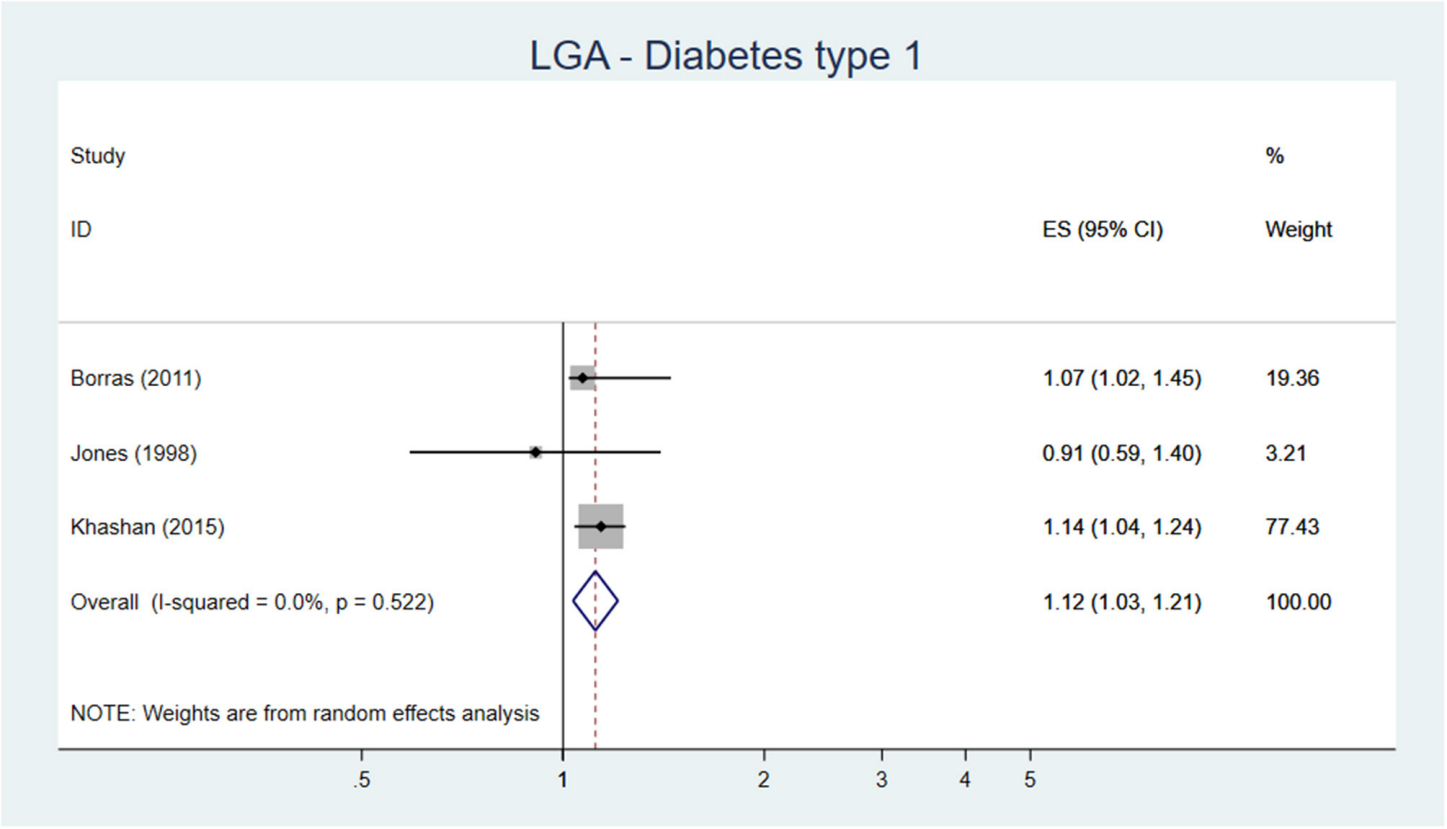
Forest plot describing the association between LGA and Diabetes type 1.

Our meta-analysis, including 13 studies, found a pooled OR of 1.15 (95% CI 1.05–1.26) for type 1 diabetes when comparing birth weight >4,000 g with <4,000 g (Figure 9). For LGA vs. AGA, the OR was 1.1 (95% CI 1.03–1.21) (Figure 10). All but one study (163) included children below 18 years of age. Two of the eight studies not included in the meta-analysis had moderate risks of bias and these studies found no significant association between high birth weight and type 1 diabetes. Other studies not included in the meta-analysis were of serious or critical risk of bias.

**Conclusion:** High birth weight and/or LGA is probably associated with a slight increase in type 1 diabetes, moderate certainty of evidence (GRADE ⊕⊕⊕O).

#### Type 2 Diabetes

Four SR investigated the association between birth weight/high birth weight and type 2 diabetes (167–170). Three of these SR were considered being of high quality (168–170). The literature search identified few additional studies (171, 172). The SR by (168, 170) only included adults while the SR by (167, 169) also included children; however, only in a few studies. The SR by Knop et al. (169) reported a slight increase in type 2 diabetes if birth weight is above 4,500 g, OR 1.19 (95% CI 1.04–1.36), while the SR by Zhao et al. (170) found no increase, OR 1.11 (95% CI 1.00–1.24) for birth weight above 4,000 g. The SR by Knop et al. (169) pointed out the J-shaped association with a higher risk, particularly at low and to a less extent at high birth weight.

**Conclusion:** High birth weight may be associated with a slight increase in type 2 diabetes, low certainty of evidence (GRADE ⊕⊕ OO).

## Discussion

In this systematic review and meta-analysis, we have summarized the evidence for an association between high birth weight and/or LGA and some severe long-term outcomes for the children (Supplementary Table 5). The outcomes included are malignancies in childhood and breast cancer, cardiovascular diseases, psychiatric disorders, and diabetes type 1 and 2. To clarify if such associations exist and if so, the magnitude of these associations is of high importance for children born after spontaneous conception in view of the dramatic increase in obesity among women of childbearing age and the associated rise in high birth weight babies. In ART, these findings are important due to the increase in frozen/thawed cycles in ART and the recent findings of higher risks of high birth weight and LGA in offspring from FET cycles.

The systematic literature search identified a huge number of articles which were scrutinized and 173 of these publications were selected for this review.

The choice of the selected types of malignancies was based on the number of publications. Thus, our SR does not include all types of malignancies, but the ones where most publications were identified. The metabolic part was limited to diabetes type 1 and 2. Cardiovascular and psychiatric diseases were selected due to being common in the population and having a high impact on human health.

### Malignancies

We found a small to moderately increased risk for all types of malignancies studied, with estimates of OR between 1.19 and 1.69. The most pronounced association was found for Wilm's tumor. The biological mechanism linking fetal growth and cancer is largely unknown (51). The observation in children with overgrowth disorders, such as Beckwith–Wiedemann syndrome (BWS), supports a theory that the number, size, and proliferative potential of muscle stem cells (173) which correlate with birth weight are involved. These cells are particularly susceptible to oncogenic mutations and thus a faster growing fetus may involve an increased cancer risk. BWS children, characterized by increased fetal growth, are prone to a wide range of cancers, including Wilm's tumor and leukemia (174). BWS is caused by overexpression of insulin-like factor 2 (IGF-2) gene. Furthermore, several cancers in adults are associated with increased levels of IGFs. Since IGF levels also are increased in heavy babies without these syndromes, there may be a more general association between levels of IGF in newborns and risk of childhood cancer (175). Further support for the IGF-1 theory comes from a study on children with congenital IGF-1 deficiency who seems to be protected against the risk of developing cancer (176). Other suggested mechanisms include exposure of fast-growing babies to elevated levels of estrogen *in utero* and/or epigenetic mechanisms, both associated with fetal birth weight and cancer risk (177).

### Psychiatric Disorders

Four out of six cohort studies on high birth weight and/or LGA and schizophrenia reported an increased risk of developing schizophrenia in the offspring while no association was found in two studies in adjusted models. All these studies were performed in the Nordic countries and the limit for being born with high birth weight varied being >4,000 and >4,500 g. The mechanisms underlying the association between high birth weight and schizophrenia are unclear. It has been suggested that potential fetal exposure to gestational diabetes may play a role, as an association between maternal diabetes and schizophrenia among offspring has been found (94, 178). Furthermore, gestational diabetes may lead to macrosomic babies, who are at increased risk of delivery complications such as shoulder dystocia and asphyxia which also, *per se*, may increase a risk to later psychiatric problems (12, 94). Interestingly, in a study using self-report questionnaires, high birth weight increased the risk for depression only in women (99).

Interestingly, a recent systematic review suggested that high birth weight was protective of psychotic disorders in general (100). It was, however, unclear which studies were included in this review and no quality assessment was presented.

In many studies, several types of psychiatric disturbances were investigated and even combined. This may explain the contradictory results concerning high birth weight/LGA and psychiatric disorders. Environmental and socioeconomic status probably play an important role in a person's susceptibility for a psychiatric disease making those with higher socioeconomic status less vulnerable (102).

There might be an association between high birth weight/LGA and negative behaviors in adolescence. The reasons for this connection are largely unknown. Family and genetic factors certainly are important in the tendency of developing behavior problems, but the neurobiological mechanisms underlying interactions to high birth weight are unclear (106). Due to delivery complications, the macrosomic infants have an increased risk of birth trauma and asphyxia (12, 176). Such adverse perinatal outcomes are, *per se*, associated with later behavioral problems (179).

Most of the studies about intellectual performance and high birth weight have been carried out on male conscripts generally excluding women and part of the most vulnerable men. A reassuring notice was that no association was found between high birth weight/LGA and risk of intellectual disability, or low IQ score. However, according to Lundgren et al. (119) high BMI in adulthood had a negative effect on IQ level.

Cognitive performance was positively related to high birth weight at least up to the birth weight of 4,200 g (113). This association is thought to be mediated by optimal prenatal factors and healthy nutrition both pre- and postnatally. Such findings related to mental development emphasize the importance of maternal care during pregnancy (113).

### Cardiovascular Diseases

Based on the current evidence, there may be an age-related association between high birth weight/LGA and high blood pressure in childhood while the opposite is found in adulthood. For CHD or cardiovascular function in adults, there was no obvious association with high birth weight or LGA. In the study by Wang et al. (124) the focus was on the relation between birth weight and CHD over the full birth weight range from low to high birth weight, and they found a consistent inverse relation between birth weight and CHD.

In general, individuals with high birth weight are taller and heavier later in life than subjects with normal birth weight (180). However, their metabolic health seems to be better later in life as they have less adipose tissue than lean mass (181).

Contradictory to the findings of a lower risk of CHD in children born with high birth weight, babies are more likely to be born large-for-gestational age in mothers with diabetes, increasing the risk of diabetes and CHD later in the children's life (18). High birth weight could be a result of gestational diabetes in the mother thus, hypothetically, high birth weight may be a potential risk factor of CHD in the offspring (182, 183).

### Type 1 Diabetes

In our meta-analysis, high birth weight was associated with a slightly higher risk of type 1 diabetes in line with previous meta-analyses (18, 146).

The mechanism between high birth weight and type 1 diabetes seems unknown. It may be other factors besides the birth weight *per se* that are responsible for this association. Gestational diabetes and maternal overweight during pregnancy are risk factors for increased birth weight (184, 185). It has been suggested that maternal and/or fetal hyperglycemia also may predispose to an increased susceptibility of the overstimulated fetal pancreatic beta cells to processes causing type 1 diabetes (186, 187). Furthermore, a rapid postnatal growth during the first year of life also seem to be associated with a later risk of developing type 1 diabetes (18). Other triggering factors of the genetic predisposition may also be related to the association between high birth weight and type 1 diabetes (188).

### Type 2 Diabetes

For type 2 diabetes, recently performed meta-analyses of high quality found some divergent results. Knop et al. (169) identified a small but significant increase in risk of type 2 diabetes at birth weight above 4,500 g while the meta-analysis by Zhao et al. (170), could not identify any increased risk; however, the estimate was of borderline significance. The biological mechanism behind such an association, if it exists, is a matter of debate. According to the fetal programming hypothesis, also small changes in organ maturation during the fetal period might result in altered growth and disordered endocrine function in adulthood (169).

## Strengths and Limitations

The major strength of this systematic review is the comprehensive literature search, identifying a considerable number of relevant articles. The ability to present meta-analyses, either of high quality and recently published or new meta-analyses performed for the purpose of this SR, makes interpretation of the summarized literature easier to capture. The main limitation is that all data are based on observational studies, both cohort studies being of higher quality but also case–control studies with their inborn risk of selection bias. Our conclusions are, however, based mainly on meta-analyses and/or on studies with low risk of bias.

In conclusion, this systematic review and meta-analysis, investigating high birth weight and LGA as risk factors for adverse outcome in offspring, found elevated risks for certain malignancies in childhood, breast cancer, several psychiatric disorders, hypertension in childhood, although not in adulthood, and type 1 and type 2 diabetes. Although these risks represent serious health effects, both in childhood and in adulthood, the size of these effects seems moderate. The results are important for the overall implications of increasing birth weight and will contribute to the ongoing discussion of the pros and cons of fresh or frozen embryo transfer cycles in ART.

## Data Availability Statement

The original contributions presented in the study are included in the article/Supplementary Materials, further inquiries can be directed to the corresponding author.

## Author Contributions

ÅM, HL, AL, NO, AP, LR, VS-A, and CB contributed to conception and design of the study. ÅM and CB search databases. Screening of abstracts and of full papers for inclusion was done by pairs of reviewers by ÅM, HL, AL, NO, AP, LR, VS-A, and CB. MP performed the statistical analysis. ÅM, HL, AL, NO, AP, LR, VS-A, MP, and CB wrote sections of the manuscript. All authors contributed to manuscript revision, read, and approved the submitted version.

## Conflict of Interest

The authors declare that the research was conducted in the absence of any commercial or financial relationships that could be construed as a potential conflict of interest.
