# Supplementary material for: The Association Between High Birth Weight and Long-Term Outcomes—Implications for Assisted Reproductive Technologies: A Systematic Review and Meta-Analysis
Source: Front Pediatr. 2021 Jun 23;9:675775. doi: 10.3389/fped.2021.675775 (PMC8260985; doi:10.3389/fped.2021.675775)
Supplement: Supplementary file 1 [file Data_Sheet_1.zip › Supplement Table 2.2. Excluded Finland_hl och vsa 200918_ 201003AN., A╠èM 210220.docx]

**Supplementary Table 2.2. Excluded articles - psychiatric disorders**

| **Study**  **author, journal, publication year, country** | **Reason for exclusion** |
| --- | --- |
| Broekman et al., Pediatrics 2009, Singapore | Infants with birth weight >4 kg were excluded from the study |
| Carlens et al., Ann Rheum Dis 2009, Sweden | Rheumatoid arthritis and juvenile idiopathic arthritis |
| Carter et al., Arch Dis Child 2019, Canada | Asthma in childhood and adolescence |
| Colebatch and Edwards, Clinical & Experimental Immunol 2010, UK | Review article, wrong outcome |
| Di Giovanni et al., Acta pediatr 2017, Italy | Pubertal growth |
| Ester et al., Int J Epidemiol 2019, The Netherlands | Results on childhood eating disorders at a mean age of 4 years |
| Goodman et al., Am J Epidemiol 2013, Sweden | Eating disorder |
| Grissom NM and Reyes TM, Int J Devi Neuroscience 2013, UK | Review article; animal studies |
| Kandhall and Miller, Minerva Psichiatr 2013, USA | Review |
| Kerkhof et al.,Clin Exp Allergy 2003, The Netherlands | Atopic dermatitis in the first year of life |
| Moilanen K, Schizophrenia Research 2002; Finland | Abstract, double publication |
| Mumm et al., Fertil Steril 2013, Denmark | Polycystic ovary syndrome in adult life |
| Panduru et al, Acta Dermatovenerol Croat 2014, Romania | Systematic review, atopic dermatitis |
| Pinto et al., Clin Exp Allergy 2017, The Netherlands | The prevalence of asthma at age of 8 years in atopic and non-atopic children |
| Remes et al., Pediatr Allergy Immunol 2008, Finland | Asthma and atopy at the age of 16 years |
| Ridgway et al., Medicine &Science in Sports & Exercise 2008, UK | Wrong outcome |
| Schams et al., Eur J Pediatr 2017, Switzerland | Developmental dysplasia of the hip with the hip maturity at the age of 2-5 days |
| Simard et al., Arthritis Rheum 2008, USA | Incident systemic lupus erythematosus |
| Sin et al., Arch Pediatr Adolesc Med 2004, Canada | Risk of emergency visit for asthma during childhood, follow up 10 years |
| Solis-Urra et al., Neurolmage 2019 | Gray matter volume |
| Spiegel et al., Clin Exp Allergy 2019, Israel | The incidence of long-term pediatric hospitalizations due to endocrine morbidity until 18 years of age |
| Tedner et al., Clin Exp Allergy 2010 | Review article, wrong outcome |
| Torniainen et al., Psychol Medicine 2013, Finland | Only persons with schizophrenia studied |
| Törnqvist and Källen, Pediatr and perinatal epidemiol 2004, Sweden | Visual impairment |
| Wang Y el al., Pediatr Obes 2013, USA | No specific reports on infants with high birth weight; wrong outcome |
| Wennerström et al., PLOS one 2015 | Wrong outcome |
| von Bonsdorff et al., PLOS one 2015; Finland | Not specifically separating large for gestational age |
