# Supplementary material for: The Association Between High Birth Weight and Long-Term Outcomes—Implications for Assisted Reproductive Technologies: A Systematic Review and Meta-Analysis
Source: Front Pediatr. 2021 Jun 23;9:675775. doi: 10.3389/fped.2021.675775 (PMC8260985; doi:10.3389/fped.2021.675775)
Supplement: Supplementary file 1 [file Data_Sheet_1.zip › Supplement Table 3.2. Robins eye_ Finland_hl och vsa 200918, A╠èM 210220.docx]

**Supplementary Table 3.2. Psychiatric disorders**

**Bias assessment according to ROBINS-I**

| **Cognitive** | |
| --- | --- |
| **Domains of bias (Alati, 2009)** | **Risk of bias** |
| Bias due to confounding | Low |
| Bias in the selection of participants into the study | Moderate |
| Bias in the classification of interventions | Low |
| Bias due to deviations from intended interventions | Low |
| Bias due to missing data | Low |
| Bias in the measurement of outcome | Low |
| Bias in the selection of reported result | Low |
| **Overall risk of bias** | **Moderate** |
| **Domains of bias (Bergvall, 2006)** | **Risk of bias** |
| Bias due to confounding | Moderate |
| Bias in the selection of participants into the study | Low |
| Bias in the classification of interventions | Low |
| Bias due to deviations from intended interventions | Low |
| Bias due to missing data | Low |
| Bias in the measurement of outcome | Low |
| Bias in the selection of reported result | Low |
| **Overall risk of bias** | **Moderate** |
| **Domains of bias (Buschgens, 2009)** | **Risk of bias** |
| Bias due to confounding | Low |
| Bias in the selection of participants into the study | Low |
| Bias in the classification of interventions | Low |
| Bias due to deviations from intended interventions | Low |
| Bias due to missing data | Low |
| Bias in the measurement of outcome | Low |
| Bias in the selection of reported result | Low |
| **Overall risk of bias** | **Low** |
| **Domains of bias (Daves, 2015)** | **Risk of bias** |
| Bias due to confounding | Moderate |
| Bias in the selection of participants into the study | Serious |
| Bias in the classification of interventions | Low |
| Bias due to deviations from intended interventions | Low |
| Bias due to missing data | Low |
| Bias in the measurement of outcome | Low |
| Bias in the selection of reported result | Low |
| **Overall risk of bias** | **Serious** |
| **Domains of bias (Duffany, 2020)** | **Risk of bias** |
| Bias due to confounding | Low |
| Bias in the selection of participants into the study | Low |
| Bias in the classification of interventions | Low |
| Bias due to deviations from intended interventions | Low |
| Bias due to missing data | Moderate |
| Bias in the measurement of outcome | Low |
| Bias in the selection of reported result | Low |
| **Overall risk of bias** | **Moderate** |
| **Domains of bias (Eide, 2007)** | **Risk of bias** |
| Bias due to confounding | Moderate |
| Bias in the selection of participants into the study | Low |
| Bias in the classification of interventions | Low |
| Bias due to deviations from intended interventions | Low |
| Bias due to missing data | Low |
| Bias in the measurement of outcome | Low |
| Bias in the selection of reported result | Low |
| **Overall risk of bias** | **Moderate** |
| **Domains of bias (Flensborg-Madsen & Mortensen, 2017)** | **Risk of bias** |
| Bias due to confounding | Moderate |
| Bias in the selection of participants into the study | Moderate |
| Bias in the classification of interventions | Low |
| Bias due to deviations from intended interventions | Low |
| Bias due to missing data | Moderate |
| Bias in the measurement of outcome | Moderate |
| Bias in the selection of reported result | Low |
| **Overall risk of bias** | **Moderate** |
| **Domains of bias (Haglund & Källen, 2011)** | **Risk of bias** |
| Bias due to confounding | Low |
| Bias in the selection of participants into the study | Moderate |
| Bias in the classification of interventions | Low |
| Bias due to deviations from intended interventions | Low |
| Bias due to missing data | Low |
| Bias in the measurement of outcome | Moderate |
| Bias in the selection of reported result | Moderate |
| **Overall risk of bias** | **Moderate** |
| **Domains of bias (Kristensen, 2014)** | **Risk of bias** |
| Bias due to confounding | Low |
| Bias in the selection of participants into the study | Moderate |
| Bias in the classification of interventions | Low |
| Bias due to deviations from intended interventions | Low |
| Bias due to missing data | Low |
| Bias in the measurement of outcome | Moderate |
| Bias in the selection of reported result | Low |
| **Overall risk of bias** | **Moderate** |
| **Domains of bias (Leonard, 2008)** | **Risk of bias** |
| Bias due to confounding | Low |
| Bias in the selection of participants into the study | Low |
| Bias in the classification of interventions | Low |
| Bias due to deviations from intended interventions | Low |
| Bias due to missing data | Low |
| Bias in the measurement of outcome | Low |
| Bias in the selection of reported result | Moderate |
| **Overall risk of bias** | **Moderate** |
| **Domains of bias (Lundgren, 2003)** | **Risk of bias** |
| Bias due to confounding | Moderate |
| Bias in the selection of participants into the study | Moderate |
| Bias in the classification of interventions | Low |
| Bias due to deviations from intended interventions | Low |
| Bias due to missing data | Low |
| Bias in the measurement of outcome | Low |
| Bias in the selection of reported result | Moderate |
| **Overall risk of bias** | **Moderate** |
| **Domains of bias (Moore, 2012)** | **Risk of bias** |
| Bias due to confounding | Low |
| Bias in the selection of participants into the study | Moderate |
| Bias in the classification of interventions | Low |
| Bias due to deviations from intended interventions | Low |
| Bias due to missing data | Low |
| Bias in the measurement of outcome | Moderate |
| Bias in the selection of reported result | Low |
| **Overall risk of bias** | **Moderate** |
| **Domains of bias (Powers 2006)** |  |
| Bias due to confounding | Moderate |
| Bias in the selection of participants into the study | Low |
| Bias in the classification of interventions | Low |
| Bias due to deviations from intended interventions | Low |
| Bias due to missing data | Low |
| Bias in the measurement of outcome | Low |
| Bias in the selection of reported result | Low |
| **Overall risk of bias** | **Moderate** |
| **Domains of bias (Record, 1969)** | **Risk of bias** |
| Bias due to confounding | Moderate |
| Bias in the selection of participants into the study | Low |
| Bias in the classification of interventions | Low |
| Bias due to deviations from intended interventions | Low |
| Bias due to missing data | Low |
| Bias in the measurement of outcome | Low |
| Bias in the selection of reported result | Low |
| **Overall risk of bias** | **Moderate** |
| **Domains of bias (Richards, 2001)** | **Risk of bias** |
| Bias due to confounding | Moderate |
| Bias in the selection of participants into the study | Low |
| Bias in the classification of interventions | Low |
| Bias due to deviations from intended interventions | Low |
| Bias due to missing data | Moderate |
| Bias in the measurement of outcome | Moderate |
| Bias in the selection of reported result | Low |
| **Overall risk of bias** | **Moderate** |
| **Domains of bias (Räikkönen 2013)** | **Risk of bias** |
| Bias due to confounding | Low |
| Bias in the selection of participants into the study | Low |
| Bias in the classification of interventions | Low |
| Bias due to deviations from intended interventions | Low |
| Bias due to missing data | Low |
| Bias in the measurement of outcome | Low |
| Bias in the selection of reported result | Low |
| **Overall risk of bias** | **Low** |
| **Domains of bias (Sörensen 1997)** | **Risk of bias** |
| Bias due to confounding | Low |
| Bias in the selection of participants into the study | Moderate |
| Bias in the classification of interventions | Low |
| Bias due to deviations from intended interventions | Low |
| Bias due to missing data | Low |
| Bias in the measurement of outcome | Low |
| Bias in the selection of reported result | Low |
| **Overall risk of bias** | **Moderate** |
| **Domains of bias (Tamai 2000)** | **Risk of bias** |
| Bias due to confounding | Low |
| Bias in the selection of participants into the study | Low |
| Bias in the classification of interventions | Low |
| Bias due to deviations from intended interventions | Low |
| Bias due to missing data | Moderate |
| Bias in the measurement of outcome | Low |
| Bias in the selection of reported result | Low |
| **Overall risk of bias** | **Moderate** |
| **Domains of bias (van Mil 2015)** | **Risk of bias** |
| Bias due to confounding | Low |
| Bias in the selection of participants into the study | Low |
| Bias in the classification of interventions | Low |
| Bias due to deviations from intended interventions | Low |
| Bias due to missing data | Low |
| Bias in the measurement of outcome | Moderate |
| Bias in the selection of reported result | Low |
| **Overall risk of bias** | **Moderate** |
| **Domains of bias (Yang, 2019)** | **Risk of bias** |
| Bias due to confounding | Serious |
| Bias in the selection of participants into the study | Moderate |
| Bias in the classification of interventions | Low |
| Bias due to deviations from intended interventions | Low |
| Bias due to missing data | Low |
| Bias in the measurement of outcome | Moderate |
| Bias in the selection of reported result | Moderate |
| **Overall risk of bias** | **Serious** |
| **Domains of bias (Zhang, 2020)** | **Risk of bias** |
| Bias due to confounding | Low |
| Bias in the selection of participants into the study | Low |
| Bias in the classification of interventions | Low |
| Bias due to deviations from intended interventions | Low |
| Bias due to missing data | Moderate |
| Bias in the measurement of outcome | Low |
| Bias in the selection of reported result | Low |
| **Overall risk of bias** | **Moderate** |
| **Psychiatric disorders** | |
| **Domains of bias (Gunnell, 2003)** | **Risk of bias** |
| Bias due to confounding | Low |
| Bias in the selection of participants into the study | Moderate |
| Bias in the classification of interventions | Low |
| Bias due to deviations from intended interventions | Low |
| Bias due to missing data | Low |
| Bias in the measurement of outcome | Low |
| Bias in the selection of reported result | Low |
| **Overall risk of bias** | **Moderate** |
| **Domains of bias (Herva, 2008)** | **Risk of bias** |
| Bias due to confounding | Low |
| Bias in the selection of participants into the study | Low |
| Bias in the classification of interventions | Low |
| Bias due to deviations from intended interventions | Low |
| Bias due to missing data | Low |
| Bias in the measurement of outcome | Moderate |
| Bias in the selection of reported result | Low |
| **Overall risk of bias** | Moderate |
| **Domains of bias (Keskinen, 2013)** | **Risk of bias** |
| Bias due to confounding | Low |
| Bias in the selection of participants into the study | Low |
| Bias in the classification of interventions | Low |
| Bias due to deviations from intended interventions | Low |
| Bias due to missing data | Low |
| Bias in the measurement of outcome | Low |
| Bias in the selection of reported result | Low |
| **Overall risk of bias** | **Low** |
| **Domains of bias (Lahti, 2015)** | **Risk of bias** |
| Bias due to confounding | Low |
| Bias in the selection of participants into the study | Low |
| Bias in the classification of interventions | Low |
| Bias due to deviations from intended interventions | Low |
| Bias due to missing data | Low |
| Bias in the measurement of outcome | Low |
| Bias in the selection of reported result | Low |
| **Overall risk of bias** | **Low** |
| **Domains of bias (Liuhanen, 2018)** | **Risk of bias** |
| Bias due to confounding | Low |
| Bias in the selection of participants into the study | Low |
| Bias in the classification of interventions | Low |
| Bias due to deviations from intended interventions | Low |
| Bias due to missing data | Low |
| Bias in the measurement of outcome | Low |
| Bias in the selection of reported result | Low |
| **Overall risk of bias** | **Low** |
| **Domains of bias (Moilanen, 2010)** | **Risk of bias** |
| Bias due to confounding | Low |
| Bias in the selection of participants into the study | Low |
| Bias in the classification of interventions | Low |
| Bias due to deviations from intended interventions | Low |
| Bias due to missing data | Low |
| Bias in the measurement of outcome | Low |
| Bias in the selection of reported result | Low |
| **Overall risk of bias** | **Low** |
| **Domains of bias (Perquier, 2014)** | **Risk of bias** |
| Bias due to confounding | Low |
| Bias in the selection of participants into the study | Low |
| Bias in the classification of interventions | Low |
| Bias due to deviations from intended interventions | Low |
| Bias due to missing data | Low |
| Bias in the measurement of outcome | Low |
| Bias in the selection of reported result | Low |
| **Overall risk of bias** | **Low** |
| **Domains of bias (van Lieshout, 2020)** | **Risk of bias** |
| Bias due to confounding | Moderate |
| Bias in the selection of participants into the study | Low |
| Bias in the classification of interventions | Low |
| Bias due to deviations from intended interventions | Low |
| Bias due to missing data | Low |
| Bias in the measurement of outcome | Low |
| Bias in the selection of reported result | Low |
| **Overall risk of bias** | **Moderate** |
| **Domains of bias (Wegelius, 2011)** | **Risk of bias** |
| Bias due to confounding | Moderate |
| Bias in the selection of participants into the study | Low |
| Bias in the classification of interventions | Low |
| Bias due to deviations from intended interventions | Low |
| Bias due to missing data | Low |
| Bias in the measurement of outcome | Low |
| Bias in the selection of reported result | Low |
| **Overall risk of bias** | **Moderate** |
| **Domains of bias (Wegelius, 2013)** | **Risk of bias** |
| Bias due to confounding | Moderate |
| Bias in the selection of participants into the study | Low |
| Bias in the classification of interventions | Low |
| Bias due to deviations from intended interventions | Low |
| Bias due to missing data | Low |
| Bias in the measurement of outcome | Low |
| Bias in the selection of reported result | Low |
| **Overall risk of bias** | **Moderate** |
