# Supplementary material for: The Association Between High Birth Weight and Long-Term Outcomes—Implications for Assisted Reproductive Technologies: A Systematic Review and Meta-Analysis
Source: Front Pediatr. 2021 Jun 23;9:675775. doi: 10.3389/fped.2021.675775 (PMC8260985; doi:10.3389/fped.2021.675775)
Supplement: Supplementary file 1 [file Data_Sheet_1.zip › Supplementary Table 2.1. excluded studies cancer, A╠èM 210207,210220.docx]

**Table 2.1. Excluded articles - malignancies**

| **Study**  **author, publication year** | **Reason for exclusion** |
| --- | --- |
| **Cancer** |  |
| Aggarwal, Indian Pediatr, 1998 | Wrong outcome |
| Berstein, Advances in Cancer Research, 1988 | Review, no SR |
| Brown, Family Planning Perspectives, 2001 | Review, no SR |
| Bukowski, Plos One, 2012 | Wrong exposure |
| De Fine Licht, Int J Cancer, 2012 | Wrong outcome |
| Dor, New York State Journal of Medicine, 1984 | Wrong outcome |
| Ekbom, Lancet, 1992 | Wrong exposure |
| Ekbom, J Natl Cancer Inst, 1997 | Wrong exposure |
| Ferguson, AEP, 2000 | Abstract |
| Chokkalingam, Cancer Causes Control, 2012 | Wrong exposure |
| Cnattingus, Cancer Epidemiol Biomarkers Prev, 2009 | Wrong outcome |
| Ferguson, Breast Cancer Symposium, 2001 | Abstract |
| Giuffré, Child´s Nerv Syst, 1990 | Wrong exposure |
| Gruhn, Leukemia, 2008 | Wrong exposure |
| Hilakivi-Clarke, Br J Cancer, 2001 | Wrong population |
| Hodgson, Breast Cancer Res, 2004 | Wrong exposure |
| Hubinette, In t J Cancer, 2001 | Wrong exposure |
| Kaijser, J Natl Cancer Inst, 2001 | Wrong exposure |
| Kaijser, Br J Cancer, 2003 | Wrong population |
| Kar, European Journal of Epidemiology, 2019 | Wrong exposure |
| Kehm, Pediatr Blood Cancer, 2017 | Wrong exposure |
| Leong, Int J Cancer, 2003 | Wrong exposure |
| McCormack, Int J Cancer, 2005 | Wrong outcome |
| Steiner, ajog.org, 2018 | Abstract |
| Milne, Int J Cancer, 2008 | Wrong exposure |
| Milne, British Journal of Cancer, 2008 | Wrong exposure |
| Milne, American Journal of Epidemiology, 2009 | Wrong exposure |
| Milne, Int J Cancer, 2013 | Meta-analysis consortium, no SR |
| Morois, International Journal of Epidemiology, 2011 | Wrong outcome |
| Morois, International Journal of Epidemiology, 2011 | Wrong outcome |
| Murray, British Journal of Cancer, 2002 | Wrong exposure |
| Okasha, Best Practice&Research Clin Endocrin and Metabol, 2002 | Review, no SR |
| Quach, Neurotoxicology, 2017 | Duplicate (Harder 2008) |
| Richiardi, British Journal of Cancer, 2002 | Wrong outcome |
| Richiardi, Cancer Epidemiology, Biomarkers and Prevention, 2003 | Wrong outcome |
| Ross, Cancer Causes and Control, 1996 | Wrong exposure |
| Ross, Cancer Epidemiol Biomarkers Prev, 2006 | Editorial |
| Samaras, Journal of the National Medical Association, 2003 | Review, no SR |
| Tamimi, Cancer Cause Control, 2010 | Wrong exposure |
| Tibblin, Epidemiology, 1995 | Wrong outcome |
| Trabert, Gynecologic Oncology, 2018 | Wrong outcome |
| Troisi, British Journal of Cancer, 2006 | Wrong exposure |
| Wang, Int J Colorectal disease, 2014 | Wrong outcome, letter to the editor |
| Wang, nature.com/scientific reports, 2017 | Small number of cases |
| Yaezel, The Journal of Pediatrics, 1997 | Wrong exposure |
| Zimmermann, Int J Cancer, 2015 | Wrong outcome |
