# Supplementary material for: The Association Between High Birth Weight and Long-Term Outcomes—Implications for Assisted Reproductive Technologies: A Systematic Review and Meta-Analysis
Source: Front Pediatr. 2021 Jun 23;9:675775. doi: 10.3389/fped.2021.675775 (PMC8260985; doi:10.3389/fped.2021.675775)
Supplement: Supplementary file 1 [file Data_Sheet_1.zip › Supplementary Table 2.3. Excluded studies_Cardiovascular, A╠èMN 210220.docx]

**Supplementary Table 2.3 Excluded articles – Cardiovascular diseases**

| **Study**  **Author, publication, year** | **Reason for exclusion** |
| --- | --- |
| Ashtree, 2020, Nutr Metab Cardiovasc Dis. | Wrong outcome. SR twin studies |
| Bruno, 2015, Curr Opin Cardiol | Narrative review |
| Carter, 2019, Arch Dis Child | Wrong outcome (astma) |
| Chen, 2008, Journal of Pediatric Health Care | Wrong outcome |
| Chiavaroli, 2014, Plos One | Included in Zhang 2014 |
| Gunnarsdottir, 2002, Am J Clin Nutr | Included in Wang 2014 |
| Gunnarsdottir, 2004, Eur J Clin Nutr | Same outcome (BP) as Gunnarsdottir 2002A. Linear model adjusted for truncal fact evt. Bruges I discussion |
| Curhan, 1996, Circulation | Included in Zhang 2013 |
| Huxley, 2007, The Lancet | Systematic review and meta-analysis. All articles included in Wang 2014. |
| Järvelin, 2004, Hypertension | Included in Zhang 2013 |
| Kernell, 2014, BMC Pregnancy and Childbirth | Wrong outcome (congenital heart disease in men) |
| Litwin, 2006, Pediatr Nephrol | Wrong outcome (children with essential hypertension and left ventricular hypertrophy and arterial wall thickening) |
| Radford, 1976, Archives of Disease in Childhood, | Wrong outcome (congenital paroxysmal atrial tachycardia) |
| Rich-Edwards, 2005 BMJ | Included in Wang 2014 |
| Singhal, 2003, Am J Clin Nutr | Wrong outcome (obesity) |
| Taine, 2016, Hypertension | Wrong outcome (BW z-score, growth velocity and BP SD score) |
| Turk, 2014, Rheum Dis Clin N Am | Wrong outcome (RA) |
| Wei, 2007, Obesity | Included in Zhang 2013 |
