# Supplementary material for: The Association Between High Birth Weight and Long-Term Outcomes—Implications for Assisted Reproductive Technologies: A Systematic Review and Meta-Analysis
Source: Front Pediatr. 2021 Jun 23;9:675775. doi: 10.3389/fped.2021.675775 (PMC8260985; doi:10.3389/fped.2021.675775)
Supplement: Supplementary file 1 [file Data_Sheet_1.zip › Supplementary Table 2.4 excluded studies diabetes A╠èM210220.docx]

**Supplementary Table 2.4** **Excluded articles - type 1 and type 2 diabetes.**

| **Study**  **author, publication year** | **Reason for exclusion** |
| --- | --- |
| Bereket and Atay, J Clin Res Pediatr Endocrinol, 2012 | Review, not systematic search |
| Bukara-Radujkovic, Horm Res, 2006 | Abstract |
| Cameron, Curr Obes Rep, 2015 | Review, about socioeconomic position (SEP) and early-life predictors of obesity |
| Dahlquist, Diabetes Care, 1999 | No information about weight groups |
| Desai, Curr Obes Rep, 2012 | Review, not systematic search |
| Dor, NY State J Med, 1984 | Wrong outcome |
| Dyck, Canadian J of Public Health, 2001 | Included in SR by Zhao 2018 |
| Geraghty, BJOG, 2019 | E-poster only |
| Gimeno, Diabetes Care,1997 | Wrong outcome |
| Hammami, J Pediatr, 2001 | Body composition in neonates <2 days after birth |
| Harder, Book-chapter, 2009, | Book chapter including same publications as in Harder et al., 2007 |
| Juul, Obesity Reviews, 2017 | Menarche |
| Kandhal & Miller, Minerva Psichiatrica, 2014 | Review – systematic literature search, narrative review |
| Malcova, Eur J Pediatr, 2006 | Risk estimates is missing, included only median and 25/75^th^ percentile for cases and controls |
| Manuck, AJOG Suppl, 2014 | Abstract |
| Marshall, Diabetic Medicine, 2004 | No information on birthweight categories |
| Mehta, Am J Obstet Gynecol, 2011 | Wrong outcome |
| Meeuvisse, Läkartidningen, 1998 | Swedish text only, obstetrical and perinatal complications |
| Newton, J Pediatr, 2017 | Nonalcoholic fatty liver disease in children |
| Ong, Hormone Research, 2006 | Commentary, no data (obesity) |
| Palatianou, Horm Metab Res, 2014 | No risk estimates |
| Parsons, Int J Obes, 1999 | Wrong outcome |
| Pessah, (Book-chapter) 2008 | Book chapter, not systematic search |
| Pettitt, Current Diabetes Report, 2003 | Review, not systematic search |
| Polanska and Jarosz-Chobot, 2006 | Wrong outcome ( parental age and birth order as risk factors for childhood diabetes) |
| Ponsonby, Pediatric Diabetes, 2011 | No risk estimates |
| Potter, J Epidemiol Community Health, 2013 | Wrong outcome (Predicting adult obesity from measures in earlier life (10-11 yrs) |
| Rami, Eur J Pediatr, 1999 | Wrong outcome (the effects of lactation, cow’s milk feeding, immunization) |
| Ratnalingham, Placenta, 2017 | Review, not systematic search, narrative |
| Ruiz-Narvaez, Diabetes Care, 2014 | Included in SR by Zhao 2018 and Knop 2018 |
| Sadauskaite-Kuehne, Diabetes Metab Res Rev, 2004 | Wrong outcome (lactation). No information on the association between birthweight/LGA and diabetes |
| Sipetic, Eur J Public Health, 2005 | No information on birthweight/LGA in association with risk for diabetes. |
| Song, (abstract) 2016 | Wrong outcome |
| Suder and Chrzanowska, J Biosoc Sci, 2015 | Abdominal obesity risk factors in children & adolescents |
| Stene and Gale, Diabetologica, 2013 | Narrative review, no information about literature search |
| Svensson, Eur J Epidemiology, 2005 | No information on the association between birthweight/LGA and diabetes |
| Tian, Eur J Endocrinology, 2006 | Included adults only |
| Trandafir & Temneanu, J Medicine and Life, 2016 | Narrative review |
| Visalli, Arch Dis Child, 2003 | No information on the association between birthweight/LGA and diabetes |
| Von Bonsdorff, AGE, 2012 | No categories for exposure |
| Waldhoer, Pediatr Diabetes, 2008 | Wrong outcome (length and BMI). No information on the association between birthweight/LGA and diabetes |
| Yarbrough, Diabetes Care, 1998 | Metabolic syndrome in postmenopausal women |
| Zimmermann, Diabetes, 2015 | Included in Knop 2018 |
