# Supplementary material for: The Association Between High Birth Weight and Long-Term Outcomes—Implications for Assisted Reproductive Technologies: A Systematic Review and Meta-Analysis
Source: Front Pediatr. 2021 Jun 23;9:675775. doi: 10.3389/fped.2021.675775 (PMC8260985; doi:10.3389/fped.2021.675775)
Supplement: Supplementary file 1 [file Data_Sheet_1.zip › Supplementary Table 3 4 Quality assessment .nan.lbr.21.09.20, CB 200922, 200924 CBA╠èM, 210220 A╠èM.docx]

**Supplementary Table 3.4 Quality assessment according to Robins-I for type 1**

**diabetes and type 2 diabetes in children/adults.**

| **Type 1 diabetes** | |
| --- | --- |
| **Dominans of bias (Bock, 1994)** | **Risk of bias** |
| Bias due to confounding | Moderate |
| Bias in the selection of participants into the study | Low |
| Bias in the classification of interventions | Low |
| Bias due to deviations from intended interventions | Moderate |
| Bias due to missing data | Low |
| Bias in the measurement of outcome | Moderate |
| Bias in the selection of reported result | No information |
| **Overall risk of bias** | **Serious** |
|  | |
| **Dominans of bias (Borras, 2011)** | **Risk of bias** |
| Bias due to confounding | Moderate |
| Bias in the selection of participants into the study | Low |
| Bias in the classification of interventions | Moderate |
| Bias due to deviations from intended interventions | Serious |
| Bias due to missing data | Low |
| Bias in the measurement of outcome | Low |
| Bias in the selection of reported result | Moderate |
| **Overall risk of bias** | **Serious** |
|  | |
| **Dominans of bias (Cardwell, 2005)** | **Risk of bias** |
| Bias due to confounding | Low |
| Bias in the selection of participants into the study | Low |
| Bias in the classification of interventions | Moderate |
| Bias due to deviations from intended interventions | Moderate |
| Bias due to missing data | Low (8% missing data) |
| Bias in the measurement of outcome | Low |
| Bias in the selection of reported result | Low |
| **Overall risk of bias** | **Moderate** |
|  | |
| **Dominans of bias ( Goldrace, 2018)** | **Risk of bias** |
| Bias due to confounding | Low |
| Bias in the selection of participants into the study | Low |
| Bias in the classification of interventions | Low |
| Bias due to deviations from intended interventions | Low |
| Bias due to missing data | Low |
| Bias in the measurement of outcome | Moderate |
| Bias in the selection of reported result | Low |
| **Overall risk of bias** | **Moderate** |
|  |  |
| **Dominans of bias (Haynes, 2007)** | **Risk of bias** |
| Bias due to confounding | Low |
| Bias in the selection of participants into the study | Low |
| Bias in the classification of interventions | Low |
| Bias due to deviations from intended interventions | Low |
| Bias due to missing data | Low |
| Bias in the measurement of outcome | Moderate |
| Bias in the selection of reported result | Low |
| **Overall risk of bias** | **Moderate** |
|  | |
| **Dominans of bias (Hu, 2020)** | **Risk of bias** |
| Bias due to confounding | Low |
| Bias in the selection of participants into the study | Moderate |
| Bias in the classification of interventions | Moderate |
| Bias due to deviations from intended interventions | Low |
| Bias due to missing data | No information |
| Bias in the measurement of outcome | Low |
| Bias in the selection of reported result | Low |
| **Overall risk of bias** | **Moderate** |
|  |  |
| **Dominans of bias (Ievins, 1997)** | **Risk of bias** |
| Bias due to confounding | Serious |
| Bias in the selection of participants into the study | Serious |
| Bias in the classification of interventions | Moderate |
| Bias due to deviations from intended interventions | Moderate |
| Bias due to missing data | No information |
| Bias in the measurement of outcome | Moderate |
| Bias in the selection of reported result | No information |
| **Overall risk of bias** | **Serious** |
|  | |
| **Dominans of bias (Jones, 1999)** | **Risk of bias** |
| Bias due to confounding | Moderate |
| Bias in the selection of participants into the study | Moderate |
| Bias in the classification of interventions | Moderate |
| Bias due to deviations from intended interventions | Moderate |
| Bias due to missing data | Moderate |
| Bias in the measurement of outcome | Low |
| Bias in the selectionof reported result | No information |
| **Overall risk of bias** | **Moderate** |
|  | |
| **Dominans of bias (Khashan, 2015)** | **Risk of bias** |
| Bias due to confounding | Low |
| Bias in the selection of participants into the study | Low |
| Bias in the classification of interventions | Low |
| Bias due to deviations from intended interventions | Low |
| Bias due to missing data | Low |
| Bias in the measurement of outcome | Low |
| Bias in the selection of reported result | Low |
| **Overall risk of bias** | **Low** |
|  | |
| **Dominans of bias (Kuchlbauer, 2014)** | **Risk of bias** |
| Bias due to confounding | Serious |
| Bias in the selection of participants into the study | Serious |
| Bias in the classification of interventions | Serious |
| Bias due to deviations from intended interventions | Moderate |
| Bias due to missing data | Critical |
| Bias in the measurement of outcome | Serious |
| Bias in the selectionof reported result | Low |
| **Overall risk of bias** | **Critical** |
|  | |
| **Dominans of bias (Lawler-Heavner, 1994)** | **Risk of bias** |
| Bias due to confounding | Moderate |
| Bias in the selection of participants into the study | Moderate |
| Bias in the classification of interventions | Serious |
| Bias due to deviations from intended interventions | Serious |
| Bias due to missing data | Serious |
| Bias in the measurement of outcome | Low |
| Bias in the selectionof reported result | Moderate |
| **Overall risk of bias** | **Serious** |
|  | |
| **Dominans of bias (McKinney, 1999)** | **Risk of bias** |
| Bias due to confounding | Moderate |
| Bias in the selection of participants into the study | Moderate |
| Bias in the classification of interventions | Moderate |
| Bias due to deviations from intended interventions | Low |
| Bias due to missing data | Serious |
| Bias in the measurement of outcome | Moderate |
| Bias in the selection of reported result | Moderate |
| **Overall risk of bias** | **Serious** |
|  | |
| **Dominans of bias (Metcalfe and Baum 1992)** | **Risk of bias** |
| Bias due to confounding | Serious |
| Bias in the selection of participants into the study | Serious |
| Bias in the classification of interventions | Moderate |
| Bias due to deviations from intended interventions | Serious |
| Bias due to missing data | Serious |
| Bias in the measurement of outcome | Serious |
| Bias in the selectionof reported result | Moderate |
| **Overall risk of bias** | **Serious** |
|  | |
| **Dominans of bias (Patterson, 1994)** | **Risk of bias** |
| Bias due to confounding | Serious |
| Bias in the selection of participants into the study | Low |
| Bias in the classification of interventions | Moderate |
| Bias due to deviations from intended interventions | moderate |
| Bias due to missing data | Moderate |
| Bias in the measurement of outcome | Moderate |
| Bias in the selectionof reported result | Serious |
| **Overall risk of bias** | **Serious** |
|  | |
| **Dominans of bias (Rosenbauer, 2008)** | **Risk of bias** |
| Bias due to confounding | Low |
| Bias in the selection of participants into the study | Low |
| Bias in the classification of interventions | Moderate |
| Bias due to deviations from intended interventions | Low |
| Bias due to missing data | Low |
| Bias in the measurement of outcome | Moderate |
| Bias in the selection of reported result | Moderate |
| **Overall risk of bias** | **Moderate** |
|  | |
| **Dominans of bias (Stene, 2001)** | **Risk of bias** |
| Bias due to confounding | Low |
| Bias in the selection of participants into the study | Low |
| Bias in the classification of interventions | Low |
| Bias due to deviations from intended interventions | Low |
| Bias due to missing data | Low |
| Bias in the measurement of outcome | Low |
| Bias in the selection of reported result | Low |
| **Overall risk of bias** | **Low** |
|  | |
| **Dominans of bias (Stene and Joner 2004)** | **Risk of bias** |
| Bias due to confounding | Low |
| Bias in the selection of participants into the study | Low |
| Bias in the classification of interventions | Low |
| Bias due to deviations from intended interventions | Low |
| Bias due to missing data | Low |
| Bias in the measurement of outcome | Low |
| Bias in the selection of reported result | Low |
| **Overall risk of bias** | **Low** |
|  | |
| **Dominans of bias (Wadsworth, 1994)** | **Risk of bias** |
| Bias due to confounding | Serious |
| Bias in the selection of participants into the study | Moderate |
| Bias in the classification of interventions | Low |
| Bias due to deviations from intended interventions | Low |
| Bias due to missing data | Moderate |
| Bias in the measurement of outcome | Low |
| Bias in the selection of reported result | Moderate |
| **Overall risk of bias** | **Serious** |
|  | |
| **Dominans of bias** (**Waernbaum, 2019)** | **Risk of bias** |
| Bias due to confounding | Low |
| Bias in the selection of participants into the study | Low |
| Bias in the classification of interventions | Low |
| Bias due to deviations from intended interventions | Loe |
| Bias due to missing data | Low |
| Bias in the measurement of outcome | Low |
| Bias in the selection of reported result | Low |
| **Overall risk of bias** | **Low** |
|  | |
| **Dominans of bias (Wei, 2006)** | **Risk of bias** |
| Bias due to confounding | Low |
| Bias in the selection of participants into the study | Moderate |
| Bias in the classification of interventions | Moderate |
| Bias due to deviations from intended interventions | Moderate |
| Bias due to missing data | Moderate |
| Bias in the measurement of outcome | Low |
| Bias in the selection of reported result | Low |
| **Overall risk of bias** | **Moderate** |

| **Type 2 diabetes** | |
| --- | --- |
| **Dominans of bias (Zhu, 2013)** | **Risk of bias** |
| Bias due to confounding | Low |
| Bias in the selection of participants into the study | Low |
| Bias in the classification of interventions | Low |
| Bias due to deviations from intended interventions | Low |
| Bias due to missing data | Low |
| Bias in the measurement of outcome | Moderate |
| Bias in the selection of reported result | Low |
| **Overall risk of bias** | **Moderate** |
