# Supplementary material for: The Association Between High Birth Weight and Long-Term Outcomes—Implications for Assisted Reproductive Technologies: A Systematic Review and Meta-Analysis
Source: Front Pediatr. 2021 Jun 23;9:675775. doi: 10.3389/fped.2021.675775 (PMC8260985; doi:10.3389/fped.2021.675775)
Supplement: Supplementary file 1 [file Data_Sheet_1.zip › Supplementary Table 3.1 Bias assessment cancer, A╠èM 210220 .docx, 210526.docx]

**Supplementary Table 3.1 Malignancies**

**Bias assessment according to ROBINS-I.**

| **Breast cancer** | |
| --- | --- |
| **Domains of bias (Andersson, 2001)** | **Risk of bias** |
| Bias due to confounding | Low |
| Bias in the selection of participants into the study | Low |
| Bias in the classification of interventions | Low |
| Bias due to deviations from intended interventions | Low |
| Bias due to missing data | Serious |
| Bias in the measurement of outcome | Low |
| Bias in the selection of reported result | Low |
| **Overall risk of bias** | **Serious** |
| **Domains of bias (Ahlgren, 2003)** | **Risk of bias** |
| Bias due to confounding | Low |
| Bias in the selection of participants into the study | Low |
| Bias in the classification of interventions | Low |
| Bias due to deviations from intended interventions | Low |
| Bias due to missing data | Moderate |
| Bias in the measurement of outcome | Low |
| Bias in the selection of reported result | Low |
| **Overall risk of bias** | **Moderate** |
| **Domains of bias (Ahlgren, 2004)** | **Risk of bias** |
| Bias due to confounding | Low |
| Bias in the selection of participants into the study | Low |
| Bias in the classification of interventions | Low |
| Bias due to deviations from intended interventions | Low |
| Bias due to missing data | Moderate |
| Bias in the measurement of outcome | Low |
| Bias in the selection of reported result | Low |
| **Overall risk of bias** | **Moderate** |
| **Domains of bias (Ahlgren, 2007)** | **Risk of bias** |
| Bias due to confounding | Low |
| Bias in the selection of participants into the study | Low |
| Bias in the classification of interventions | Low |
| Bias due to deviations from intended interventions | Low |
| Bias due to missing data | Moderate |
| Bias in the measurement of outcome | Low |
| Bias in the selection of reported result | Low |
| **Overall risk of bias** | **Moderate** |
| **Domains of bias (Barber, 2019)** | **Risk of bias** |
| Bias due to confounding | Low |
| Bias in the selection of participants into the study | Critical |
| Bias in the classification of interventions | Low |
| Bias due to deviations from intended interventions | Low |
| Bias due to missing data | Critical |
| Bias in the measurement of outcome | Low |
| Bias in the selection of reported result | Low |
| **Overall risk of bias** | **Critical** |
| **Domains of bias I Dos Santos, 2004)** | **Risk of bias** |
| Bias due to confounding | Moderate |
| Bias in the selection of participants into the study | Moderate |
| Bias in the classification of interventions | Low |
| Bias due to deviations from intended interventions | Low |
| Bias due to missing data | Moderate |
| Bias in the measurement of outcome | Low |
| Bias in the selection of reported result | Low |
| **Overall risk of bias** | **Moderate** |
| **Domains of bias (Innes, 2000)** | **Risk of bias** |
| Bias due to confounding | Moderate |
| Bias in the selection of participants into the study | Serious |
| Bias in the classification of interventions | Low |
| Bias due to deviations from intended interventions | Low |
| Bias due to missing data | Serious |
| Bias in the measurement of outcome | Low |
| Bias in the selection of reported result | Low |
| **Overall risk of bias** | **Serious** |
| **Domains of bias (Lahmann, 2004)** | **Risk of bias** |
| Bias due to confounding | Serious |
| Bias in the selection of participants into the study | Moderate |
| Bias in the classification of interventions | Low |
| Bias due to deviations from intended interventions | Low |
| Bias due to missing data | Low |
| Bias in the measurement of outcome | Low |
| Bias in the selection of reported result | Low |
| **Overall risk of bias** | **Serious** |
| **Domains of bias (McCormack, 2003)** | **Risk of bias** |
| Bias due to confounding | Low |
| Bias in the selection of participants into the study | Low |
| Bias in the classification of interventions | Low |
| Bias due to deviations from intended interventions | Low |
| Bias due to missing data | Low |
| Bias in the measurement of outcome | Low |
| Bias in the selection of reported result | Low |
| **Overall risk of bias** | **Low** |
| **Domains of bias (Mellemkjær, 2003)** | **Risk of bias** |
| Bias due to confounding | Moderate |
| Bias in the selection of participants into the study | Low |
| Bias in the classification of interventions | Low |
| Bias due to deviations from intended interventions | Low |
| Bias due to missing data | Moderate |
| Bias in the measurement of outcome | Low |
| Bias in the selection of reported result | Low |
| **Overall risk of bias** | **Moderate** |
| **Domains of bias (Michels, 1996)** | **Risk of bias** |
| Bias due to confounding | Moderate |
| Bias in the selection of participants into the study | Low |
| Bias in the classification of interventions | Low |
| Bias due to deviations from intended interventions | Moderate |
| Bias due to missing data | Serious |
| Bias in the measurement of outcome | Low |
| Bias in the selection of reported result | Low |
| **Overall risk of bias** | **Serious** |
| **Domains of bias (Michels, 2006)** | **Risk of bias** |
| Bias due to confounding | Low |
| Bias in the selection of participants into the study | Low |
| Bias in the classification of interventions | Low |
| Bias due to deviations from intended interventions | Low |
| Bias due to missing data | Low |
| Bias in the measurement of outcome | Low |
| Bias in the selection of reported result | Low |
| **Overall risk of bias** | **Low** |
| **Domains of bias ( Mogren, 1999)** | **Risk of bias** |
| Bias due to confounding | Low |
| Bias in the selection of participants into the study | Low |
| Bias in the classification of interventions | Low |
| Bias due to deviations from intended interventions | Low |
| Bias due to missing data | Low |
| Bias in the measurement of outcome | Low |
| Bias in the selection of reported result | Low |
| **Overall risk of bias** | **Low** |
| **Domains of bias (Sanderson, 2002)** | **Risk of bias** |
| Bias due to confounding | Low |
| Bias in the selection of participants into the study | Low |
| Bias in the classification of interventions | Low |
| Bias due to deviations from intended interventions | Low |
| Bias due to missing data | Moderate |
| Bias in the measurement of outcome | Low |
| Bias in the selection of reported result | Low |
| **Overall risk of bias** | **Moderate** |
| **Domains of bias (Troisi, 2013)** | **Risk of bias** |
| Bias due to confounding | Moderate |
| Bias in the selection of participants into the study | Low |
| Bias in the classification of interventions | Low |
| Bias due to deviations from intended interventions | Low |
| Bias due to missing data | Low |
| Bias in the measurement of outcome | Low |
| Bias in the selection of reported result | Low |
| **Overall risk of bias** | **Moderate** |
| **Domains of bias (Titus-Ernstoff, 2002)** | **Risk of bias** |
| Bias due to confounding | Serious |
| Bias in the selection of participants into the study | Serious |
| Bias in the classification of interventions | Low |
| Bias due to deviations from intended interventions | Low |
| Bias due to missing data | Serious |
| Bias in the measurement of outcome | Low |
| Bias in the selection of reported result | Low |
| **Overall risk of bias** | **Serious** |
| **Domains of bias (Vatten, 2002)** | **Risk of bias** |
| Bias due to confounding | Moderate |
| Bias in the selection of participants into the study | Low |
| Bias in the classification of interventions | Low |
| Bias due to deviations from intended interventions | Low |
| Bias due to missing data | Low |
| Bias in the measurement of outcome | Low |
| Bias in the selection of reported result | Low |
| **Overall risk of bias** | **Moderate** |
| **Domains of bias (Vatten, 2005)** | **Risk of bias** |
| Bias due to confounding | Moderate |
| Bias in the selection of participants into the study | Low |
| Bias in the classification of interventions | Low |
| Bias due to deviations from intended interventions | Low |
| Bias due to missing data | Moderate |
| Bias in the measurement of outcome | Low |
| Bias in the selection of reported result | Low |
| **Overall risk of bias** | **Moderate** |
| **Domains of bias (Wu, 2011)** | **Risk of bias** |
| Bias due to confounding | Moderate |
| Bias in the selection of participants into the study | Serious |
| Bias in the classification of interventions | Low |
| Bias due to deviations from intended interventions | Low |
| Bias due to missing data | Serious |
| Bias in the measurement of outcome | Low |
| Bias in the selection of reported result | Low |
| **Overall risk of bias** | **Serious** |
| **CNS tumors** | |
| **Domains of bias (Crump, 2015)** | **Risk of bias** |
| Bias due to confounding | Low |
| Bias in the selection of participants into the study | Low |
| Bias in the classification of interventions | Low |
| Bias due to deviations from intended interventions | Low |
| Bias due to missing data | Low |
| Bias in the measurement of outcome | Low |
| Bias in the selection of reported result | Low |
| **Overall risk of bias** | **Low** |
| **Domains of bias (Emerson, 1991) (195)** | **Risk of bias** |
| Bias due to confounding | Moderate |
| Bias in the selection of participants into the study | Low |
| Bias in the classification of interventions | Low |
| Bias due to deviations from intended interventions | Low |
| Bias due to missing data | Low |
| Bias in the measurement of outcome | Low |
| Bias in the selection of reported result | Low |
| **Overall risk of bias** | **Moderate** |
| **Domains of bias (Greenop, 2014) (190)** | **Risk of bias** |
| Bias due to confounding | Low |
| Bias in the selection of participants into the study | Moderate |
| Bias in the classification of interventions | Low |
| Bias due to deviations from intended interventions | Low |
| Bias due to missing data | Serious |
| Bias in the measurement of outcome | Low |
| Bias in the selection of reported result | Low |
| **Overall risk of bias** | **Serious** |
| **Domains of bias (Johnson, 2016)** | **Risk of bias** |
| Bias due to confounding | Low |
| Bias in the selection of participants into the study | Moderate |
| Bias in the classification of interventions | Low |
| Bias due to deviations from intended interventions | Low |
| Bias due to missing data | Low |
| Bias in the measurement of outcome | Low |
| Bias in the selection of reported result | Low |
| **Overall risk of bias** | **Moderate** |
| **Domains of bias (Kitahara, 2014)** | **Risk of bias** |
| Bias due to confounding | Low |
| Bias in the selection of participants into the study | Low |
| Bias in the classification of interventions | Low |
| Bias due to deviations from intended interventions | Low |
| Bias due to missing data | Low |
| Bias in the measurement of outcome | Low |
| Bias in the selection of reported result | Low |
| **Overall risk of bias** | **Low** |
| **Domains of bias (Mallol-Mesnard, 2008) (192)** | **Risk of bias** |
| Bias due to confounding | Moderate |
| Bias in the selection of participants into the study | Low |
| Bias in the classification of interventions | Fair |
| Bias due to deviations from intended interventions | Low |
| Bias due to missing data | Low |
| Bias in the measurement of outcome | Low |
| Bias in the selection of reported result | Low |
| **Overall risk of bias** | **Moderate** |
| **Domains of bias (McLaughlin, 2009) (191)** | **Risk of bias** |
| Bias due to confounding | Moderate |
| Bias in the selection of participants into the study | Moderate |
| Bias in the classification of interventions | Low |
| Bias due to deviations from intended interventions | Low |
| Bias due to missing data | Low |
| Bias in the measurement of outcome | Low |
| Bias in the selection of reported result | Low |
| **Overall risk of bias** | **Moderate** |
| **Domains of bias (Oksuzyan, 20013) (193)** | **Risk of bias** |
| Bias due to confounding | Moderate |
| Bias in the selection of participants into the study | Low |
| Bias in the classification of interventions | Low |
| Bias due to deviations from intended interventions | Low |
| Bias due to missing data | Low |
| Bias in the measurement of outcome | Low |
| Bias in the selection of reported result | Low |
| **Overall risk of bias** | **Moderate** |
| **Domains of bias (O’Neill, 2015)** | **Risk of bias** |
| Bias due to confounding | Moderate |
| Bias in the selection of participants into the study | Moderate |
| Bias in the classification of interventions | Low |
| Bias due to deviations from intended interventions | Low |
| Bias due to missing data | Low |
| Bias in the measurement of outcome | Low |
| Bias in the selection of reported result | Low |
| **Overall risk of bias** | **Moderate** |
| **Domains of bias (Savitz, 1994)** | **Risk of bias** |
| Bias due to confounding | Moderate |
| Bias in the selection of participants into the study | Serious |
| Bias in the classification of interventions | Low |
| Bias due to deviations from intended interventions | Low |
| Bias due to missing data | Serious |
| Bias in the measurement of outcome | Low |
| Bias in the selection of reported result | Low |
| **Overall risk of bias** | **Serious** |
| **Domains of bias (Schüz, 2001) (196)** | **Risk of bias** |
| Bias due to confounding | Serious |
| Bias in the selection of participants into the study | Low |
| Bias in the classification of interventions | Low |
| Bias due to deviations from intended interventions | Low |
| Bias due to missing data | Moderate |
| Bias in the measurement of outcome | Low |
| Bias in the selection of reported result | Low |
| **Domains of bias (Schüz, 2007)** | **Risk of bias** |
| Bias due to confounding | Moderate |
| Bias in the selection of participants into the study | Serious |
| Bias in the classification of interventions | Low |
| Bias due to deviations from intended interventions | Low |
| Bias due to missing data | Serious |
| Bias in the measurement of outcome | Low |
| Bias in the selection of reported result | Low |
| **Overall risk of bias** | **Serious** |
| **Domains of bias (Spix, 2009)** | **Risk of bias** |
| Bias due to confounding | Moderate |
| Bias in the selection of participants into the study | Low |
| Bias in the classification of interventions | Low |
| Bias due to deviations from intended interventions | Low |
| Bias due to missing data | Low |
| Bias in the measurement of outcome | Low |
| Bias in the selection of reported result | Low |
| **Overall risk of bias** | **Moderate** |
| **Domains of bias (Tettamanti, 2016)** | **Risk of bias** |
| Bias due to confounding | Low |
| Bias in the selection of participants into the study | Low |
| Bias in the classification of interventions | Low |
| Bias due to deviations from intended interventions | Low |
| Bias due to missing data | Low |
| Bias in the measurement of outcome | Low |
| Bias in the selection of reported result | Low |
| **Overall risk of bias** | **Low** |
| **Domains of bias (Tran, 2017)** | **Risk of bias** |
| Bias due to confounding | Moderate |
| Bias in the selection of participants into the study | Low |
| Bias in the classification of interventions | Low |
| Bias due to deviations from intended interventions | Low |
| Bias due to missing data | Low |
| Bias in the measurement of outcome | Low |
| Bias in the selection of reported result | Low |
| **Overall risk of bias** | **Moderate** |
| **Domains of bias (Urayama, 2008) (194)** | **Risk of bias** |
| Bias due to confounding | Moderate |
| Bias in the selection of participants into the study | Low |
| Bias in the classification of interventions | Low |
| Bias due to deviations from intended interventions | Low |
| Bias due to missing data | Low |
| Bias in the measurement of outcome | Low |
| Bias in the selection of reported result | Low |
| **Overall risk of bias** | **Moderate** |
| **Domains of bias (Von Behren, 2003) (189)** | **Risk of bias** |
| Bias due to confounding | Moderate |
| Bias in the selection of participants into the study | Low |
| Bias in the classification of interventions | Low |
| Bias due to deviations from intended interventions | Low |
| Bias due to missing data | Low |
| Bias in the measurement of outcome | Low |
| Bias in the selection of reported result | Low |
| **Overall risk of bias** | **Moderate** |
| **Domains of bias (Yaezel, 1997)** | **Risk of bias** |
| Bias due to confounding | Moderate |
| Bias in the selection of participants into the study | Low |
| Bias in the classification of interventions | Low |
| Bias due to deviations from intended interventions | Low |
| Bias due to missing data | Low |
| Bias in the measurement of outcome | Low |
| Bias in the selection of reported result | Low |
| **Overall risk of bias** | **Moderate** |
| **Hematological malignancies** | |
| **Domains of bias (Cnattingius, 1995)** | **Risk of bias** |
| Bias due to confounding | Moderate |
| Bias in the selection of participants into the study | Low |
| Bias in the classification of interventions | Low |
| Bias due to deviations from intended interventions | Low |
| Bias due to missing data | Low |
| Bias in the measurement of outcome | Low |
| Bias in the selection of reported result | Low |
| **Overall risk of bias** | **Moderate** |
| **Domains of bias (Crump, 2015)** | **Risk of bias** |
| Bias due to confounding | Low |
| Bias in the selection of participants into the study | Low |
| Bias in the classification of interventions | Low |
| Bias due to deviations from intended interventions | Low |
| Bias due to missing data | Low |
| Bias in the measurement of outcome | Low |
| Bias in the selection of reported result | Low |
| **Overall risk of bias** | **Low** |
| **Domains of bias (Groves, 2018)** | **Risk of bias** |
| Bias due to confounding | Moderate |
| Bias in the selection of participants into the study | Low |
| Bias in the classification of interventions | Low |
| Bias due to deviations from intended interventions | Low |
| Bias due to missing data | Low |
| Bias in the measurement of outcome | Low |
| Bias in the selection of reported result | Low |
| **Overall risk of bias** | **Moderate** |
| **Domains of bias (Hjalgrim, 2004)** | **Risk of bias** |
| Bias due to confounding | Moderate |
| Bias in the selection of participants into the study | Low |
| Bias in the classification of interventions | Low |
| Bias due to deviations from intended interventions | Low |
| Bias due to missing data | Low |
| Bias in the measurement of outcome | Low |
| Bias in the selection of reported result | Low |
| **Overall risk of bias** | **Moderate** |
| **Domains of bias (Kaatsch, 1998)** | **Risk of bias** |
| Bias due to confounding | Moderate |
| Bias in the selection of participants into the study | Low |
| Bias in the classification of interventions | Low |
| Bias due to deviations from intended interventions | Low |
| Bias due to missing data | Serious |
| Bias in the measurement of outcome | Low |
| Bias in the selection of reported result | Low |
| **Overall risk of bias** | **Serious** |
| **Domains of bias (Koifman, 2008)** | **Risk of bias** |
| Bias due to confounding | Moderate |
| Bias in the selection of participants into the study | Serious |
| Bias in the classification of interventions | Low |
| Bias due to deviations from intended interventions | Low |
| Bias due to missing data | Serious |
| Bias in the measurement of outcome | Low |
| Bias in the selection of reported result | Low |
| **Overall risk of bias** | **Serious** |
| **Domains of bias (Ma, 2005)** | **Risk of bias** |
| Bias due to confounding | Moderate |
| Bias in the selection of participants into the study | Moderate |
| Bias in the classification of interventions | Low |
| Bias due to deviations from intended interventions | Low |
| Bias due to missing data | Moderate |
| Bias in the measurement of outcome | Low |
| Bias in the selection of reported result | Low |
| **Overall risk of bias** | **Moderate** |
| **Domains of bias (McLaughlin, 2006)** | **Risk of bias** |
| Bias due to confounding | Moderate |
| Bias in the selection of participants into the study | Low |
| Bias in the classification of interventions | Low |
| Bias due to deviations from intended interventions | Low |
| Bias due to missing data | Low |
| Bias in the measurement of outcome | Low |
| Bias in the selection of reported result | Low |
| **Overall risk of bias** | **Moderate** |
| **Domains of bias (Okcu, 2002)** | **Risk of bias** |
| Bias due to confounding | Low |
| Bias in the selection of participants into the study | Low |
| Bias in the classification of interventions | Low |
| Bias due to deviations from intended interventions | Low |
| Bias due to missing data | Low |
| Bias in the measurement of outcome | Low |
| Bias in the selection of reported result | Low |
| **Overall risk of bias** | **Low** |
| **Domains of bias (O’Neill, 2015)** | **Risk of bias** |
| Bias due to confounding | Low |
| Bias in the selection of participants into the study | Moderate |
| Bias in the classification of interventions | Low |
| Bias due to deviations from intended interventions | Low |
| Bias due to missing data | Low |
| Bias in the measurement of outcome | Low |
| Bias in the selection of reported result | Low |
| **Overall risk of bias** | **Moderate** |
| **Domains of bias (Paltiel, 2015)** | **Risk of bias** |
| Bias due to confounding | Low |
| Bias in the selection of participants into the study | Low |
| Bias in the classification of interventions | Low |
| Bias due to deviations from intended interventions | Low |
| Bias due to missing data | Low |
| Bias in the measurement of outcome | Low |
| Bias in the selection of reported result | Low |
| **Overall risk of bias** | **Low** |
| **Domains of bias (Peckham-Gregory, 2016)** | **Risk of bias** |
| Bias due to confounding | Moderate |
| Bias in the selection of participants into the study | Moderate |
| Bias in the classification of interventions | Low |
| Bias due to deviations from intended interventions | Low |
| Bias due to missing data | Low |
| Bias in the measurement of outcome | Low |
| Bias in the selection of reported result | Moderate |
| **Overall risk of bias** | **Moderate** |
| **Domains of bias (Petridou, 1997)** | **Risk of bias** |
| Bias due to confounding | Moderate |
| Bias in the selection of participants into the study | Serious |
| Bias in the classification of interventions | Low |
| Bias due to deviations from intended interventions | Low |
| Bias due to missing data | Low |
| Bias in the measurement of outcome | Moderate |
| Bias in the selection of reported result | Low |
| **Overall risk of bias** | **Serious** |
| **Domains of bias (Petridou, 2015)** | **Risk of bias** |
| Bias due to confounding | Low |
| Bias in the selection of participants into the study | Low |
| Bias in the classification of interventions | Low |
| Bias due to deviations from intended interventions | Low |
| Bias due to missing data | Low |
| Bias in the measurement of outcome | Low |
| Bias in the selection of reported result | Low |
| **Overall risk of bias** | **Low** |
| **Domains of bias (Podvin, 2006)** | **Risk of bias** |
| Bias due to confounding | Moderate |
| Bias in the selection of participants into the study | Low |
| Bias in the classification of interventions | Low |
| Bias due to deviations from intended interventions | Low |
| Bias due to missing data | Low |
| Bias in the measurement of outcome | Low |
| Bias in the selection of reported result | Low |
| **Overall risk of bias** | **Moderate** |
| **Domains of bias (Rangel, 2010)** | **Risk of bias** |
| Bias due to confounding | Medium |
| Bias in the selection of participants into the study | Critical |
| Bias in the classification of interventions | Low |
| Bias due to deviations from intended interventions | Low |
| Bias due to missing data | Critical |
| Bias in the measurement of outcome | Low |
| Bias in the selection of reported result | Low |
| **Overall risk of bias** | **Critical** |
| **Domains of bias (Reynolds, 2002)** | **Risk of bias** |
| Bias due to confounding | Moderate |
| Bias in the selection of participants into the study | Low |
| Bias in the classification of interventions | Low |
| Bias due to deviations from intended interventions | Low |
| Bias due to missing data | Low |
| Bias in the measurement of outcome | Low |
| Bias in the selection of reported result | Low |
| **Overall risk of bias** | **Moderate** |
| **Domains of bias (Robison, 1987)** | **Risk of bias** |
| Bias due to confounding | Moderate |
| Bias in the selection of participants into the study | Low |
| Bias in the classification of interventions | Low |
| Bias due to deviations from intended interventions | Low |
| Bias due to missing data | Serious |
| Bias in the measurement of outcome | Low |
| Bias in the selection of reported result | Low |
| **Overall risk of bias** | **Serious** |
| **Domains of bias (Roman, 2013)** | **Risk of bias** |
| Bias due to confounding | Moderate |
| Bias in the selection of participants into the study | Moderate |
| Bias in the classification of interventions | Low |
| Bias due to deviations from intended interventions | Moderate |
| Bias due to missing data | Low |
| Bias in the measurement of outcome | Low |
| Bias in the selection of reported result | Low |
| **Overall risk of bias** | **Moderate** |
| **Domains of bias (Schyz, 2007)** | **Risk of bias** |
| Bias due to confounding | Moderate |
| Bias in the selection of participants into the study | Serious |
| Bias in the classification of interventions | Low |
| Bias due to deviations from intended interventions | Low |
| Bias due to missing data | Serious |
| Bias in the measurement of outcome | Low |
| Bias in the selection of reported result | Low |
| **Overall risk of bias** | **Serious** |
| **Domains of bias (Smith, 2009)** | **Risk of bias** |
| Bias due to confounding | Moderate |
| Bias in the selection of participants into the study | Moderate |
| Bias in the classification of interventions | Low |
| Bias due to deviations from intended interventions | Low |
| Bias due to missing data | Low |
| Bias in the measurement of outcome | Low |
| Bias in the selection of reported result | Low |
| **Overall risk of bias** | **Moderate** |
| **Domains of bias (Tran, 2017)** | **Risk of bias** |
| Bias due to confounding | Moderate |
| Bias in the selection of participants into the study | Low |
| Bias in the classification of interventions | Low |
| Bias due to deviations from intended interventions | Low |
| Bias due to missing data | Low |
| Bias in the measurement of outcome | Low |
| Bias in the selection of reported result | Low |
| **Overall risk of bias** | **Low** |
| **Domains of bias (Triebwasser, 2016)** | **Risk of bias** |
| Bias due to confounding | Moderate |
| Bias in the selection of participants into the study | Low |
| Bias in the classification of interventions | Low |
| Bias due to deviations from intended interventions | Low |
| Bias due to missing data | Low |
| Bias in the measurement of outcome | Low |
| Bias in the selection of reported result | Low |
| **Overall risk of bias** | **Moderate** |
| **Domains of bias (Westergård, 1997)** | **Risk of bias** |
| Bias due to confounding | Low |
| Bias in the selection of participants into the study | Low |
| Bias in the classification of interventions | Low |
| Bias due to deviations from intended interventions | Low |
| Bias due to missing data | Low |
| Bias in the measurement of outcome | Low |
| Bias in the selection of reported result | Low |
| **Overall risk of bias** | **Low** |
| **Domains of bias** (**Zack, 1991)** | **Risk of bias** |
| Bias due to confounding | Moderate |
| Bias in the selection of participants into the study | Low |
| Bias in the classification of interventions | Low |
| Bias due to deviations from intended interventions | Low |
| Bias due to missing data | Low |
| Bias in the measurement of outcome | Low |
| Bias in the selection of reported result | Low |
| **Overall risk of bias** | **Moderate** |
| **Wilm´s tumor** | |
| **Domains of bias (Crump, 2014)** | **Risk of bias** |
| Bias due to confounding | Low |
| Bias in the selection of participants into the study | Low |
| Bias in the classification of interventions | Low |
| Bias due to deviations from intended interventions | Low |
| Bias due to missing data | Low |
| Bias in the measurement of outcome | Low |
| Bias in the selection of reported result | Low |
| **Overall risk of bias** | **Low** |
| **Domains of bias (Daniels*,* 2008)** | **Risk of bias** |
| Bias due to confounding | Serious |
| Bias in the selection of participants into the study | Critical |
| Bias in the classification of interventions | Low |
| Bias due to deviations from intended interventions | Low |
| Bias due to missing data | Serious |
| Bias in the measurement of outcome | Low |
| Bias in the selection of reported result | Low |
| **Overall risk of bias** | **Critical** |
| **Domains of bias (Heck*,* 2018)** | **Risk of bias** |
| Bias due to confounding | Moderate |
| Bias in the selection of participants into the study | Low |
| Bias in the classification of interventions | Low |
| Bias due to deviations from intended interventions | Low |
| Bias due to missing data | Moderate |
| Bias in the measurement of outcome | Low |
| Bias in the selection of reported result | Low |
| **Overall risk of bias** | **Moderate** |
| **Domains of bias (Heuch*,* 1996)** | **Risk of bias** |
| Bias due to confounding | Moderate |
| Bias in the selection of participants into the study | Low |
| Bias in the classification of interventions | Low |
| Bias due to deviations from intended interventions | Low |
| Bias due to missing data | Low |
| Bias in the measurement of outcome | Low |
| Bias in the selection of reported result | Low |
| **Overall risk of bias** | **Moderate** |
| **Domains of bias (Jepsen*,* 2004)** | **Risk of bias** |
| Bias due to confounding | Serious |
| Bias in the selection of participants into the study | Low |
| Bias in the classification of interventions | Low |
| Bias due to deviations from intended interventions | Low |
| Bias due to missing data | Low |
| Bias in the measurement of outcome | Low |
| Bias in the selection of reported result | Low |
| **Overall risk of bias** | **Serious** |
| **Domains of bias (Lindblad*,* 1992)** | **Risk of bias** |
| Bias due to confounding | Moderate |
| Bias in the selection of participants into the study | Low |
| Bias in the classification of interventions | Low |
| Bias due to deviations from intended interventions | Low |
| Bias due to missing data | Moderate |
| Bias in the measurement of outcome | Low |
| Bias in the selection of reported result | Low |
| **Overall risk of bias** | **Moderate** |
| **Domains of bias (Olshan*,* 1993)** | **Risk of bias** |
| Bias due to confounding | Moderate |
| Bias in the selection of participants into the study | Serious |
| Bias in the classification of interventions | Low |
| Bias due to deviations from intended interventions | Low |
| Bias due to missing data | Serious |
| Bias in the measurement of outcome | Low |
| Bias in the selection of reported result | Low |
| **Overall risk of bias** | **Serious** |
| **Domains of bias (Puumala,*,* 2008)** | **Risk of bias** |
| Bias due to confounding | Moderate |
| Bias in the selection of participants into the study | Low |
| Bias in the classification of interventions | Low |
| Bias due to deviations from intended interventions | Low |
| Bias due to missing data | Low |
| Bias in the measurement of outcome | Low |
| Bias in the selection of reported result | Low |
| **Overall risk of bias** | **Moderate** |
| **Domains of bias** (**Rangel, 2010)** | **Risk of bias** |
| Bias due to confounding | Medium |
| Bias in the selection of participants into the study | Critical |
| Bias in the classification of interventions | Low |
| Bias due to deviations from intended interventions | Low |
| Bias due to missing data | Critical |
| Bias in the measurement of outcome | Low |
| Bias in the selection of reported result | Low |
| **Overall risk of bias** | **Critical** |
| **Domains of bias (Schytz*,* 2001)** | **Risk of bias** |
| Bias due to confounding | Serious |
| Bias in the selection of participants into the study | Critical |
| Bias in the classification of interventions | Serious |
| Bias due to deviations from intended interventions | Low |
| Bias due to missing data | Moderate |
| Bias in the measurement of outcome | Low |
| Bias in the selection of reported result | Moderate |
| **Overall risk of bias** | **Critical** |
| **Domains of bias (Schytz*.,* 2010)** | **Risk of bias** |
| Bias due to confounding | Moderate |
| Bias in the selection of participants into the study | Low |
| Bias in the classification of interventions | Low |
| Bias due to deviations from intended interventions | Low |
| Bias due to missing data | Moderate |
| Bias in the measurement of outcome | Low |
| Bias in the selection of reported result | Low |
| **Overall risk of bias** | **Moderate** |
| **Domains of bias (Smulevich*.,* 1999)** | **Risk of bias** |
| Bias due to confounding | Moderate |
| Bias in the selection of participants into the study | Low |
| Bias in the classification of interventions | Low |
| Bias due to deviations from intended interventions | Low |
| Bias due to missing data | Moderate |
| Bias in the measurement of outcome | Low |
| Bias in the selection of reported result | Low |
| **Overall risk of bias** | **Moderate** |

Ref 189-196 are only presented in Tables
