# Supplementary material for: The Association Between High Birth Weight and Long-Term Outcomes—Implications for Assisted Reproductive Technologies: A Systematic Review and Meta-Analysis
Source: Front Pediatr. 2021 Jun 23;9:675775. doi: 10.3389/fped.2021.675775 (PMC8260985; doi:10.3389/fped.2021.675775)
Supplement: Supplementary file 1 [file Data_Sheet_1.zip › Supplementary Table 3.3 Bias assessment Cardiovascular .docx]

**Supplementary Table 3.3. (Cardio-vascular diseases)**

**Bias assessment according to ROBINS-I**

| **Domains of bias (Azadbakht 2014)** | **Risk of bias** |
| --- | --- |
| Bias due to confounding | Low |
| Bias in the selection of participants into the study | No information |
| Bias in the classification of interventions | Moderate |
| Bias due to deviations from intended interventions | Low |
| Bias due to missing data | No information |
| Bias in the measurement of outcome | Low |
| Bias in the selection of reported result | No information |
| **Overall risk of bias** | **Serious** |

| **Domains of bias (Conen 2010)** | **Risk of bias** |
| --- | --- |
| Bias due to confounding | Low |
| Bias in the selection of participants into the study | Moderate |
| Bias in the classification of interventions | Moderate |
| Bias due to deviations from intended interventions | Moderate |
| Bias due to missing data | Moderate |
| Bias in the measurement of outcome | Serious |
| Bias in the selection of reported result | Low |
| **Overall risk of bias** | **Serious** |

| **Domains of bias (Dong 2017)** | **Risk of bias** |
| --- | --- |
| Bias due to confounding | Low |
| Bias in the selection of participants into the study | Serious |
| Bias in the classification of interventions | Serious |
| Bias due to deviations from intended interventions | Serious |
| Bias due to missing data | No information |
| Bias in the measurement of outcome | Low |
| Bias in the selection of reported result | Low |
| **Overall risk of bias** | **Serious** |
| **Domains of bias (Espineira 2011)** | **Risk of bias** |
| Bias due to confounding | Moderate |
| Bias in the selection of participants into the study | Serious |
| Bias in the classification of interventions | Serious |
| Bias due to deviations from intended interventions | Serious |
| Bias due to missing data | Low |
| Bias in the measurement of outcome | Low |
| Bias in the selection of reported result | Low |
| **Overall risk of bias** | **Serious** |

| **Domains of bias (Ferreira 2018)** | **Risk of bias** |
| --- | --- |
| Bias due to confounding | Serious |
| Bias in the selection of participants into the study | Moderate |
| Bias in the classification of interventions | Low |
| Bias due to deviations from intended interventions | Low |
| Bias due to missing data | NA |
| Bias in the measurement of outcome | Low |
| Bias in the selection of reported result | Low |
| **Overall risk of bias** | **Serious** |

| **Domains of bias (Gunnarsdottir 2002)** | **Risk of bias -** (Low, Moderate, Serious, Critical, No information) |
| --- | --- |
| Bias due to confounding | Low |
| Bias in the selection of participants into the study | Moderate |
| Bias in the classification of interventions | Low |
| Bias due to deviations from intended interventions | Low |
| Bias due to missing data | NA |
| Bias in the measurement of outcome | Low |
| Bias in the selection of reported result | Low |
| **Overall risk of bias** | **Moderate** |

| **Domains of bias (Johnsson 2018)** | **Risk of bias** |
| --- | --- |
| Bias due to confounding | Low |
| Bias in the selection of participants into the study | Critical |
| Bias in the classification of interventions | Low |
| Bias due to deviations from intended interventions | Low |
| Bias due to missing data | Low |
| Bias in the measurement of outcome | Low |
| Bias in the selection of reported result | Low |
| **Overall risk of bias** | **Critical** |
| **Domains of bias (Kuciene 2018)** | **Risk of bias** |
| Bias due to confounding | Low |
| Bias in the selection of participants into the study | Moderate |
| Bias in the classification of interventions | Low |
| Bias due to deviations from intended interventions | Low |
| Bias due to missing data | Low |
| Bias in the measurement of outcome | Low |
| Bias in the selection of reported result | Low |
| **Overall risk of bias** | **Moderate** |
| **Domains of bias (Larsson 2015)** | **Risk of bias** |
| Bias due to confounding | Low |
| Bias in the selection of participants into the study | Moderate |
| Bias in the classification of interventions | Low |
| Bias due to deviations from intended interventions | Low |
| Bias due to missing data | Low |
| Bias in the measurement of outcome | Low |
| Bias in the selection of reported result | Low |
| **Overall risk of bias** | **Moderate** |

| **Domains of bias (Launer 1993)** | **Risk of bias** |
| --- | --- |
| Bias due to confounding | Low |
| Bias in the selection of participants into the study | Serious |
| Bias in the classification of interventions | Low |
| Bias due to deviations from intended interventions | Low |
| Bias due to missing data | Moderate |
| Bias in the measurement of outcome | Low |
| Bias in the selection of reported result | Low |
| **Overall risk of bias** | **Serious** |
| **Domains of bias (Ledo 2018)** | **Risk of bias** |
| Bias due to confounding | Moderate |
| Bias in the selection of participants into the study | Low |
| Bias in the classification of interventions | Low |
| Bias due to deviations from intended interventions | Low |
| Bias due to missing data | Low |
| Bias in the measurement of outcome | Low |
| Bias in the selection of reported result | Low |
| **Overall risk of bias** | **Moderate** |

| **Domains of bias (Li C 2006)** | **Risk of bias** |
| --- | --- |
| Bias due to confounding | Low |
| Bias in the selection of participants into the study | NA |
| Bias in the classification of interventions | Serious |
| Bias due to deviations from intended interventions | Low |
| Bias due to missing data | Low |
| Bias in the measurement of outcome | Low |
| Bias in the selection of reported result | Low |
| **Overall risk of bias** | **Serious** |
| **Domains of bias (Li Y 2013)** | **Risk of bias** |
| Bias due to confounding | Low |
| Bias in the selection of participants into the study | Moderate |
| Bias in the classification of interventions | Low |
| Bias due to deviations from intended interventions | Low |
| Bias due to missing data | NA |
| Bias in the measurement of outcome | Low |
| Bias in the selection of reported result | Low |
| **Overall risk of bias** | **Moderate** |

| **Domains of bias (Perkiömäki 2016)** | **Risk of bias** |
| --- | --- |
| Bias due to confounding | Low |
| Bias in the selection of participants into the study | Moderate |
| Bias in the classification of interventions | Low |
| Bias due to deviations from intended interventions | Low |
| Bias due to missing data | Low |
| Bias in the measurement of outcome | Low |
| Bias in the selection of reported result | Low |
| **Overall risk of bias** | **Moderate** |
| **Domains of bias (Rashid 2019)** | **Risk of bias** |
| Bias due to confounding | Low |
| Bias in the selection of participants into the study | Moderate |
| Bias in the classification of interventions | Serious |
| Bias due to deviations from intended interventions | Low |
| Bias due to missing data | NA |
| Bias in the measurement of outcome | Low |
| Bias in the selection of reported result | Low |
| **Overall risk of bias** | **Serious** |

| **Domains of bias (Schooling 2010)** | **Risk of bias** |
| --- | --- |
| Bias due to confounding | Low |
| Bias in the selection of participants into the study | Serious |
| Bias in the classification of interventions | Low |
| Bias due to deviations from intended interventions | Low |
| Bias due to missing data | Low |
| Bias in the measurement of outcome | Low |
| Bias in the selection of reported result | Low |
| **Overall risk of bias** | **Serious** |
| **Domains of bias (Skilton 2014)** | **Risk of bias** |
| Bias due to confounding | Low |
| Bias in the selection of participants into the study | Moderate |
| Bias in the classification of interventions | Low |
| Bias due to deviations from intended interventions | Low |
| Bias due to missing data | Low |
| Bias in the measurement of outcome | Low |
| Bias in the selection of reported result | Low |
| **Overall risk of bias** | **Moderate** |

| **Domains of bias (Strufaldi 2009)** | **Risk of bias** |
| --- | --- |
| Bias due to confounding | Low |
| Bias in the selection of participants into the study | Moderate |
| Bias in the classification of interventions | Serious |
| Bias due to deviations from intended interventions | Low |
| Bias due to missing data | Low |
| Bias in the measurement of outcome | Low |
| Bias in the selection of reported result | Low |
| **Overall risk of bias** | **Serious** |
| **Domains of bias (Tan 2018)** | **Risk of bias** |
| Bias due to confounding | Low |
| Bias in the selection of participants into the study | Serious |
| Bias in the classification of interventions | Serious |
| Bias due to deviations from intended interventions | Low |
| Bias due to missing data | Low |
| Bias in the measurement of outcome | Low |
| Bias in the selection of reported result | Low |
| **Overall risk of bias** | **Serious** |

| **Domains of bias (Timka 2019)** | **Risk of bias** |
| --- | --- |
| Bias due to confounding | Low |
| Bias in the selection of participants into the study | Moderate |
| Bias in the classification of interventions | Low |
| Bias due to deviations from intended interventions | Low |
| Bias due to missing data | Low |
| Bias in the measurement of outcome | Low |
| Bias in the selection of reported result | Low |
| **Overall risk of bias** | **Moderate** |

| **Domains of bias (Yiu 1999)** | **Risk of bias** |
| --- | --- |
| Bias due to confounding | Low |
| Bias in the selection of participants into the study | Serious |
| Bias in the classification of interventions | Low |
| Bias due to deviations from intended interventions | Low |
| Bias due to missing data | Low |
| Bias in the measurement of outcome | Low |
| Bias in the selection of reported result | Low |
| **Overall risk of bias** | **Serious** |
