# Supplementary material for: The Association Between High Birth Weight and Long-Term Outcomes—Implications for Assisted Reproductive Technologies: A Systematic Review and Meta-Analysis
Source: Front Pediatr. 2021 Jun 23;9:675775. doi: 10.3389/fped.2021.675775 (PMC8260985; doi:10.3389/fped.2021.675775)
Supplement: Supplementary file 1 [file Data_Sheet_1.zip › Supplementary Table 4. AMSTAR Systematic reviews, A╠èM 210220 .docx]

**Supplementary Table 4 High birth weight and LGA and long-term outcome. Quality assessment of Systematic Reviews according to AMSTAR.**

| Author, Year, Country | 1.  A priori design | 2.  Two independent data extractors | 3.  At least two databases | 4.  ”Grey literature” included | 5.  List of included studies | 6.  Characteristics of included studies provided | 7.  Assessment of scientific quality of included studies | 8. Conclusions based on the scientific quality of included studies | 9. Appropriate method to combine findings of studies | 10. Assessment of the likelihood of publication bias | 11. Statement of conflict of interest | Overall quality of the systematic review |
| --- | --- | --- | --- | --- | --- | --- | --- | --- | --- | --- | --- | --- |
| Systematic reviews n=17 | | | | | | | | | | | | |
| Cardwell, 2010, UK | Yes | Yes | Yes | Yes | Yes | Yes | No | No | Yes | No | Yes | LOW |
| Caughey, 2009, USA | Yes | No | Yes | No | Yes | Yes | No | Yes | Yes | No | No | LOW |
| Chu, 2010, Canada | Yes | Yes | Yes | No | Yes | Yes | No | No | Yes | No | No | MEDIUM |
| Dalhaus, 2016, Germany | Yes | Yes | Yes | Yes | Yes | Yes | Yes | Yes | Yes | Yes | Yes | HIGH |
| Davies, 2020, UK | Yes | Yes | No | No | Yes | Yes | Yes | Yes | Yes | Yes | Yes | MEDIUM |
| Georgakis, 2017, Greece | Yes | Yes | Yes | Yes | Yes | Yes | Yes | Yes | Yes | Yes | Yes | HIGH |
| Harder, 2007, Germany | Yes | No | Yes | No | Yes | Yes | No | No | Yes | Yes | Yes | Medium |
| Harder, 2008, Germany | Yes | Yes | Yes | No | Yes | Yes | Yes | Yes | Yes | Yes | Yes | HIGH |
| Harder, 2009, Germany | Yes | No | Yes | Yes | Yes | Yes | No | No | Yes | Yes | No | Medium |
| Harder, 2010, Germany | Yes | Yes | Yes | No | Yes | Yes | Yes | Yes | Yes | Yes | Yes | HIGH |
| Hjalgrim, 2003, Denmark | Yes | Yes | Yes | No | Yes | Yes | Yes | Yes | Yes | Yes | No | HIGH |
| Knop, 2018, China | Yes | Yes | Yes | No | Yes | Yes | Yes | Yes | Yes | Yes | Yes | HIGH |
| Michels, 2006, USA | Yes | No | No | No | Yes | Yes | No | No | Yes | No | No | LOW |
| Wang, 2014, China | Yes | Yes | No | No | Yes | Yes | Yes | Yes | Yes | Yes | Yes | Medium |
| Whincup, 2008, UK | Yes | Yes | Yes | No | Yes | Yes | Yes | Yes | Yes | Yes | No | HIGH |
| Xue, 2007, USA | Yes | No | No | No | Yes | Yes | No | No | Yes | No | No | LOW |
| Zhang, 2013, China | Yes | Yes | Yes | No | Yes | Yes | Yes | Yes | Yes | No | Yes | HIGH |
| Zhao, 2018, China | Yes | Yes | Yes | Yes | Yes | Yes | Yes | No | Yes | Yes | Yes | High |
| Zhou, 2020, China | Yes | Yes | Yes | Yes | Yes | Yes | Yes | No | Yes | Yes | Yes | High |
