# Supplementary material for: The Association Between High Birth Weight and Long-Term Outcomes—Implications for Assisted Reproductive Technologies: A Systematic Review and Meta-Analysis
Source: Front Pediatr. 2021 Jun 23;9:675775. doi: 10.3389/fped.2021.675775 (PMC8260985; doi:10.3389/fped.2021.675775)
Supplement: Supplementary file 1 [file Data_Sheet_1.zip › Supplementary Table I 4 Included studies diab 15.09.20.LBR_NBO, CB 201102.A╠èM 210220 docx.docx]

**Supplementary Table 1.4 Characteristics of included studies with high birth weight and LGA and as exposure: Long term outcomes-type 1 and type 2 diabetes.**

| **Author, year, country** | **Study design**  **Population** | **Study duration**  **(year of publication)** | **Exposure** | **Patients (n)** | **Comment** | **Outcome variables** |
| --- | --- | --- | --- | --- | --- | --- |
| **Systematic reviews/meta-analyses n= 6**  **Type 1 diabetes and type 2 diabetes** | | | | | | |
| Cardwell, 2010,  Ireland | Systematic review Meta-analysis 24 articles from 29 populations Cohort n=5  Case-control n=19 | Published before July 2009  0-19 years | Birthweight: <2500 g, 2500-3000 g, 3000-3500 g, 3500-4000 g, ≥4000 g | Cases n=12 087 Controls n= 5 094 651 | Meta-analysis on unadjusted OR presented for individual studies. Adjustments included in meta-analyses | Type 1 diabetes |
| Harder, 2007,  Germany | Systematic review, Meta-analysis  Cohort n=10 Case-control n=4 | 1966-2005 | Birthweight:  ≤2500 g compared to >4000 g or >4500 g | Cases n=6901 Controls n=125 279 | Studies including adults only (n=12). Studies including men only (n=4) and women only (n=1) Children/adolescents up to 18 years (n=2) studies, both case/control studies | Type 2 diabetes |
| Harder, 2009,  Germany | Systematic review  Meta-analysis  Cohort n=2  Case-control n=10 | 1966-2007  ≤ 20 years of age | >4000 g compared to <4000 g | Cases n=7491 Controls n=2 398 150 |  | Type 1 diabetes  Partly overlapping with Cardwell |
| Knop, 2018, China | Systematic review, Meta-analysis  49 studies  Cohort n=36  Case-control n=8  Cross-sectional n=5 | Published Jan 1966-Oct 2016 | Birth weight >4500 g compared to ≤4500 g | Cases n=43 549  Sample n=4 053 387 | >18 years of age | Type 2 diabetes |
| Whincup, 2008, UK | Systematic review  Meta-analysis  30 studies  Cohort n=24  Case-control n=2 Cross-sectional n=4 | 1950-2007 | Per 1000 g increase  Birth weight >4000 g | Cases n=6 260  Controls n=152 594 | Adults | Type 2 diabetes |
| Zhao, 2018, China | Systematic review Meta-analysis  Cohort n=16 Case-control n=5 | Publication year  1994-2016, both children and adults | Birth weight <2500 g, 2500-4000 g, >4000 g | Cases n=22 341 Controls n=290 824 | 19 studies included adults only  Children/adolescents up to 18 years, 2 studies, both case/control studies | Type 2 diabetes  Partly overlapping with Harder 2007 |
| **Original articles**  **Type 1 diabetes n=22** | | | | | | |
| Bock, 1994,  Denmark | Case-control  Hospital admission  National Patient Registry | 1973-1989  <10 years | Birth weight in groups of 250 gram categories. | Cases n=837  Controls n=837 | All children born 1973-1977 diagnosed IDDM 1978-1989.  Age and sex-matched controls | Perinatal risk factors for type 1 diabetes <10 years |
| Borras, 2011,  Spain | Case-control  Catalan register of Diabetes and the Register of Newborn Screening for Metabolic diseases | 1993-2003  <10 years | LGA defined as birth weight >90^th^ percentile | Cases n=306  Controls n=1224 matched 1:4 by year of birth from the general population |  | Perinatal risk factors for type 1 diabetes |
| Cardwell, 2005,  UK | Cohort | 1979-1986  <15 years | High birth weight  ≥4000g BW by gestational age | Cases n=991  Cohort n=447 663 | BW was converted into SD scores of BW by sex and gestational age by using the 1990 British Growth Standard and divided by quintiles into fifths | Perinatal risk factors for type 1 diabetes |
| Goldacre, 2017.  UK | Cohort | 1998-2012  <12 years | Birth weight categories:  <2500, 2500-2999, 3000-3499, 3500-3999, 4000-5499 g | Cases n=2969  Controls n=3 834 405 |  | Type 1 diabetes |
| Haynes, 2007, Australia | Case-control  Western Australian Children diabetes Register | 1980-2002  <15 years | Birth weight categories:  <3000, 3000-3499, 3500-3999, ≥4000 g | Cases n=840  Controls n=558,633 |  | Type 1 diabetes |
| Ievins, 2007,  UK | Cohort  Hospital records | 1963-1999  <15 years | Birth weight categories:  1000-1999, 2000-2499, 2500-2999, 3000-3499, 3500-3999, >4000 g | Cases n=518  Controls n=292,845 |  | Type 1 diabetes |
| Jones, 1999,  UK | Case-control study | 1965-1987  ≤20 years | LGA: defined as >90^th^ percentile for birth weight | Cases n=315  (160 boys 155 girls)  Controls n=1525  Matching up to eight per case, matched for sex, year and place of delivery | 98% of cases were matched with five or more controls | Type 1 diabetes |
| Khashan, 2015,  Sweden | Cohort  Swedish Medical Birth registry, Swedish patient registry | 1973-2009  up to 15 years | Birth weight categories:  <1500, 1500-2499, 2500-2999, 3000-3999, 4000-5500 g | Cases n=13 944  Controls n=3 610 731 |  | Type 1 diabetes |
| Kuchlbauer, 2014, Germany | Cohort study | 1988-April 2013  children | Birth weight standard deviation scores used for children with diabetes and control group | 3 groups of cases:  G1: 0-4.9 years  G2: 5-9.9 years  G3: 10-20 years  Cases: 1117  Controls: 54 344 | No risk estimates | Diabetes in childhood |
| Lawler-Heavner, 1994, USA | Case-control  Colorado IDDM Registry. Self-administered questionnaires collected birth size and demographic data. | 1978-1988  <18 years | Birth weight categories:  <3000, 3000-3499, 3500-3999, ≥4000 g | Cases n=221  Controls n=197 | Phase one n=98 non-Hispanic whites recruited from Colorado IDDM Registry alive as of 06/01/88  Phase two n=126 an additional random sample from Colorado IDDM Registry alive as of 03/01/90 | Type 1 diabetes |
| McKinney, 1999,  UK | Case-control | 1993-1994  up to 16 years | Birth weight categories:  <2500 g , 2500-< 3500 g,  ≥3500 g | Cases n=196  Controls n=325  Sex-matched controls |  | Type 1 diabetes.  Strongest associations were infections and respiratory difficulties |
| Metcalfe and Baum, 1992, UK | Case-control Postal questionnaires to physician and parents | 1988  up to 15 years | Birth weight categories:  < 2500 g, 2500 to < 4000 g,  ≥4500 g | Cases n=952  National registry reference | Data on BW available in 1100/1335 (82% of families) | Type 1 diabetes |
| Patterson, 1994,  UK | Case-control Perinatal data from Child health system | 1979-1987  up to 15 years | Birth weight categories:  <2500 g, ≥4000 g | Cases n=529  Controls n=2645 |  | Type 1 diabetes |
| Rosenbauer, 2008, Germany | Case-control Nationwide hospital based surveillance (ESPED) | 1992-1995  Pre-school children | Birth weight categories:  <2500, 2500-2999, 3000-3999, ≥4000 g | Cases n=719  Controls n=1735 |  | Type 1 diabetes |
| Stene, 2001,  Norway | Cohort  Medical birth registry  National Childhood Diabetes Registry | 1974-1998  up to 15 years | Birth weight categories:  <2000, 2000-2499, 2500-2999, 3000-3499, 3500-3999, 4000-4499, ≥4500g | Cases n=1824  Cohort n= 1 382 602 |  | Type 1 diabetes |
| Stene and Joner, 2004 Norway | Case-control  Medical birth registry  National Childhood Diabetes Registry | Children born between 1985-2000  up to 15 years | Birth weight categories:  <2500, 2500-2999, 3000-3499, 3500-3999, ≥4000 g | Cases n=545  Controls n=1668 |  | Type 1 diabetes |
| Tai, 1998, Taiwan | Case-control  Diabetes registry | Diagnosis 1984-1993, age <30 years | <3000 g, 3000-3999 g, ≥4000 g | Cases n=117  Control n=193  Controls from classmates and colleagues, matched for age, sex and educational level |  | Type 1 diabetes |
| Wadsworth, 1997,  UK | Case-control British pediatric ass. Surveillance unit | 1988  <5 years | Birthweight as a continuous variable | Cases n=273  Controls n=360 | Information on relevant confounders not found | Type 1 diabetes |
| Waernbaum, 2019,  Sweden | Case-control  Linkage between the Swedish Childhood Diabetes Register, the Swedish Medical Birth Register and National Patient Register | 1973-2013  0-14 years | Birthweight as z score category with the interval 0–1 as reference. | Cases n=14 949  Control n=55 712 |  | Type 1 diabetes |
| Wei, 2006,  Taiwan | Case-control Diabetes screening program in all school-children | 6-18 years in the years 1992-97 | ≤2600 g, 2601-2999 g, 3000-3542 g, 3543-3999 g, ≥4000 g | Cases n=277 Controls n=533 |  | Type 1 diabetes |
| **Type 2 diabetes n=2** | | | | | | |
| Hu, 2020, China | Cohort  Nation-wide population cohort | NA | Four weight categories <2500 g- >4000 g | Cohort n=49 118 |  | Diabetes type 1 and 2 |
| Zhu, 2013,  China | Cross-sectional survey of overweight/obese children in Tianjin. Questionnaire, clinical and laboratory measurements | 2010 children | <2500 g, 2500-4000 g, >4000 g | 3173 children participated Obese/overweight n=903  Diabetes n=2 Impaired fasting glucose n=6 Impaired glucose tolerance n=16 |  | Type 2 diabetes and pre-diabetes in obese/ overweight children |
| LGA, large-for-gestational-age; AGA, appropriate-for-gestational-age; HOMA-IR, homeostasis model assessment-insulin resistance; MS, metabolic syndrome; GDM, gestational diabetes mellitus; LBW, low birth weight; HBW, high birth weight; NBW, normal birth weight | | | | | | |
