# Supplementary material for: The Association Between High Birth Weight and Long-Term Outcomes—Implications for Assisted Reproductive Technologies: A Systematic Review and Meta-Analysis
Source: Front Pediatr. 2021 Jun 23;9:675775. doi: 10.3389/fped.2021.675775 (PMC8260985; doi:10.3389/fped.2021.675775)
Supplement: Supplementary file 1 [file Data_Sheet_1.zip › Supplementary Table I.1. Included studies LGA and Cancer 210207, 210220A╠èM.docx, 210516.docx]

**Supplementary Table 1.1 Characteristics of included studies with LGA and high birth weight as exposure: Long-term outcomes-malignancies.**

| **Author, year, country** | **Study design**  **population** | **Study duration**  **(years)** | **Exposure** | **Patients (n)** | **Comment** | **Outcome variables** |
| --- | --- | --- | --- | --- | --- | --- |
| **Breast cancer**  **Systematic reviews/metaanalyses n=3** | | | | | | |
| Michels and Xue, 2006  USA | Case-control n=16  Cohort n=11  Meta-analysis | 1998-2005 | Five weight categories between <2500 and >4000 g | Cases n=12 301  Controls n=418 269 |  | Breast cancer |
| Xue and Michels, 2007, USA | Case-control n=18  Cohort n=14. Systematic review, Meta-analysis | 1998-June 1 2007 | Five weight categories between <2500 and >4000 g | Cases n=21 845  Controls n=777 627 |  | Breast cancer |
| \| Zhou, 2020, China \| Case/control n=16  Systematic review, meta-anlysis \| Up to 2019 \| Continuous per 500 g \| Cases n=16 000  Controls n=553 644 \|  \| Breast cancer \| \| --- \| --- \| --- \| --- \| --- \| --- \| --- \| | Case/control n=16  Systematic review, meta-analysis | Up to 2019 | Continuous per 500 g | Cases n=16 000  Controls n=553 644 |  | Breast cancer |
| **Breast cancer**  **Original articles n=19** | | | | | | |
| Andersson, 2001, Sweden | Cohort. Midwife records, questionnaires, Swedish cancer registry and regional cancer registry | Born 1914-1930. Follow until 1998 | Five weight categories between 1600-5500 g | Cases  n=6  Cohort n= 1080 | All cancers | Breast cancer |
| Ahlgren, 2003, Denmark | Cohort. School health records on birth weight. Danish Cancer Registry and Danish Breast Cancer Cooperative Groups Registry | Born 1930-1975. Followed until 2000 | Seven weight categories between 500-6000 g | Cases n=2334  Cohort n=106 504 | Overlap with Ahlgren 2004. Exclude from meta-analysis | Breast cancer |
| Ahlgren, 2004, Denmark | Cohort. School health records on birth weight and growth data Danish Cancer Registry and Danish Breast Cancer Cooperative Groups Registry | Born 1930-1975. Followed until 2001 | Five weight categories. Median birth weight (kilo) per category | Cases n=3340  Cohort n= 117 415 | Overlap with Ahlgren 2003 | Breast cancer |
| Ahlgren, 2007, Denmark | Cohort. School health records on birth weight, The Danish Civil Registration system and the Danish Cancer Registry | Born 1936-1975. Followed 1968-2003 | Six weight categories 501-5999 g | Cases n=3066  Cohort n >200 000 | Overlap with Ahlgren 2003 and 2004. Not include in meta-analysis for breast cancer. The publication includes all types of malignancies | Breast cancer |
| Barber, 2019, USA | Cohort. Self-reported questionnaire concerning birth weight and cancer. Cancer registries and the National Death Index | Women aged 21-69 years were enrolled 1995. Follow up 1997-2015 | Three weight categories between <2500 and >4000 g | Cases n=601  Cohort n=20 959 |  | Breast cancer |
| I dos Santos, 2004, UK | Cohort, questionnaires, phone interviews | Born 1946, follow up until 2000, age 47-54 at follow up | Four weight categories between  <3000 and≥4000 g | Cases n=59  Cohort n=2176 |  | Breast cancer |
| Innes, 2000, USA | Case-control. Birth registry data and Cancer registry New York State | Women aged 14-37 years and born after 1957 Diagnosis 1978-1995 | Five weight categories between <1500 g and >4500 g | Cases n=484 Controls n=1907  Six nextborn girls per case from same county |  | Breast cancer |
| Lahmann, 2004, Sweden | Case-control. Nested within the Malmö Diet and Cancer cohort study. Birth registries and regional and national cancer registries | Born 1924-1950. Diagnosis 1991-2001. Only women >55 years of age at diagnosis were included in the analysis | Four weight categories between <3000 and >4000 g | Cases n=89  Controls n=238  Aim: 3 aged matched controls per case but 1:1 (n=11) and 1:2 (n=7) |  | Breast cancer |
| McCormack, 2003, Sweden | Cohort. Uppsala birth cohort 1915-1929. Birth registries, Swedish Cancer Registry, Swedish Death Registry | Born 1915-1929. Followed until 2001 | Four weight categories between <3000 and >4000 g | Cases n=359  Cohort n=5358 |  | Breast cancer |
| Mellemkjær, 2001, Denmark | Case-control. Central Population Register, parish registries, midwife records. The Danish Cancer Registry | Women <40 years of age. Born after 1935. Diagnosis 1943-1990 | Four weight categories between <2500 and >4000 g | Cases n=881 Controls n=3423  Age matched controls born immediate before or after birth of cases |  | Breast cancer |
| Michels, 1996, USA | Case-control. Nested within the cohorts of the two Nurse´s Health Studies | Born 1921-1965. Analysis 1992 | Five weight categories between <2500 and >4000 g | Cases n=582  Controls n=1569  Controls 2:1 with no breast cancer from the same cohort. | Overlap with Michels, 2006. | Breast cancer |
| Michels, 2006, USA | Cohort  Nurses Health Study 1 and II.  Questionnaires. Birth weight validated in a sample by questionnaire to mother and by birth certificates. Hospital records of cases | Born 1921-1965,  25-42 years of age. Followed 12 and 26 years | Four weight categories between <5,5 (2495 g) and >8,4 (3810 g) lbs | Cases n=3140  Cohort n=152 608 | Overlap with Michels 1996 | Breast cancer |
| Mogren, 1999, Sweden | Cohort, Birth registry, Cancer Registry | Born 1955-1990, followed to 1994 | Four weight categories between <2500 and ≥4500 g | Cases n=57  Cohort n=248 701 |  | Breast cancer |
| Sanderson, 2002, USA | Case-control, Cancer Registry,  Questionnaires | 25-64 years old, 1996-1998 | Five weight categories between <2500 g and ≥4000 g | Cases n= total 1459, n= 288 interviewed  Control n=1556,  n=350 interviewed  Controls from Populations registry | Premenopausal women interviewed | Breast cancer |
| Troisi, 2013, Sweden, Norway, Denmark | Case-control. National Birth registries and National Cancer registries | Age <20-43 years.  Born 1936-1990. Diagnosis 1979-2010 | Three weight categories between <2500 and >4000 g and LGA yes/no | Cases n=1419  Controls n=14 190  10:1 from medical birth registry same year and country | LGA according to Marsal | Breast cancer |
| Titus-Ernstoff, 2002, USA | Case-control. State cancer registries, telephone interviews | Age 50-79 years. Diagnosis 1992-1994 | Six weight categories between <2500 and >4500 g | Cases n=5659  Controls n=5928  randomly selected from drivers license list or Medicare beneficiary list | 40% missing data on birth weight | Breast cancer |
| Vatten, 2002, Norway | Case-control. Birth records, National Cancer registry. Controls identified through Central Person Registry | Born 1910-1970. Diagnosis between 1959 and 1997 | Four weight categories between <3090 and >3730 g | Cases n=373  Controls n=1150  4:1 Selected from birth records and born consecutively and in the same city as cases and without history of breast cancer | Not in meta-analysis | Breast cancer |
| Vatten, 2005, Norway | Cohort  Birth records linked to daughters ID through Central Person Registry,  The Norwegian Cancer Registry | Born 1920-1958. Followed until 2001. Follow up started >20 years of age | Five weight categories between <3040 and >3840 g | Cases n=312  Cohort n= 16 016 | Partly overlap with Vatten 2002. | Breast cancer |
| Wu, 2011 USA | Case-control  Los Angeles County Cancer Surveillance Program, structured interviews | Age 25-74 years. Diagnosis 1995-2001 or 2003-2006 | Five weight categories between <2500 and >4000 g | Cases n=2259 cases Controls n=2019  selected from neighbourhood to cases Standard algorithm |  | Breast cancer |
| **CNS tumors**  **Systematic reviews** **n=4** | | | | | | |
| Dahlhaus, 2016, Germany | Systematic review | 2007-2016  <15 years of age at diagnosis | >4000 g vs <4000 g  <2500 g vs >2500 g | Cases n=18 845  Controls n=1 838 858 | Partly overlap with Harder 2008 | Astrocytoma  Medulloblastoma  Ependymoma |
| Georgakis, 2017, Greece | Systematic review and meta-analysis | Up to 2018 | >4000 g vs <4000 g  <2500 g vs >2500 g  LGA and SGA vs AGA | Cases  n=53 167 overall  Cases children  n=22 330  Controls n >10 million | Meta-analysis only including child cases | CNS tumors |
| Harder, 2008, Germany | Meta-analysis | 1966-2007  <19 years of age | >4000 g vs <4000 g  <2500 g vs >2500 g | Cases n=4162  Controls n=1 744 802 | Partly overlap with Dalhaus 2016 | Astrocytoma  Medulloblastoma |
| Harder, 2010, Germany | Systematic review and meta-analysis | 1966-2008  0-18 years | >4000 g vs <4000 g  <2500 g vs >2500 g | Cases n=3004  Controls n=2 156 923 |  | Neuroblastoma |
| **CNS tumors**  **Original articles n=18** | | | | | | |
| Crump, 2015, Sweden | Cohort  Swedish Birth Registry, Swedish Cancer Registry | Born 1973-2008. Follow up until 2010 | Three weight categories between <2500 and >4000 g | Cases n=2809  Cohort n=3 571 574 |  | CNS tumors |
| Emerson, 1991, USA (195) | Case-control. Cancer Surveillance System in Western Washington, birth certificates | Born 1965-1986. Follow up 1974-1986 | Two weight categories <4000 g, >4000 g | Controls n=785 Controls n=785 5:1, live born children same year and county |  | CNS tumors |
| Greenop, 2014, Australia (190) | Case-control. Hospital cancer registries. Questionnaires | Diagnosis 2005-2010  Age< 14 years | Three weight categories between <2500 and >4000 g. LGA, AGA and SGA | Cases n=319  Controls n=1079 matched for age, sex and state of residence | 44% of eligible cases and 74% of controls included in the analyses | CNS tumors |
| Johnson, 2016, USA | Cross-sectional. NF1 Patients Initiative. Questionnaire to parents | 2011-2015  <18 years of age | Four weight categories between 567 and  5816 g | Cases n=184  Controls n=422  Non cases |  | CNS tumors |
| Kitahara, 2014, Denmark | Cohort Copenhagen School Health Records, Danish Civil Registration System, Danish Cancer Registry | Born 1930-1989 Follow up 1968-2010  >18 years of age | Continuous per 0.5 kg | Cases n=608  Cohort n=320 425 |  | CNS tumors |
| Mallol-Mesnard, 2008, France (192) | Case-control  National Cancer Registry | Age <15 years  2003-2004 | Three weight categories between <2500 and >4000 g | Cases n=209  Controls n=1681  Random dialing matched for age and sex | 80% of cases and 70% of controls participated | CNS tumors |
| McLaughlin, 2009, USA (191) | Case-control. The New York State Cancer Registry, birth records | 1985-2001  1->18 months of age | Four weight categories between <2500 and >4500 g | Cases n=529  Controls n=12 010  From same birth records matched for age at diagnosis, region and birth year |  | Neuroblastoma |
| Oksuzyan 2013, USA (193) | Case-control. State based cancer registry | 1988-2008  Age <16 years | Five weight categories between 2500 g and >4500 g | Cases n=3308  Controls n=3308 from birth registry  Matched by age and sex | 15% of cases and controls included in analyses due to missing data | CNS tumors |
| O’Neill 2015,  USA+UK | Case-control, cancer registries, birth registries | Age 28 days-14 years | Continous per 500 g and categories with 3000-3490 g as ref | Cases n=3561,  n=5702  Controls n=53 716, n=8106 |  | CNS tumors |
| Savitz, 1994, USA | Case-control, local hospital registry | Age <15 years  1976-1983 | Three weight categories between <2500 and >4000 g | Cases=47  Controls n=212 from random dialing | 70% of cancer cases and 60% of controls accepted participating | CNS tumors |
| Schüz, 2001, Germany (196) | Case-control. German Childhood Cancer Registry, questionnaires | 1993-1997  <15 years of age | Three weight categories between <2500 and >4000 g | Cases n=466  Controls n=2458  From local offices for registration of residents |  | CNS tumors |
| Schüz, 2007, Germany | Case-control. German Childhood Cancer Registry, questionnaires | 1992-94  <15 years of age | Three weight categories between <2500 and >4000 g,  LGA | Cases, n=389  Control, n=2024 |  | CNS tumors |
| Spix, 2009, Germany | Case-control  German childhood cancer registry, telephone interviews | Diagnosis 1993-2003  Age <5 years of age | Three weight categories between <2500 and >4000 g | Cases  Leukemia n=243  Controls n=604  CNS tumors n=102 Controls n=246  Controls matched 3:1 for sex, age and year of diagnosis | Response rate to questionnaires 78% cases, 61% controls | Leukemia  CNS tumors |
| Tettamanti, 2016, Sweden | Cohort Swedish Medical Birth Registry, Swedish Cancer Registry | Born 1973-1995  Study period 1988-2010 | Three weight categories, SGA, AGA, and LGA | Cases n=758  Cohort n=2 032 727 |  | CNS tumors |
| Tran, 2017, USA | Case-control. Hospital Registry, Cancer registry, Death registry, birth certificates. | Born 1945-1989. <15 years of age | Three weight categories between <2500 and >4000 g | Cases  Leucemia n=132  CNS tumors n=75  Controls n=1047  controls from local birth registry matched for yearr of birth, county of residence, sex, ethnicity and mother´s age | Paternal preconceptional exposure to radiation | Leukemia, CNS tumors |
| Urayama, 2007, USA (194) | Case-control. California Cancer Registry, California birth certificates | Age <5 years. Born 1983-1997 Diagnosis 1988-1997 | Four weight categories between <1500 and >4000 g | Cases n=508  Controls n=1016  2:1 same birth certificate files. Matched for date of birth and gender. Mothers Californian residents | Partly overlap with Von Behren 2003 | Neuroblastoma |
| Yaezel, 1997, USA, Australia, Canada | Case-control. Cancer registry, questionnaires | Age <18 years | Two weight categories <4000 g and >4000 g | Cases n= 252.  Controls n=816  random dialing | 50% of cases and 60% of controls participated | CNS tumors |
| Von Behren, 2003, USA (189) | Case-control. California Cancer Registry, California live birth certificates | Age 0-4 years. 1988-1997 | Three birth categories between <2500 and >4000g | Cases n=746  Controls n=1491  2:1 same birth certificate files matched for date of birth and gender. Mothers Californian Residents | Partly overlap with Uruyama, 2008 | CNS tumors |
| **Hematological malignancies.**  **Systematic reviews/metaanalyses n=2** | | | | | | |
| Caughey, 2009, USA | Systematic review and meta-analysis | Age 1-<30 years | Six weight weight categories between <2500 and >4500 g | Cases overall n=16 501  ALL n=10 974  AML n=1832 |  | Leukemia |
| Hjalgrim, 2003, Denmark | Systematic review and meta-analysis | 1962-2002  0-29 years | >4000 g and <4000 g  Trend per 1000 g | Cases, overall n=10 282  ALL n=5281  AML n=963  Combined n=4038  Controls n=not specified |  | Leukemia |
| **Hematologic malignancies**  **Original articles n=29** | | | | | | |
| Cnattingus, 1995, Sweden | Case-control. Swedish Cancer Registry. The Swedish Medical Birth Registry. The Registry of cause of death | 1973-1989  0-16 years of age | Eight weight categories between <1500 and >4500 g | Cases n=613  Controls n=3065 5:1 controls matched by gender, year of birth and month of birth |  | ALL |
| Crump, 2015, Sweden | Cohort Medical birth registry and cancer registry | Born 1973-2008 Follow up until 2010 | SGA, AGA and LGA.  Three weight categories between <2500 and >4000 g | Cases n=1960  Cohort n=3 569 333. |  | ALL |
| Groves, 2018, USA | Case-control. Cancer registries in Kentucky, Arizona and Illinois, birth certificates | Diagnosis 1990-2002  Age <5 years | Three weight categories between <2500 and >4000 g | Cases n=633  Controls n=2755  4:1 and 5:1matched by sex, race, ethnicity county and time of birth |  | ALL |
| Hjalgrim, 2004, Denmark, Sweden, Norway Iceland | Case-control  Medical birth registry, Civil registration, Population based Nordic acute leukemia database | Diagnosis 1984-1999  Age 0-14 years | Eight weight categories between <1500 and >4500 g | Cases n=2204 overall  ALL n=1905  AML n=299  Controls n=10 745  5:1 matched for sex, age and nationality |  | ALL and AML |
| Kaatsch, 1998, Germany | Case-control | Diagnosis 1992-1994  Born ≥1975, age <15 years | Three weight categories between 2500 and >4000 g | Cases  Leukemia n=1184  Lymphomas n=234  Other malignancies  n=940  Controls n=2588  1:1 matched for age, sex and place of residence at diagnosis |  | Leukemia.Non-Hodgkin lymphoma.  Other malignancies |
| Koifman, 2008, Brazil | Case-control  Hospital based registry | Diagnosis 1999-2005  Age 0-21 months | Four weight categories between <2500 and >3999 g | Cases ALL+AML n=201  Controls n=440 non cancer matched by age, sex, and attending same hospital for serious conditions |  | ALL and AML |
| Ma, 2005, USA | Case-control  California State Cancer Registry. Personal interviews | Age 0-14 years  1995-2000 | Three weight categories between <2500 and >4000 g | Cases  ALL n=313  AML n=53  Controls n=460  Randomly selected from birth registry matched for date of birth, gender, race | 85% of eligible cases and 50% of controls participated | AML  ALL |
| McLaughlin, 2006, USA | Case-control  New York State cancer registry, birth certificates | Age <10 years Diagnosed 1985-2001 | Six weight categories between <2500 and >4500 g | Cases n=1070  ALL n=916  AML n=154  Controls n=9686  2:1 from birth cohorts same year New York State |  | AML, ALL |
| Mogren, 1999, Sweden | Cohort, Birth registry, Cancer Registry | Born 1955-1990, followed to 1994  (0-4 years) | Four weight categories between <2500 and ≥4500 g | Cases n=97  Cohort n=248 701 |  | ALL |
| Okcu, 2002, USA | Case-control. Texas State Cancer Registry.  Birth registry | Born 1990-95. Age <5 years. Diagnosed 1995 | Three weight categories between <2500 g and >4000 g | Cases, leukemia total n=104  ALL n=83  Controls n=2669  matched for year of birth |  | Leukemia  ALL |
| O’Neill 2015,  USA+UK | Case-control, cancer registries,  birth registries | Age 28 days-14 years | Continous per 500 g and categories with 3000-3490 g as ref | Cases  Leukemia n=5561, n=7826  Control, n=53 716, n=10 785 |  | Leukemia, |
| Paltiel, 2015, multinational | Cohort, national registries | Age 0-15 years, diagnosed 1959-2009 | Birth weight ≥4.0 kg | Cohort n=112 781 singletons  Leucemia n=115  ALL n=98 |  | Cancer, leukemia, ALL |
| Peckham-Gregory, 2017, USA | Case-control  Texas cancer registry, birth records | Diagnosis 1995-2011  Up to age 16 | Small for gestational age y/n  Large for gestational age y/n | Cases n=374 cases  Controls n=3740  10:1 non cancers born i Texas 1995-2011 |  | Lymphoma |
| Petridou, 1997, Greece | Case-control  National network of hematological clinics, Hospital controls, questionnaires and interviews | Diagnosis 1993-1994  Age 0-14 years | Five weight categories between <2500 and >4000 g | Cases n=153  Controls n=300  2:1 matched for age and sex, hospitalized with acute conditions at the same time as cases |  | Leukemia |
| Petridou, 2015, Sweden | Cohort | Age 0-14 years Diagnosis 1973-2007 | Three weight categories between <2499 and >4000g weight categories. SGA, AGA, LGA | Cases n=684 overall  Non Hodgkin n=515 Hodgkin n=169  Cohort n=3 444 136 |  | Lymphoma |
| Podvin, 2006, USA | Case-control  Washington State Cancer Registry, Birth Registry | 1981-2003  Age<20 years | Three weight catecories between <2500 and >4000 g | Cases  ALL n=376  AML n= 85  Controls n=4980 matched for year of birth |  | ALL  AML |
| Rangel 2010, Brazil | Nested Case-control  Hospital based Registry. Questionnaires and telephone interviews | 1984-2008 | Four weight categories between <2500 and >4000 g | Eligible, total n= 544 Leukemia n= 251  Non Hodgkin n= 261 Wilm´s tumor. n=1200 Controls matched 3:1 for age and sex  Included number of cases total n= 410  Leukemia n=164  Non-Hodgkin n=131  Wilm´s tumor n=115 Controls n=1575 | 547 non responders to questionnaires | Leukemia.  non Hodgkin lymphoma |
| Reynolds, 2002, USA | Case-control  California State Cancer Registry  Birth Registry | 1988-1997  Age <5 years | Three weight categories between <2500 and >4000 g | Cases AML n=240  Controls n=480  Cases ALL n=307  Controls n=614  <2 years  Cases ALL n=1100 Controls n=2198  2-4 years  Controls matched for date of birth and sex | 88% of cases were matched to controls | ALL  AML |
| Robinson, 1987, USA | Case-control  Local hospital registry Minnesota and Mayo clinic | Diagnosis 1969-1987 | Four weight categories between 2000 and >4501 g | Cases n=219 available for analysis. Control group I matched for date of birth 4:1 in county. Control group II matched for date of birth within Minnesota 4:1 | 521 cases in total, however missing data on 302 | ALL |
| Roman, 2013, USA, Germany and UK | Case-control  Treatment centres and interviews | Diagnosis 1989-1996  <15 years of age | Four weight categories between <2500 and >4500 g | Cases n=3922  Controls n=11 823  matched on age at diagnosis, region of residence and sex |  | ALL |
| Savitz, 1994, USA | Case-control, local hospital registry | Age <15 years  1976-1983 | Three weight categories between <2500 and >4000 g | Cases  ALL n=71  Lymphoma n=26  Controls n=212 from random dialing | 70% of cancer cases and 60% of controls accepted participating | ALL, lymphoma |
| Schüz, 2007, Germany | Case-control. German Childhood Cancer Registry, questionnaires | 1992-94  <15 years of age | Three weight categories between <2500 and >4000 g,  LGA | Cases n=389  Control n=2024 |  | ALL, AML, non-Hodgkin lymphoma |
| Smith, 2009, UK | Case-control, cancer and population registries | 1991-1996  <15 years | Three weight categories between <2500 and >4000 g | Cases n=1632  Controls n=3264 |  | Leukemia (ALL, AML) |
| Spix, 2009, Germany | Case-control  German childhood cancer registry, telephone interviews | Diagnosis 1993-2003  Age <5 years of age | Three weight categories between <2500 and >4000 g | Cases  Leukemia n=243  Controls n=604  CNS tumors n=102 Controls n=246  Controls matched 3:1 for sex, age and year of diagnosis | Response rate to questionnaires 78% cases, 61% controls | Leukemia  CNS tumors |
| Tran, 2017, USA | Case-control. Hospital Registry, Cancer registry, Death registry, birth certificates. | Born 1945-1989 <15 years of age | Three weight categories between <2500 and >4000 g | Cases  Leukemia n=132  CNS tumors n=75  Controls n=1047  controls from local birth registry matched for yearr of birth, county of residence, sex, ethnicity and mother´s age | Paternal preconceptional exposure to radiation | Leukemia |
| Triebwasser, 2016, USA | Case-control  California Cancer registry, birth records. | Born 1978-2009. Diagnosis 1988-2011  0-19 years of age | Three weight categories between <2500 and >4000 g | Cases n=1216  Controls n=4485 matched 4:1on month and year of birth, sex and race/ethnicity |  | Hodgkin lymphoma |
| Westergaard, 1997, Denmark | Cohort. Danish Civil Registration and Danish Cancer Registry | Age<15 years. From 1968-1992 | Six weight categories between <2510 and >4510 g | Cohort n= 1 975 584 |  | ALL  AML |
| Yaezel, 1997, USA, Australia, Canada | Case-control.  Cancer registry, questionnaires | Age <18 years | Two weight categories <4000 and >4000 g | Cases  ALL n=1284  AML n=185  Non Hodgkin lymphoma n=190  Control n=816 random dialing | 50% of cases and 60% of controls participated | ALL  AML  Non-Hodgkin lymphoma |
| Zack 1991, Sweden | Case-control. Medical birth registry, National cancer registry, Cause of death registry | Born 1973-1984 | Continous per 100 g increase | Cases n=411  Controls n=2055 matched 5:1 for sex and month and year of birth |  | Leukemia |
| **Wilm’s tumor**  **Systematic reviews/metaanalyses n=1** | | | | | | |
| Chu, 2010, Canada | Systematic review  11 studies (cohort, case-control), meta-analysis | Age 0-15 (18) years | High birth weight (>4000 g) vs normal weight (2500-4000 g) | Cases n >6000  Controls n >1 730 822 |  | Wilm’s tumor |
| **Wilm´s tumor**  **Original articles n=14** | | | | | | |
| Crump, 2014, Sweden | Cohort  Swedish Cancer Registry, Medical Birth Registry | Born 1973-2008 | Three weight categories between <2500 and >4000 g | Cases n=443  Cohort n=3 571 574 |  | Wilm’s tumor |
| Daniels, 2008, USA | Case-control  National Wilm’s Tumour study group (NWTSG) | <16 years of age Diagnosed 1999-2002 | Three weight categories between <2500 and >4000 g | Cases n=521  Controls n=517  matched by age at diagnosis and geographic region |  | Wilm’s tumor |
| Heck, 2019, Denmark | Case-control  Danish Cancer Registry, Population Registry, Medical Birth Registry | <20 years of age  From 1968, end date not given | Three weight categories between <2500 and >4000 g | Cases n=217  Controls n= 4340  matched by sex and age |  | Wilm’s tumor |
| Heuch, 1996, Norway | Cohort  Norwegian Birth Registry, norwegian Cancer Registry | 1967-1992  Age 0-14 years | Four weight categories between <3000 g and >4000 g | Cohort n= 1 489 297 |  | Wilm´s tumor |
| Jepsen, 2004, Denmark | Case-control  Danish birth registry and Danish Cancer Registry | Diagnosis between 1973 and 1993. Age 0-14 years | Four weight categories between <3500 g and >4500 g | Cases n=126  Controls n=1260 matched for gender and year of birth |  | Wilm´s tumor |
| Lindblad, 1992, Sweden | Case-control (nested)  Swedish Cancer Registry, Medical Birth Registry | Born 1973-1984 Registered i Cancer registry through 1984 | >4000 g vs <4000 g | Cases n=110  Controls n=550  Matched by sex and date of birth 5:1 |  | Wilm’s tumor |
| Olshan, 1993, USA | Case-control  National Wilms Tumor Study Group. Interviews | 1984-1986  Age<15 years | Five weight categories between <2500 and >4501 g | Cases n=612  Controls n= 233  Random digit dialing.  Matched for age and geographic area | Birthweight available for 22% of cases and 68% of controls | Wilm´s tumor |
| O’Neill 2015,  USA+UK | Case-control, cancer registries, birth registries | Age 28 days-14 years | Continous per 500 gand categories with 3000-3490 g as ref | Cases n=1129, n=1515  Controls n=53 716, n=2072 |  | Wilm’s tumor, |
| Puumala, 2008, USA | Case-control  Cancer Registry in Minnesota, Birth Registry | Age <14 years  1988-2004 | Weight categories between  <2500 and >4000 g | Cases n=138  Controls n=8752 |  | Wilm´s tumor |
| Rangel, 2010, Brazil | Nested Case-control  Hospital based Registry. Questionnaires and telephone interviews | 1984-2008 | Four weight categories between <2500 and >4000 g | Eligible, total n= 544 Leukemia n= 251  Non Hodgkin n= 261 Wilm´s tumor. n=1200 Controls matched 3:1 for age and sex  Included number of cases total n= 410  Leukemia n=164  Non-Hodgkin n=131  Wilm´s tumor n=115 Controls n=1575 | 547 non responders to questionnaires  70% leukemia  48% non Hodgkin  29% Wilms tumor | Wilm’s tumor |
| Schüz, 2001, Germany | Case-control  German Child Cancer Registry  Questionnaires and interviews | <10 years of age Diagnosed 1988-1994 | Three weight categories between <2500 and >4000 g | Cases n=177  Controls n=2006 from local offices of registration of residents matched by gender and year of birth |  | Wilm’s tumor |
| Schüz, 2011, Denmark, Sweden, Finland, Norway | Case-control, National Cancer Registries | 0-14 years of age  Diagnosed 1985-2006 | Seven weight categories between  <2.0 and ≥ 4.5 kg  LGA vs AGA | Cases n=690  Controls n=3298 from national population registries matched by birth month and year, sex and country |  | Wilm’s tumor |
| Smulevich, 1999, Russia | Case-control  Moscow Cancer Register, Pediatric Polyclinic | 1986-1988  Age <14 years, | <4000 and >4000 g | Cases n=48  Controls n=96  Matched for age, gender and residency |  | Wilm´s tumor |
| Yaezel, 1997, USA, Australia, Canada | Case-control. Cancer registry, questionnaires | Age <18 years | Two weight categories <4000 g and > 4000 g | Cases n=169  Controls n=816, random dialing | 50% of cases and 60% of controls participated | Wilm´s tumor |

CNS, Central Nervous System; LGA, Large for Gestational Age; AML, Acute Myeloid Leukemia; ALL, Acute Lymphatic Leukemia

Ref 189-196 are only presented in Tables
