# Supplementary material for: The Association Between High Birth Weight and Long-Term Outcomes—Implications for Assisted Reproductive Technologies: A Systematic Review and Meta-Analysis
Source: Front Pediatr. 2021 Jun 23;9:675775. doi: 10.3389/fped.2021.675775 (PMC8260985; doi:10.3389/fped.2021.675775)
Supplement: Supplementary file 1 [file Data_Sheet_1.zip › Supplementary Table I.2. Included studies psychiatry 210207 A╠èM,210220.docx]

**Supplementary Table 1.2. Characteristics of included studies with LGA and high birth weight as exposure: Long term outcomes-psychiatric and cognitive disorders**

| **Author, year, country** | **Study design**  **population** | **Study duration (years)** | **Exposure** | **Patients**  **(n)** | **Comment** | **Outcome** | **Variables** |
| --- | --- | --- | --- | --- | --- | --- | --- |
| **Psychiatric and cognitive disorders** | | | | | | | |
| **Systematic reviews n=1** | | | | | | | |
| Davies, 2020, UK | 152 studies  Cohort n=  Case control n=? | 1977-2019 | Birth weight <2000 g, < 2500 g, 2500-3999 g and >4000 g | Not stated |  | Psychosis |  |
| **Psychiatric and cognitive disorders** | | | | | | | |
| **Original articles n=31** | | | | | | | |
| Alati, 2009, Australia | Cohort  Mater University Study of Pregnancy (MUSP) | Children born 1981-1984 | Birth weight  Five quintiles  Quintile 1=lowest birth weight  Quintile 5=highest birth weight | Cases= the whole cohort  Cohort n=4971 |  | Anxious depressive symptoms, social problem symptoms at the age of 14 years |  |
| Bergvall, 2006, Sweden | Cohort  The  Swedish Medical Birth Register, the Swedish Conscript  Register, the Population and Housing Census of 1990,  and the Swedish Multi-Generation Register | Children born between 1973-1981 | Birth weight <SDS below the mean birth weight for gestational age=light for gestational age, between -2 and +2 SDS=appropriate weight for gestational age, >2 SDS=heavy for gestational age. | Cases n=35 821  Cohort n=357 768 |  | Intellectual performance in early adulthood |  |
| Buschgens, 2009, the Netherlands | Cohort  The trackingadolescents’ individual lives survey (TRAILS) | 2001-2002 | Birth weight, <2500 g, ≥4500 g | Cases= the whole cohort  Cohort n=2230 |  | Externalizing behaviors: inattention, hyperactivity/  impulsivity aggression, and  delinquency at the age of 10-12 years  Evaluated by TCP, CBCL |  |
| Dawes, 2015, UK | Cohort from UK Biobank resource | Cohort recruited 2006-2010 | Birth weight, altogether 12 categories; lowest <3 percentile birth weight 1690; highest >97 birth weight 5140 g | Cases= the whole cohort  Cohort n=18 819 |  | Hearing, vision, reaction time and IQ |  |
| Duffany, 2020, USA | Cohort  The NYC Longitudinal Study of Early Development | Cohort recruited 1994-2004 | LGA  AGA | Cohort n= 108 348 |  | School performance |  |
| Eide, 2007, Norway | Cohort  The Medical Birth Registry of Norway and the Norwegian Conscript Service | Children born 1967-1979 | Birth weight  ≤1500 g, 1500-1999 g, 2000-2499 g, 2500-2999 g, 3000-3499 g, 3500-3999 g, 4000-4499 g, 4500-4999 g, ≥5000 g | Cases= the whole cohort  Cohort n=317 761 |  | Intellectual performance at age 18 |  |
| Flensborg-Madsen and Mortensen, 2017, Denmark | Cohort  The Copenhagen Perinatal Cohort | Children born 1959-1961 | Birth weight ≤2500, >2500-3000, >3000-3500, >3500-4000, >4000 | Cases= the whole cohort  Cohort n=4 696 |  | Intelligence at three adult ages 19, 28 and 50 |  |
| Gunnell, 2003, Sweden | Cohort  The Swedish Medical Birth Registry, the Military  Service Conscription Registry, the Population and Housing  Censuses of 1970 and 1990, and the Swedish Inpatient  Discharge Register | Males born in 1973-1980 | Birth weight <2500 g, 2500-3000 g, 3000–3500 g, 3500-4000 g, >4000 g | Cases n=80 with schizophrenia  Cases n = 124 with other non-affective psychoses  Cohort n=246 655  Ca |  | Early-adult-onset schizophrenia and other non- affective psychoses |  |
| Haglund and Källen, 2011, Sweden | case control  The Malmö Child Psychiatric Clinic,  the Child and Youth Habilitation Center in Malmö, the Swedish Medical Birth Registry | Children born in 1980-2005, Enrolled in the study 1997–2007 | Birth weight  <2500 g, 2500-4000 g, >4000 g | Cases n=250  Cohort n=68 964:  all children born in the same region during 1980-2005 |  | Autism and Asperger syndrome at the age 2.5-15 years |  |
| Herva, 2008, Finland | Cohort  The Northern  Finland 1966 Birth Cohort Study | Children born in 1966 | Birth weight <1999 g, 2000-2499 g, 2500-2999 g, 3000-3499 g, 3500-3999 g, 4000-4499 g, 4500-4999 g, ≥5000 g | Cases n=1206  Cohort n=8339 |  | Depression at the age of 31 years |  |
| Keskinen, 2013, Finland | Northern Finland 1966 Birth Cohort | Participants born 1966. followed until 2010 | Birthweight 2500g, 2500-4500 g, >4500 g | Cases n=150  Cohort n=10 526 |  | Schizophrenia by the age of 44 years |  |
| Kristensen, 2014, Norway | Cohort  The Norwegian  Armed Forces Personnel Database | Males born at term 1967-1976 | Birth weight. categories: <2000 g, 2000-2499 g; 2500-2999 g; 3000-3499 g; 3500-3999 g; 4000-4499 g; 4500-4999 g;  >5000 g | Cases= the whole cohort  Cohort n=217 746 |  | IQ score at the time of military conscription |  |
| Lahti, 2015, Finland | Cohort. Helsinki Birth Cohort Study, National hospital discharge register, Cause of death register | Participants born 1934–1944 | SGA=birth weight at or below −2 SD of that predicted by gestational age, AGA=between −2 and +2 SD of that predicted by gestational age, LGA=birth weight at or above +2 SD that predicted by gestational age | Cases n=1660  Cohort n=12 597 |  | Severe mental disorders from early to late adulthood |  |
| Leonard, 2008, Australia | Cohort Western  Australian population data, The Midwives’ Notification System, The IDEA (Intellectual Disability  Exploring Answers) Database | Participants born 1983–1992, and alive 2002 | Birth weight  <1000 g, 1000-1499 g, 1500-1999 g, 2000-2499 g, 2500-2999 g, 3000-3499 g, 3500-3999 g, 4000-4499 g, >4500 g | Cases n=2625  Cohort n=217 252 |  | Intellectual disability |  |
| Liuhanen, 2018, Finland | Cohort  Northern Finland 1966 Birth Cohort, Finnish schizophrenia families | Participants born 1966 (study sample), 1940-1976 (replication sample) | Birth weight >4000 g, ≤4000 g  Low or high genetic risk | Cases n=256  Cohort n=12 058  Study sample n=4223, Replication sample n=282, |  | Social anhedonia and schizophrenia |  |
| Lundgren, 2003, Sweden | Cohort  The Swedish Medical Birth Registry, the Military Service Conscription Registry | Individuals born in 1973 -1988;  conscripted 1991-2006 | Birth weight  Low birth weight for gestational age= birth weight <-2 SDS  High birth weight for gestational age=birth weight  >2 SDS. | Cases= the whole cohort  Cohort n=620 834 males |  | Intellectual performance in young adults |  |
| Moilanen, 2010, Finland | Cohort 1966 Northern Finland 1966 Birth cohort | Follow-up more than 30 years | Birth weight;  <2500 g, 2500-4499 g, ≥4500 g | Cases n=111  Cohort n=12 058  Birth weight available for 10 932 in cohort |  | Schizophrenia at the age of 16 and 33-35 years |  |
| Moore, 2012, USA | Cohort  Database of The Office of Statewide Health Planning and Development  (OSHPD) | Children born Jan 1991-Dec 2001;  Follow up until Nov 2006 | Birth weight  SGA (either 5th or 5-10th percentile), AGA (>10th to <90th percentile), LGA (either 90-95th or >95th percentile) | Cases n=20 206  Cohort n=5 979 605 | The youngest member was 4 years and 11 months old. | Autism |  |
| Perquier, 2014, France | Cohort  The E3N study | Women born between 1925-1950 | Birth weight  <2500 g, 2500-4000 g,>4000 g | Cases n=6335  Cohort n=71 412  Females n=41 144 |  | Postmenopausal depression |  |
| Power, 2006 UK | Birth cohort  The Birth Cohort 1958 in England, Scotland and Wales | Males and females born during one week March 1958 | Birth weight  ≤2500 g, 2501–3000 g, 3001–3500 g, 3501–4000 g, >4000 g | Cases= the whole cohort  Cohort n=13 980 |  | Cognitive development -influence of home and learning environments |  |
| Record, 1969, UK | Cohort  Birmingham, live births | Children born in Jan 1950-Sept 1954 | Birth weight  2000-2400 g, 2500-2900 g, 3000-3400 g, 3500-3900 g, 4000-4400 g | Cases= the whole cohort  Cohort n=41 534 |  | Intelligence (verbal reasoning) at 11+ examinations |  |
| Richards, 2001, UK | Cohort.  The 1946 birth cohort | Participants born 1946 | Birth weight  <2510 g, 2510-3000 g, 3010-3500 g, 3510-4000 g, 4010- 5000 g | Cases= the whole cohort  Cohort n=3900 |  | Cognitive function from childhood to middle life measured at ages 8, 11, 15, 26, and 43 years |  |
| Räikkonen, 2013, Finland | Cohort  Helsinki Birth Cohort | Men born during 1934-1944 | Birth weight | Cases=the whole cohort  Cohort n=931 | 4630 were identified from the birth cohort and only 931 participated in testing | Cognitive development at the age of 20.1 and 67.9 years |  |
| Sörensen, 1997, Denmark | Cohort  The Danish Birth Registry, the Fifth Conscription  District of Denmark | Conscripts born between 1973-1975 | Birth weight  ≤2500 g, 2501-3000 g, 3001-3500 g, 3501-4000 g, 4001- 4500 g, >4500 g | Cases= the whole cohort  Cohort n=4300 |  | Cognitive function in young adult life |  |
| Tamai, 2020, Japan | Cohort | Born 2010. Follop up until 5,5 years | Seven birth weight categories < 3 SD- > 3 SD | Cohort n=35 321 |  | Behavioral development and neurodevelopment |  |
| Van Lieshout, 2020, Canada | Cohort  The 2014 Ontario Child Health Study (OCHS) | 2014 Ontario child Health Study | Birth weight  >4000 g, 2500-4000 g | Cases=628  Cohort n=2151 |  | Mental disorders at the age of 12-17 years: MDD, SA, SP, GAD, ODD, CD and ADHD at the age 12-17 years |  |
| van Mil, 2015; The Netherlands | Cohort  The Generation R Study | Pregnant women enrolled 2001-2005 | Birth weight  High birth weight:  >90th percentile >80th percentile | Cases=the whole cohort  Cohort n=6 015 |  | Child attention problems at 6 years of age |  |
| Wegelius, 2011, Finland | Cohort  The Finnish schizophrenia family study |  | Birth weight <2500 g, 2500-2999 g, 3000-3500 g, 3500-4000 g, >4000 g | Cases: offspring with schizophrenia n=135  Cases: offspring with primary psychotic disorder n=225  Cohort n=1051  (from 315 Finnish schizophrenia families) |  | Schizophrenia and primary psychotic disorder |  |
| Wegelius, 2013, Finland | Cohort  The Finnish schizophrenia family study | Persons born 1940-1976; diagnosis of schizophrenia 1969-1998 | Birth weight <2500 g, 2500-2999 g, 3000–3500 g, 3500-4000 g, >4000 g | Cases n=282 subjects with a primary psychotic disorder, 178 of whom had a diagnosis of schizophrenia Cohort n=1051 from 315 families |  | The symptom severity of psychotic disorders |  |
| Yang Y, 2019, China | Cohort  From Xicheng and Huairou districts | Recruitment of participants Jan 2014-Dec 2014 | Birth weight  normal, low, macrosomia | Cases n=1550 724?Cohort n=9295 students | No definition of birth weight categories | Behavioral problems at the age of 6-16 years |  |
| Zhang, 2020, China | Cohort | Born October 2012-September 2013. Follow up 6 months after birth | Three weight categories between <2500 g and >4000 g | Cohort n= 4026 |  | Development quotient and neurodevelopmental delay |  |

ADHD, attention deficit hyperactivity disorder; AGA, appropriate for gestation; CBCL, child behavior checklist; CD, conduct disorder; GAD, generalized anxiety disorder; IQ, intelligence quotient; LGA, large for gestational age; MDD, major depressive disorder; ODD, oppositional defiant disorder; SDS, standard deviation score; SAD, separation anxiety disorder; SGA, small for gestational age; SP, specific phobia; TCP, teacher’s checklist of psychopathology
