# Supplementary material for: The Association Between High Birth Weight and Long-Term Outcomes—Implications for Assisted Reproductive Technologies: A Systematic Review and Meta-Analysis
Source: Front Pediatr. 2021 Jun 23;9:675775. doi: 10.3389/fped.2021.675775 (PMC8260985; doi:10.3389/fped.2021.675775)
Supplement: Supplementary file 1 [file Data_Sheet_1.zip › Supplementary Table I.3. Included studies Cardiovascular,210220A╠èM.docx]

**Supplementary Table 1.3. Characteristics of included studies with LGA and high birth weight as exposure: Long term outcomes- cardio-vascular diseases**

| **Author, year, country** | **Study design**  **Population** | **Study duration**  **(years)** | **Exposure** | **Patients (n)** | **Comment** | **Outcome variables** |
| --- | --- | --- | --- | --- | --- | --- |
| **Cardio-Vascular** | | | | | | |
| **Systematic reviews+ meta-analysis, n=2** | | | | | | |
| Zhang, 2013,  China | SR and meta-analysis  31 studies  Studies without HBW group were excluded | Study duration NA  Singletons born at full-term age  >1 year | High birth weight (HBW) ≥4000 g or >90th percentile for GA  Normal birth weight (NBW) 2500- 4000 g or the 10-90th percentile for GA  Analyses were performed in  Age subgroups but not predefined in Material & Methods   1. Childhood 2. Early adulthood 3. Adults | HBW n=60 345  NBW n=559 979  Total cohort=621 324 | Meta-analysis of observational studies in Epidemiology Group Checklist  Newcastle-Ottawa quality assessment scale | SBP  DBP  HBP |
| Wang, 2014,  China | SR and meta-analysis  27 studies  23 with BW as continuous variable  14 BW as categorical variable | 1995-2013 | High birth weight ≥4000 g  Normal birth weight 2500-4000 g | Cases with CHD:  n=11 218  Total cohort   n=256 699 | Cochrane methodology  Newcastle-Ottawa quality assessment scale | CHD in adults |
| **Cardio-vascular, original articles, n=21** | | | | | | |
| **Blood pressure / Hypertension, n=14** | | | | | | |
| Azadbakht, 2014,  Iran | Cohort Self-administered questionnaire.  Clinical examination | 2009-2010  Age 10-18 years | <2500 g  2500-4000 g  >4000 g | Cohort n=5528 | CASPIAN III study | Blood pressure (SBP, DBP) |
| Dong, 2017,  China | Cross sectional  Questionnaire  Physical examination | 2013  6-18 years | HBW ≥4000 g | High birth weight n=4981  Normal birth weight n=4981 |  | HBP |
| Espineira, 2011, Brazil | Cohort nested in a population-based prospective cohort.  Maternal interviews  Physical examination | Birth year 1978–1979  2001-2003  23–25 years of age | LGA | Cohort n=515 adults nested in a population-based prospective cohort |  | Blood pressure |
| Ferreira, 2018,  Brazil | Cross-sectional school based.  Questionnaire, clinical examination | Study period NA  Age 12-18 years  Mean age 14.68 years (SD 1.6) | Birth weight (g)  <2500, 2500-2999, 3000-3999, ≥4000 | Cohort n=829 adolescents  Cases with HBP  Office blood pressure 8.5% n=70  Home blood pressure 3.8% n=32 |  | Blood pressure (office and home) |
| Gunnarsdotttir, 2002, Iceland | Cohort  Midwife records, questionnaires,  The Heart Association Heart Preventive clinic  Physical examination | Singletons born 1914-1935 and alive in 1967 and age 33- 65 years | Birth weight (kg)  <3.45  >3.45 to 3.75  >3.75 to <4.0  >4.0 | Cohort n=4601  Cases  Hypertension40-47% of women  59-61% of men  Crude numbers NA | Sub-cohort of the ongoing prospective study: The Reykjavik Study of Icelandic Heart Association | HBP  SBP >140 or DBP >90 mmHg. |
| Kuciene, 2018,  Lithuania | Cross-sectional  Medical records. Clinical examination | Singletons  Born 1995-1998  Follow-up 2010-2012  Age 12-15 years | Birth weight 3 categories:  <2500g, 2500-3999, >4000g  LGA:  BW >90th percentile | Cohort n=4598 adolescents  Cases n= 1178 |  | HBP  HBP: average SDP and DBP >90th percentile |
| Launer, 1993, Netherlands | Cohort  Clinical examination | Born 1980  Follow-up age 4 years | Birth weight (g): | Cohort n=374 children | RCT of healthy women in 7^th^, month of uncomplicated pregnancies with high and low sodium intake | SBP |
| Ledo, 2018,  Brazil | Cross sectional.  Questionnaire, official birth document, clinical examination | 2012  Age 6-12 years  Mean 9.5 (SD2.0) | Birth weight (g): <2500, 2500-3999, ≥4000 | Cohort n=719  SBP >90^th^ percentile  n=22  DBP >90^th^ percentile  n=36 |  | HBP >90^th^ percentile for DBP and SBP |
| Li C, 2006,  USA | Longitudinal cohort. Questionnaire  Clinical examination | 1994-2000  Age 4-12 years  Follow-up 2-6 years (from pre-puberty to late puberty) | Birth weight(g): <2500, 2500-3999, ≥4000 | Cohort n=98 |  | SBP |
| Li Y, 2013, China | Cohort  Questionnaire  Clinical examination | Singletons born in1993-95, follow-up 2005-6 (age 10-13) and 2011-12  (age 15-18) | Birth weight(g):  Cases: ≥4000  Controls: 2500-4000 | Cohort  Childhood:  n=1415 pairs  Adolescence:  n=1112 |  | Blood pressure |
| Schooling, 2010, China | Longitudinal cohort  Questionnaire and physical examination | Born in Singapore 1950-51  Phase 2: 2005-2006  Phase 3: 2006-2008 Age >50 years | Birth weight (per standard deviation higher birth weight) | Cohort n=18958  Cases:  Men 55.9% (n=2824)  Women 47.2% (n=6564) | Guangzhou Biobank cohort study | HBP (>130/85 mmHg or appropriate medication) |
| Strufaldi, 2009,  Brazil | Cross-sectional  Self-reported questionnaire  Physical examination | 2006  Mean age 8 years  (6-10) | Four birth weight categories: ≤2.90 kg 2.91-3.20 kg 3.21-3.58 kg >3.58 kg | Cohort n=739 children |  | Blood pressure |
| Tan, 2018,  China | Cohort  National, multi-center study  Self-reported questionnaire  Physical examination | 2013  Aged 6-18 years  Mean 10.59 +/-3.23 | Three birth weight groups;  LBW <2.5 kg)  NBW (2.5 kg ≤ birth weight <4.0 kg)  HBW ≥4.0 kg | Cohort n=49 357 children  Cases (High SBP) n=7654  Cases (High DBP) n=4787  Cases (hypertension)  n=9479 | “The Health Lifestyles Interventions, a national multi-center study” | HBP  Blood pressure |
| Yiu, 1999,  US | Cohort  Medical records  Physical examination | Pregnancies from 1959-1965  7-year follow-up  (6-9 years of age) | Birth weight and  SGA = less than the 3^rd^ percentile (<2,325g)  AGA = between the 3^rd^ percentile and the 97^th^ percentile LGA = greater than the 97^th^ percentile (>4,500g) | Cohort n=2958 children | NCPP  Exclusion criteria: Gestational age less than 25 weeks | Blood pressure |
| **Coronary heart disease (CHD), n=1** | | | | | | |
| Rashid, 2019, USA | Longitudinal cohort  Questionnaire, telephone interview, hospital files, local hospital discharge lists and death certificate files | 1987-89 baseline examination  Age 45-64  Mean age 53.9 (SD5.7) singleton and born at term.  Median follow-up 22.8 years | Birth weight and 3 categories (kg):  low ≤2.5,  medium 2.5-4.0  high ≥4.0 | Cohort n= 9820  Cases n = 432 | The ARIC study | HF, all-cause mortality, and myocardial infarction  Definition: first occurrence of a hospitalization with an HF diagnosis according to ICD codes or death certificates with death from HF in any position |
| **Atrial fibrillation /other cardio-vascular risk factors, n=6** | | | | | | |
| Conen, 2010,  US | Prospective longitudinal cohort  Mailed questionnaire, medical records | 1993-2009  Women >45 years  14.5 years follow-up  No cardiovascular disease and AF at baseline | BW categorized into five groups: <2.5, 2.5–3.2, 3.2–3.9, 3.9–4.5 and >4.5 kg. | Cohort n=27 982  Cases (with atrial fibrillation) n=735 | Women health study | AF |
| Johnsson, 2018,  Sweden | Cohort study, matched Uppsala University Hospital. Swedish National Board of Health and Welfare  Clinical examination | Born 1975-79  Follow-up in 2014-15 at age 34-40 | Birth Weight >4500g | Cohort n=644  Response rate 54/644Continous outcome  Matched by BW (within ±1 SDS), age, gender (Swedish National Board of Health and Welfare) |  | Cardiovascular risks  Radial artery intima thickness |
| Larsson, 2015,  Sweden | Cohort  Questionnaire, Swedish Health Care Register, Swedish Inpatient Register, Swedish Cause of Death Register | Men born 1918-52, Women born 1914-48  Follow-up 1998-2009  Age:  Men 45-79,  Women 49-83 | Birth weight (g): <1500, 1500-2499, 2500-3999, 4000-4999, ≥5000 | Cohort n= 53 005  n= 29 551 men  n=23 454 women  Cases n=4202  n=2711 men  n=1491 women |  | AF |
| Perkiömäki, 2016, Finland | Cohort  Questionnaire in pregnancy and at delivery, clinical examination | 2012-2014  Born 1966  Age 46 | Birth weight (min-max 1230-6080) and <2500, 2500-3999, ≥4000 | Cohort  rMSSD n=4078  n=1799 men  n=2279 women  BRS n=1922  n=902 men  n=1020 women | The Northern Birth Cohort (NFBC) 1966 (Järvelin 2004) | Cardiovascular autonomic function:  Vagally-mediated heart rate variability (rMSSD)  Spontaneous baroreflex sensitivity (BRS) |
| Skilton, 2014,  Finland | Cohort  Questionnaire  Physical examination | 1980 (3-18 years)  Follow-up 2001 and 2007  24-45 years born at term | LGA | Cohort n=696 |  | Carotid-intima thickness, brachial flow-mediated dilatation, blood pressure |
| Timpka, 2019,  UK | Longitudinal study  Prospective populations-based birth cohort  Medical records  Physical examination | 1991-1992  Mean follow up 17.7 years  55% women | Birth weight Z-score | Cohort n=1964 | Subset of ALSPAC  (Avon Longitudinal study of parents and children)  372 (18.9%) had missing data on birth weight or other covariables. Multiple imputation was used for missing data. | Cardiac structure, systolic function, diastolic function  (Echocardiographic examination) |

BW, birth weight; HBW, high birth weight; NBW, normal birth weight; GA, gestational age; LGA, large for gestational age; AGA, appropriate for gestational age; SGA, small for gestational age; BMI, body mass index; HBP, high blood pressure; SBP, systolic blood pressure; DBP, diastolic blood pressure; CHD, coronary heart disease; CAD, coronary artery disease; AF, atrial fibrillation; HF, heart failure
