# Supplementary material for: The Association Between High Birth Weight and Long-Term Outcomes—Implications for Assisted Reproductive Technologies: A Systematic Review and Meta-Analysis
Source: Front Pediatr. 2021 Jun 23;9:675775. doi: 10.3389/fped.2021.675775 (PMC8260985; doi:10.3389/fped.2021.675775)
Supplement: Supplementary file 2 [file Table_14.DOC]

|  |  |  |  |
| --- | --- | --- | --- |
| **Breast cancer** | Meta-analysis including 15 cohort and case-control studies | High birth weight  AOR of 1.24 (95% 1.11-1.39) | ⊕⊕⊕O  Moderate |
|  | 3 previous SR/meta-analyses | High birth weight  3/3 found an association |  |
|  |  |  |  |
| **CNS tumours** | Meta-analysis including 15 cohort and case-control studies | High birth weight  AOR 1.15 (95% CI 1.05-1.27)  LGA  AOR 1.09 (95% CI 0.95-1.23) | ⊕⊕⊕O  Moderate certainty |
|  | 4 previous SR/meta-analyses | High birth weight  4/4 found an association |  |
|  |  |  |  |
| **Leukemia** | Meta-analysis including 22 cohort and case-control studies | High birth weight  AOR 1.29 (95% CI 1.20-1.39)  LGA  AOR 1.45 (95% CI 1.10-1.91) | ⊕⊕⊕O  Moderate certainty |
|  | 2 previous SR/meta-analyses | High birth weight  2/2 found an association |  |
|  |  |  |  |
| **Lymphoma** | 1 cohort, 7 case-control studies | High birth weight/LGA  4/8 studies found an association | ⊕⊕OO  Low certainty |
|  |  |  |  |
| **Wilm’s tumour** | Meta-analysis including 11 cohort and case-control studies | High birth weight  AOR 1.68 (95% CI 1.38-2.06)  LGA  AOR 1.77 (95% CI 1.31-2.39) | ⊕⊕⊕O  Moderate certainty |
|  | 1 previous SR/meta-analysis | High birth weight/LGA  1/1 found an association |  |
|  |  |  |  |
| **Schizophrenia** | 6 cohort studies | High birth weight and/or LGA 4/6 studies found an association | ⊕⊕OO  Low certainty |
|  |  |  |  |
| **Depression** | 2 cohort studies | High birth weight/women  2/2 studies found an association | ⊕⊕OO  Low certainty |
|  |  |  |  |
| **Psychiatric disorders in general** | 1 SR/ meta-analysis, 3 cohort studies | Contradictory results | ⊕OOO  Very low certainty |
|  |  |  |  |
| **Autism and behavioural problems** | 1 case.control and 6 cohort studies | LGA  5/7 studies found an association | ⊕⊕OO  Low certainty |
|  |  |  |  |
| **Cognitive development** | 5 cohort studies | High birth weight  5/5 studies found an association (better cognitive function) | ⊕⊕OO  Low certainty |
|  |  |  |  |
| **Intellectual performance** | 8 cohort studies | High birth weight and/or LGA 5/8 studies- no association | ⊕⊕⊕O  Moderate certainty |
|  |  |  |  |
| **Blood pressure** | 1 SR/meta-analysis  14 cohort studies | High birth weight-childhood  An association was found  High birth weight/adulthood  Inverse association  Variable findings | ⊕⊕OO  Low certainty  (association in childhood, inverse association in adulthood) |
|  |  |  |  |
| **Coronary heart disease** | 1 SR/meta-analysis | High birth weight  OR 0.89 (95% CI 0.79-1.01) | ⊕⊕⊕O  Moderate certainty |
|  |  |  |  |
| **Atrial fibrillation** | 6 cohort studies | Variable findings | ⊕OOO  Very low certainty |
|  |  |  |  |
| **Diabetes type 1** | Meta-analysis including 13 cohort and case-control studies | High birth weight  OR 1.15 (95% CI 1.05-1.26)  LGA  OR 1.10 (95% CI 1.03-1.21) | ⊕⊕⊕O  Moderate certainty |
|  | 2 previous SR/meta-analyses | High birth weight  2/2 found an association |  |
|  |  |  |  |
| **Diabetes type 2** | 4 previous SR/meta-analyses | 2/4 found an association | ⊕⊕OO  Low certainty |

LGA, large for gestational age;OR, odds ratio;AOR, adjusted odds ratio; SR, systematic review

| High certainty ⊕⊕⊕⊕ | We are very confident that the true effect lies close to that of the estimate of the effect. |
| --- | --- |
| Moderate certainty  ⊕⊕⊕🌕 | We are moderately confident in the effect estimate: The true effect is likely to be close to the estimate of the effect, but there is a possibility that it is substantially different. |
| Low certainty ⊕⊕🌕🌕 | Confidence in the effect estimate is limited: The true effect may be substantially different from the estimate of the effect. |
| Very low certainty ⊕🌕🌕🌕 | We have very little confidence in the effect estimate: The true effect is likely to be substantially different from the estimate of effect. |
